# Mechanochemical Synthesis of Valproic Acid-Based Hybrids: Antioxidant, Anti-Inflammatory, and In Silico Evaluation

**DOI:** 10.3390/molecules31142535

**Published:** 2026-07-21

**Authors:** Diyana Dimitrova, Iliyan Ivanov, Dimitar Bojilov, Gabriel Marc, Smaranda Oniga, Ovidiu Oniga, Ovidiu Crișan, Stanimir Manolov

**Affiliations:** 1 Department of Organic Chemistry, Faculty of Chemistry, University of Plovdiv, 24 “Tsar Assen” Str., 4000 Plovdiv, Bulgaria; dimitrovadiyana5@gmail.com (D.D.); iiiliyan@abv.bg (I.I.); bozhilov@uni-plovdiv.net (D.B.); 2 Department of Organic Chemistry, Faculty of Pharmacy, “Iuliu Hațieganu” University of Medicine and Pharmacy, 41 Victor Babeș Street, RO-400010 Cluj-Napoca, Romania; marc.gabriel@umfcluj.ro (G.M.); ocrisan@umfcluj.ro (O.C.); 3 Department of Pharmaceutical Chemistry, Faculty of Pharmacy, “Iuliu Hațieganu” University of Medicine and Pharmacy, 41 Victor Babeș Street, RO-400010 Cluj-Napoca, Romania; smaranda.oniga@umfcluj.ro; 4 Department of Therapeutic Chemistry, “Iuliu Hațieganu” University of Medicine and Pharmacy, 12 Ion Creangă Street, RO-400010 Cluj-Napoca, Romania; ooniga@umfcluj.ro

**Keywords:** valproic acid, mechanochemical synthesis, amide derivatives, antioxidant activity, anti-inflammatory activity, deep eutectic solvents, lipophilicity, molecular modeling

## Abstract

Valproic acid is a clinically important therapeutic agent whose biological properties have stimulated the development of structurally modified derivatives with improved pharmacological profiles. In the present study, nine amino–valproic acid hybrids were synthesized by a rapid mechanochemical approach using a planetary ball mill under solvent-minimized conditions. The synthesized compounds were formulated in a newly developed deep eutectic solvent composed of urea and propylene glycol (1:4 molar ratio), which was characterized by solvatochromic and Kamlet–Taft analyses. The biological activities of the derivatives were evaluated by hydrogen peroxide scavenging activity (HPSA), hydroxyl radical-scavenging activity (HRSA), and inhibition of albumin denaturation (IAD) assays. Among the investigated compounds, derivative **3h** exhibited the highest biological activity, showing IC_50_ values of 291 µM and 163 µM in the HPSA and HRSA assays, respectively, and the strongest anti-inflammatory activity in the IAD assay. All synthesized derivatives demonstrated lower IC_50_ values than ibuprofen in the albumin denaturation model. Dose–response relationships were analyzed using four- and five-parameter logistic models, with the 5PL model providing a statistically superior fit to the experimental data. Lipophilicity measurements and in silico studies further supported the favorable physicochemical profile of the synthesized compounds. These results identify compound **3h** as the most promising derivative and demonstrate the potential of mechanochemical synthesis for the preparation of biologically active valproic acid hybrids.

## 1. Introduction

Valproic acid (VPA), also known as 2-n-propylpentanoic acid, is a fatty acid-derived anticonvulsant widely recognized for its effectiveness in treating various conditions, including epilepsy [1] and bipolar disorder [2], and it is also used for migraine prophylaxis [3,4]. The pharmacological effects of VPA arise from its capacity to modulate both glutamatergic and *γ*-aminobutyric acid (GABA) neurotransmission [5]. Apart from these established uses, VPA has shown potential in managing aggressive behaviors in individuals with attention-deficit hyperactivity disorder (ADHD), dystonia, and cognitive impairments [1]. Its functions extend beyond traditional therapeutic applications as VPA acts as an epigenetic modulator through the inhibition of histone deacetylases [6,7]. This unique property has led researchers to explore its possibilities for repurposing in oncology [8,9,10,11,12]. Specifically, VPA is being investigated for its applications in treating various cancers, including breast cancer [13,14], colon cancer linked to diabetes [15], and diffuse intrinsic pontine glioma [16]. Additionally, studies are examining its effects on high-fat-diet-induced hypertension [17] and HIV infection [18]. Combination therapies involving VPA are currently in Phase II clinical trials aimed at evaluating its efficacy as a cancer treatment [19,20,21].

Due to its alleged neuroprotective qualities and ability to reduce oxidative stress, VPA is being studied for the treatment of neurodegenerative diseases, such as Parkinson’s disease (PD) [22] and Alzheimer’s disease (AD) [23]. VPA has both oxidative and antioxidative effects, according to clinical data [24,25,26]. However, recent research highlights its propensity to cause oxidative stress, which is a major factor in cellular damage and the advancement of illness, especially when hepatotoxicity, neurotoxicity, nephrotoxicity, and other systemic negative effects are involved [27,28,29]. The improvement of natural antioxidant defense systems is suggested as the main underlying mechanism of neuroprotection, and the neuroprotective effects of VPA are ascribed, at least in part, to its antioxidant qualities [30].

In a number of preclinical models, VPA has demonstrated notable anti-inflammatory properties. By lowering reactive oxygen species (ROS) and using anti-inflammatory mechanisms, VPA increased survival rates and decreased pulmonary damage in a rat model of acute lung injury brought on by superior mesenteric artery blockage [31]. Additionally, VPA demonstrated anti-inflammatory qualities at the molecular and cellular levels in a mouse model of conjunctival inflammation following surgery [32]. VPA changed cytokine profiles, reduced histopathological damage and blood creatinine levels, and promoted renal recovery by reducing inflammatory reactions in a different study [33] involving rats with renal ischemia–reperfusion [34].

Phenethylamine (PEA; β-phenylethylamine) is an endogenous monoamine alkaloid and trace amine that functions primarily as a monoaminergic neuromodulator, with a secondary role as a neurotransmitter, in the human central nervous system [35]. As an endogenous neuroamine, PEA influences physical energy, mood, and attention [36] and is selectively metabolized by monoamine oxidase B to phenylacetic acid. Reduced concentrations of PEA and its metabolite in the biological fluids of individuals with depression suggest a potential link to mood dysregulation [37]. Evidence indicates that PEA may contribute to the pathophysiology of various psychiatric and neurological conditions, including schizophrenia, attention-deficit/hyperactivity disorder, depression, Parkinson’s disease, epilepsy, and Reye’s syndrome; elucidating these roles necessitates accurate quantification of cerebral trace amine levels [38]. Beyond its neurobiological significance, the phenethylamine scaffold serves as a key pharmacophore in medicinal chemistry for the development of active pharmaceutical agents [37].

Derivatives of PEA have demonstrated diverse biological activities, including antimicrobial [39], anticancer [40], antioxidant [41], and antibacterial properties [42]. Recent studies report that phenethylamine compounds exhibit efficacy against Gram-positive and Gram-negative pathogens, anticancer activity in tumor cell lines, and low toxicity toward healthy cells [43], while ongoing research [44] also explores their potential application in weight reduction therapies [45,46,47].

Tryptamines are naturally widespread indole-containing monoamine alkaloids, with their core compound, tryptamine, being derived from tryptophan decarboxylation and functioning as a neuromodulator and neurotransmitter in mammalian brains [48,49]. Endogenous derivatives such as serotonin and melatonin regulate circadian rhythms, metabolic homeostasis, and excitatory signaling [50]. Melatonin exhibits antioxidant [51], anti-inflammatory [52], and immunomodulatory properties [53], with demonstrated potential in preventing tau protein hyperphosphorylation and shielding Alzheimer’s disease patients from Aβ peptide toxicity [54]. Tryptamine derivatives show therapeutic potential for psychoactive disorders [55], cancer [56], and vascular diseases [57], alongside antimalarial, antimicrobial and antiviral cytotoxic activities [58,59]. These scaffolds serve as precursors to natural products (e.g., psychotrimine, chimonanthine) and yield Schiff bases with antiulcer and NTPDase-inhibitory properties relevant to thrombosis and cancer [60,61,62].

Mechanochemistry has gained importance in medicinal chemistry, especially in a field known as “medicinal mechanochemistry,” which focuses on producing active pharmaceutical ingredients (APIs) and pharmaceutically relevant fragments in an ecologically benign manner. This strategy seeks to produce useful molecules effectively while minimizing their influence on the environment. Numerous effective mechanosyntheses of APIs, such as dantrolene (skeletal muscle relaxant), axitinib (renal cell cancer), ftivazide (anti-tuberculosis), teriflunomide (for multiple sclerosis), and nitrofurantoin (antibacterial), have been documented [63,64,65].

Accordingly, the present work focuses on the mechanochemical synthesis of a new series of VPA derivatives and their comprehensive evaluation. The obtained compounds were investigated for their antioxidant and anti-inflammatory activities, while their toxicity profiles were assessed using in silico approaches. In addition, their lipophilicity was determined both experimentally and computationally to elucidate structure–property relationships. Through this integrated approach, the study aims to provide new insights into the development of valproic acid-based derivatives with improved pharmacological potential and safety profiles.

Deep eutectic solvents (DESs) represent a novel class of “green” solvents that have attracted considerable attention in the pharmaceutical and biomedical fields. They are regarded as a sustainable alternative to conventional organic solvents due to their low toxicity, biodegradability, and ease of preparation. Many contemporary active pharmaceutical ingredients (APIs) are hydrophobic, which limits their solubility and, consequently, their bioavailability. DESs serve as efficient solubilizing media, enhancing the bioavailability of such compounds through complex intermolecular interactions that help maintain molecular stability in solution.

The concept of Therapeutic Deep Eutectic Solvents (THEDESs) represents an innovative approach in which the drug itself functions as a constituent of the solvent system. This transforms the solvent into an active component of the therapeutic formulation, eliminating the need for additional excipients and potentially optimizing the pharmacokinetic profile of the drug [66,67,68]. Considering the growing pharmaceutical relevance of deep eutectic solvents, the present study aimed to develop a novel DES system and evaluate its suitability for in vitro biological applications. To this end, a previously unreported deep eutectic solvent based on urea and propylene glycol (1:4 molar ratio) was synthesized and characterized. The resulting DES was subsequently used as the medium for in vitro biological assessment.

Based on the therapeutic relevance of VPA and the promising biological activities of phenethylamine and tryptamine derivatives, the present work aimed to develop novel valproic acid-based compounds using an environmentally friendly mechanochemical approach. The synthesized derivatives were comprehensively evaluated in terms of their biological activity, lipophilicity, and safety profiles, while a newly developed deep eutectic solvent was employed as a green medium for their in vitro biological assessment.

## 2. Results and Discussion

### 2.1. Synthesis

This article reports the synthesis of hybrid molecules of VPA with variously substituted 2-phenylethylamines **1a**–**e**, **g**, **h**), including veratrylamine **1f** and tryptamine **1i**. Valproic acid was first transformed into the corresponding acid chloride **2** by refluxing in toluene in the presence of thionyl chloride for 2 h. The resulting acid chloride **2** was then used directly in the subsequent step without further purification.

**Scheme 1:**
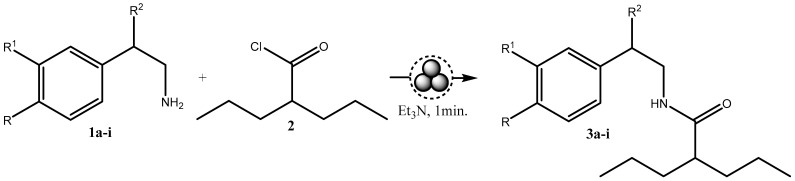
Synthesis of hybrid molecules **3a**–**i**.

The hybrid molecules **3a**–**i** were obtained mechanochemically within one minute. In a 25 mL stainless steel grinding jar, the corresponding amines **1** 2-propylpentanoyl chloride **2** and triethylamine were combined with four 12 mm hardened-steel grinding balls. The mechanochemical synthesis was performed using a Retsch PM 200 planetary ball mill operating at a constant rotational speed of 500 rpm for 1 min. The mechanochemical protocol offers a rapid, operationally simple, and potentially sustainable synthetic route, enabling the preparation of the target VPA hybrids within only 1 min under solvent-minimized conditions (Scheme 1).

Compound **3a** has been previously reported [69], whereas compounds **3b**, **3f**, **3g**, and **3h** are reported here for the first time. Compounds **3c**, **3d**, **3e**, and **3i** were first reported by our research group in our previous study. The starting amine for compound **3f** is veratrylamine or (3,4-dimethoxyphenyl)methanamine and for compound **3i** it is tryptamine (Table 1).

All synthesized hybrid derivatives were successfully identified and structurally characterized using a combination of spectroscopic and spectrometric techniques. The yields of the obtained compounds **3a**–**i** were all above 90% and are presented in Table 1. Their structures were confirmed by ^1^H (Figures S1–S5) and ^13^C (Figures S6–S10) nuclear magnetic resonance (NMR) spectroscopy, infrared (IR) spectroscopy (Figures S11–S15), and high-resolution mass spectrometry (HRMS) (Figures S16–S20). The obtained spectral data were consistent with the proposed molecular structures and confirmed the successful synthesis of the target compounds.

### 2.2. Salvatochromism and Kamlet–Taft Multiparameter Scale

**Figure 1:**
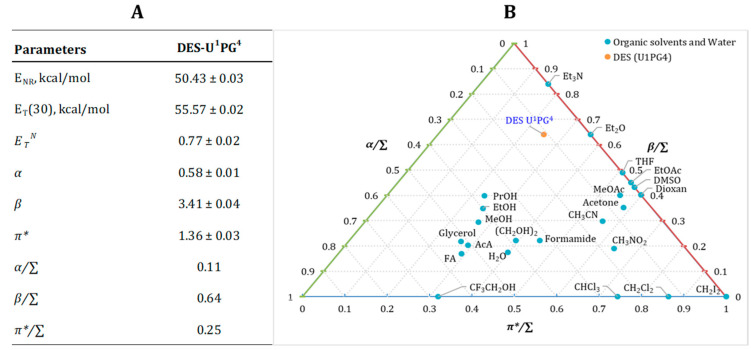
(**A**) Synthesis of solvatochromic parameters and Kamlet–Taft multiparameter scale. (**B**) Comparative evaluation of Kamlet–Taft parameters of newly obtained DES at 298 K with reference organic liquids. The reference normalized values *α*/∑, *β*/∑, and π*/∑ for organic solvents and water are taken from Snyder [70].

The solvatochromic properties of the obtained solvent were evaluated using empirical polarity parameters and Kamlet–Taft parameters (Figure 1A). The E_T_(30) value of 55.57 kcal/mol and the normalized polarity parameter $E_{T}^{N}$ = 0.77 confirm the high polarity of the system. Analysis of the Kamlet–Taft parameters revealed the specific nature of the molecular interactions: basicity (*β* = 3.41) was identified as the dominant factor in the solvatochromic profile, whereas acidity (*α* = 0.58) and polarizability (π* = 1.36) made more moderate contributions. The normalized values (β/∑ = 0.64 compared to α/∑ = 0.11 and $\pi^{*}/\sum$ = 0.25) clearly highlight the role of hydrogen-bonding interactions arising from the presence of urea. The triangular plot (Figure 1B) positions DES-U^1^PG^4^ distinctly away from aprotic solvents and in close proximity to highly polar protic media, further corroborating its strong hydrogen-bonding character and elevated polarity.

Despite its pronounced polarity, DES-U^1^PG^4^ exhibits a unique nanostructured organization that facilitates the efficient solubilization of chemically diverse compounds. The coexistence of microdomains with different physicochemical properties enables the solvent system to adapt dynamically to the solute environment, thereby promoting the dissolution of both polar and nonpolar molecules. The synthesized compounds, together with the corresponding reference standards, were formulated in the prepared DES and subsequently subjected to in vitro biological evaluation.

### 2.3. Investigation of Biological Activity

The biological potential of the synthesized compounds was assessed using three complementary in vitro methods: hydrogen peroxide scavenging activity (HPSA), hydroxyl radical-scavenging activity (HRSA), and inhibition of albumin denaturation (IAD). The results were expressed as IC_50_ values (µM), which served as quantitative measures of antioxidant and anti-inflammatory activity.

#### 2.3.1. Hydrogen Peroxide Scavenging Activity (HPSA)

Reactive oxygen species (ROS), including oxygen-centered radicals, non-radical oxidizing molecules, and singlet oxygen (^1^O_2_), are continuously generated as unavoidable by-products of aerobic metabolism in all living organisms [71]. ROS are characterized by their high reactivity and instability, enabling them to readily interact with numerous biological macromolecules, including proteins, lipids, lipoproteins, and nucleic acids. When generated in excess, ROS induce oxidative stress, leading to cellular and tissue damage associated with aging and the development of various pathological conditions, including atherosclerosis, carcinogenesis, and mutagenesis [72]. Although endogenous antioxidant defense systems effectively counteract the harmful effects of reactive oxygen species, they cannot entirely prevent oxidative damage. Consequently, the supplementation of exogenous antioxidants is of considerable importance for maintaining cellular homeostasis and supporting overall health [73]. However, the use of synthetic antioxidants has become increasingly restricted due to concerns regarding their potential health risks, including toxic effects and damage to DNA and protein structures [74]. In addition to the mechanisms of oxidative stress, inflammatory processes further promote and amplify the generation of ROS. One of the major contributors to inflammation is the formation of superoxide anion radicals, which are also involved in the production of other reactive oxygen species, including hydrogen peroxide (H_2_O_2_) [75].

**Figure 2:**
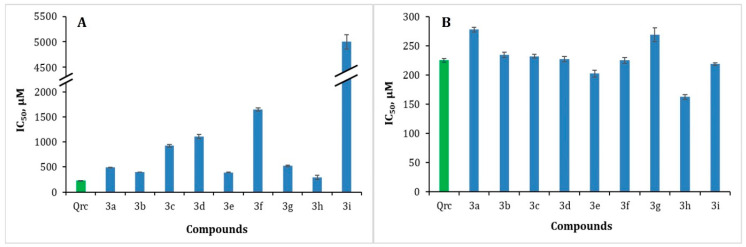
(**A**) Results of the HPSA and HRSA of the synthesized amine–valproic acid derivatives. Values are presented as IC_50_, µM. Quercetin (Qrc) was used as standard. The data are presented as mean ± SD (*n* = 3). (**A**) Hydrogen peroxide scavenging activity (HPSA); (**B**) hydroxyl radical-scavenging activity (HRSA).

In the present study, we investigated the ability of amino–valproic acid derivatives to inhibit the detrimental effects of reactive oxygen species, particularly hydrogen peroxide (H_2_O_2_). Quercetin, a well-established antioxidant, was employed as a reference standard. The results of the hydrogen peroxide scavenging activity (HPSA) assay are presented in Figure 2 and Table S1. The HPSA data revealed substantial differences in the antioxidant potential of the tested derivatives, with IC_50_ values ranging from 291 µM for compound **3h** to 5075 µM for compound **3i**.

Compound **3h**, containing two dimethoxyphenyl moieties, exhibited the strongest antioxidant potential, approaching the activity of the reference standard quercetin (IC_50_ = 225 µM). The observed activity highlights the crucial role of the electronic effects exerted by the methoxy groups (–OCH_3_). Through their strong electron-donating (+M) effect, these substituents increase the electron density of the aromatic rings, thereby facilitating the neutralization of hydrogen peroxide.

Comparative analysis revealed that the presence of two dimethoxyphenyl moieties in compound **3h** provides optimal stabilization of intermediate radical species through electron delocalization. In contrast, compounds bearing fewer or no electron-donating substituents, such as **3e** (containing two methoxy groups) and particularly **3g** (featuring two unsubstituted aromatic rings), displayed substantially higher IC_50_ values. These findings confirm that the density of alkoxy substituents is a critical determinant of antioxidant efficacy in the HPSA assay.

#### 2.3.2. Hydroxyl Radical-Scavenging Activity (HRSA)

The most potent oxidant is the hydroxyl radical (^•^OH), which is produced by ROS. It is produced by 1-electron reductions of molecular oxygen (O_2_) in cellular metabolism and is the primary cause of cytotoxicity in aerobic species, including humans [76]. Hydroxyl radicals (^•^OHs) are among the most reactive oxygen species and are capable of inducing protein carbonylation, DNA damage, and disruption of cellular membrane structures. Furthermore, they initiate the oxidation of essential polyunsaturated fatty acids, leading to lipid peroxidation, a process that plays a central role in oxidative stress-mediated cellular injury.

In the HRSA assay, the tested compounds exhibited a more homogeneous activity profile compared with the HPSA assay, with IC_50_ values ranging from 163 to 278 µM (Figure 2B). Compound **3h** once again demonstrated the highest activity, displaying an IC_50_ value of 163 µM, which surpassed that of the reference standard quercetin (232 µM). This finding suggests that the structural configuration of compound **3h** is particularly favorable for interactions with hydroxyl radicals. Although the presence of aromatic rings is a common structural feature throughout the series, the specific methoxy substituents in compound **3h** significantly enhance its radical-scavenging capacity. The lower activities observed for compounds such as **3a** and **3g**, which lack methoxy substituents, indicate that antioxidant activity is governed not merely by the number of aromatic rings but, more importantly, by their electronic characteristics as determined by the nature of the substituents. Overall, compound **3h** emerged as the most promising candidate, exhibiting an optimal structure–activity profile for the neutralization of reactive oxygen species. Its activity was comparable to, and even exceeded, that of the established antioxidant standard quercetin.

#### 2.3.3. Inhibition of Albumin Denaturation (IAD)

Anti-inflammatory therapy remains one of the cornerstones of modern pharmacology, with nonsteroidal anti-inflammatory drugs (NSAIDs) continuing to be among the most widely used therapeutic agents. Despite their high efficacy, the adverse effects associated with long-term use have prompted the search for novel derivatives with improved safety and activity profiles. In the present study, attention was focused on the synthesized derivatives whose ability to prevent serum albumin denaturation was employed as an indicator of biological activity. Albumin, the major transport protein in plasma, undergoes conformational changes under conditions of thermal stress, and inhibition of this denaturation process is widely recognized as a marker of potential anti-inflammatory and anti-rheumatic activity.

The anti-inflammatory potential of the synthesized amino–valproic acid derivatives was assessed using the inhibition of albumin denaturation (IAD) assay. The resulting dose–response curves were analyzed using four-parameter (4PL) and five-parameter (5PL) nonlinear logistic models. The calculated *Hill slope*, asymmetry parameter (***s***), and IC_50_ values provided insight into the potency, cooperativity, and overall dose–response characteristics of the tested compounds (Table 2). Ibuprofen, a clinically established nonsteroidal anti-inflammatory drug with well-documented anti-inflammatory activity, was used as the reference standard for evaluating the performance of the synthesized amino–valproic acid derivatives **3a**–**i**.

**Figure 3:**
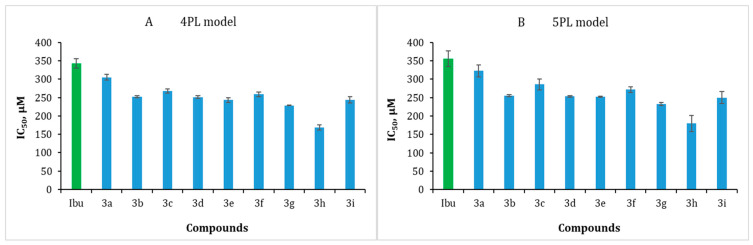
Results of the IAD of the synthesized amine–valproic acid derivatives. Values are presented as IC_50_, µM. Ibuprofen (Ibu) was used as a standard. The data are presented as mean ± SD (*n* = 3).

The IC_50_ value of ibuprofen, determined using the IAD assay, was calculated to be 343 µM according to the 4PL model and 356 µM according to the 5PL model (Table 2, Figure 3). Analysis of the results revealed that all amino–valproic acid derivatives exhibited greater anti-inflammatory activity than the reference standard ibuprofen, as evidenced by their lower IC_50_ values (Table 2, Figure 3).

Among all investigated compounds, derivative **3h** demonstrated the highest anti-inflammatory activity, displaying the lowest IC_50_ values in both the 4PL and 5PL models (Table 2, Figure 3). The superior activity of compound **3h** may be attributed to the presence of methoxy substituents on the aromatic rings at the ortho and para positions. This structure–activity relationship is consistent with observations from our previous studies, which demonstrated the favorable contribution of such substitution patterns to biological activity [77].

##### Inhibition Kinetics: Beyond Symmetrical Dose–Response Models

**Figure 4:**
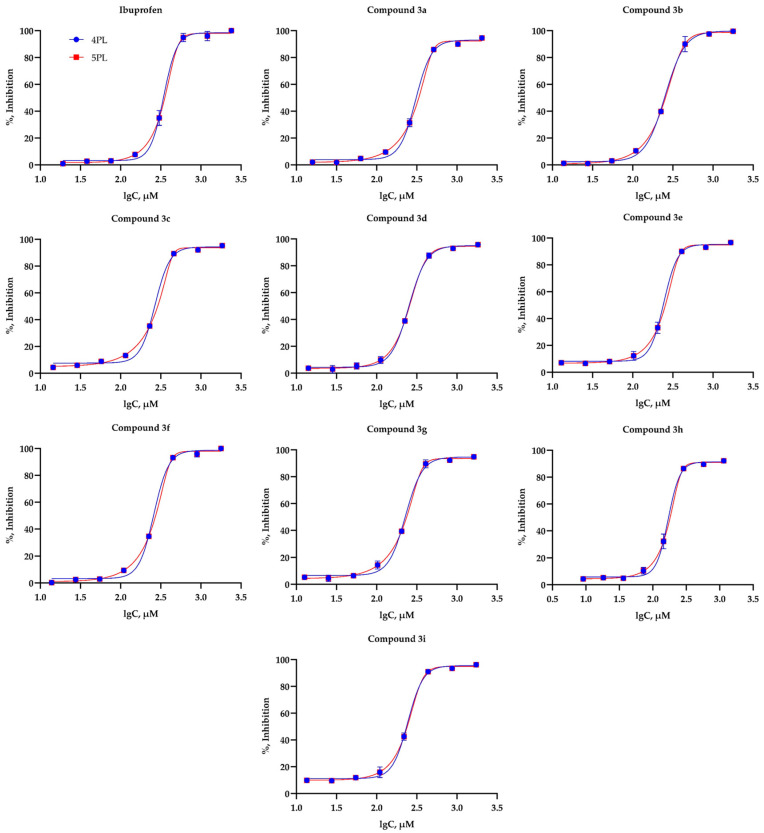
Dose–response curves of ibuprofen and synthesized amine–valproic acid derivatives (**3a**–**i**) obtained from the inhibition of albumin denaturation (IAD) assay. Experimental data values derived from four-parameter (4PL) and five-parameter (5PL) logistic models. The data are presented as mean ± SD (*n* = 3).

In contrast to many conventional pharmacological studies that employ simplified symmetrical dose–response models, the present work utilized a five-parameter logistic (5PL) model to characterize the inhibition data more accurately (Figure 4). The application of this model was motivated by the need to capture asymmetrical dose–response behavior, thereby providing a more realistic representation of the biological system under investigation. Although IC_50_ remains the primary parameter for assessing compound potency, the *Hill slope* and asymmetry parameter (***s***) provide additional mechanistic information regarding the nature of the dose–response relationship (Table 2, Figure 4). Compound **3h** displayed the lowest IC_50_ values within the series, confirming its superior anti-inflammatory potential. Nevertheless, the *Hill slope* is particularly informative because it reflects the degree of cooperativity during interactions between the compounds and the protein matrix. Accordingly, consideration of both potency and kinetic parameters enables a more comprehensive evaluation of the biological activity of the synthesized amino–valproic acid derivatives.

##### The Phenomenon of Cooperativity and Its Biological Significance

*Hill Slope*. The *Hill slope* describes the steepness of the concentration–response curve. A *Hill slope* value greater than 1.0 indicates positive cooperativity, meaning that the binding of one molecule of a derivative to the albumin matrix facilitates the subsequent binding of additional molecules. As the *Hill slope* increases, the dose–response curve becomes steeper, reflecting a more efficient and abrupt transition from low to high inhibitory activity over a narrow concentration range (Figure 4).

From a biological perspective, positive cooperativity suggests that the interaction between the compound and the protein matrix is not an independent event but rather a coordinated process in which initial binding enhances the probability of further interactions. Consequently, compounds exhibiting higher *Hill slope* values may achieve effective inhibition at lower concentration increments, indicating a more favorable pharmacodynamic profile.

The high *Hill slope* values observed for the investigated derivatives (ranging from 8 to 11) indicate that the inhibition of albumin denaturation is not merely the result of random molecular encounters but rather a complex cooperative process (Table 2, Figure 4). The elevated *Hill* coefficient suggests that the binding of a single derivative molecule to allosteric sites on albumin dramatically alters the affinity of the remaining binding sites.

Compound **3h** occupies a unique position within the studied series by combining high potency with a moderate degree of cooperativity. This specific combination of parameters indicates that the mechanism of action of compound **3h** differs from that of the derivatives exhibiting higher cooperativity (*Hill slope* 9–11). While the latter appears to rely on a cooperative “accumulation effect” to stabilize albumin, compound **3h** achieves efficient protection through a more direct and thermodynamically favorable interaction.

This distinction between affinity-driven potency (as observed for compound **3h**) and cooperative capacity (characteristic of derivatives with *Hill slope* values > 8) is fundamental to understanding how subtle changes in the chemical structure of the derivatives influence the manner in which they “communicate” with their biological targets.

##### Significance of Asymmetry (s)

The asymmetry parameter (***s***), which ranged from 0.18 to 0.57, confirms that the inhibitory responses of the investigated derivatives do not follow the classical symmetrical sigmoidal profile assumed by the 4PL model. Within the framework of the 5PL model, values of ***s*** < 1 indicate that the protective effects of the compounds are more pronounced at low-to-moderate concentrations than would be predicted by a symmetrical dose–response model. This finding suggests that the amino–valproic acid derivatives are particularly effective at preserving the native conformation of albumin during the early stages of thermal stress. While the 4PL model would effectively average out this behavior, the 5PL model captures the early protective response, thereby providing a more accurate representation of the experimental data. Consequently, the improved goodness of fit achieved by the 5PL model reflects its ability to account for biologically relevant asymmetry in the concentration–response relationship. From a mechanistic perspective, the asymmetry parameter may be regarded as an indicator of the dynamic nature of the protective response. The greater the deviation of ***s*** from unity, the more distinct the concentration-dependent behavior of the compound becomes, suggesting a more complex mode of interaction with the protein system.

#### 2.3.4. Statistical Comparison of the 4PL and 5PL Models

The anti-inflammatory activities of the nine synthesized derivatives were compared with that of the reference standard ibuprofen using nonlinear regression analysis. To accurately model the concentration–response relationships, a five-parameter logistic (5PL) model was employed. According to the Extra sum-of-squares F-test, the 5PL model provided a statistically superior fit compared with the conventional four-parameter logistic (4PL) model (*p* < 0.001) (Table S2). Statistical evaluation revealed considerable variation in F values, ranging from 5.17 to 40.38, highlighting the structure-dependent nature of the inhibition kinetics. While, for some derivatives, the transition from the symmetrical 4PL model to the asymmetrical 5PL model resulted in only moderate improvements in model performance, for others (F > 11) the use of the asymmetrical model proved essential for the accurate estimation of pharmacological parameters. These findings demonstrate that the amino–valproic acid derivatives exhibit distinct concentration–response profiles that cannot be adequately described by conventional symmetrical models. The superior performance of the 5PL model indicates the presence of biologically relevant asymmetry in the inhibitory response and underscores the importance of incorporating asymmetry when characterizing the pharmacological behavior of this class of compounds.

The statistical validity of the curve fitting was confirmed by the diagnostic parameters generated for each compound (Table S2). The obtained adjusted coefficients of determination ($R_{adj}^{2}$ > 0.99) indicate an excellent agreement between the experimental data and the mathematical model. The low residual sum of squares (RSS) values further confirm the precision of the measurements, while the low Akaike information criterion (*AIC*) values demonstrate an optimal balance between model complexity and the reliability of the estimated pharmacological parameters (Table S2).

Collectively, these statistical indicators provide strong support for the robustness of the calculated IC_50_ values and their suitability for comparing the biological activities of the investigated derivatives. The application of the 5PL logistic regression model minimized potential biases associated with the assumption of symmetrical dose–response behavior, thereby enabling a more accurate characterization and ranking of the compounds according to their biological activity.

#### 2.3.5. Evaluation of Lipophilicity (R_M_) Profiles of Synthesized Amine–Valproic Acid Derivatives

The lipophilicity of ibuprofen and a series of synthesized amine–valproic acid-based derivatives **3a**–**i** was systematically evaluated to assess their pharmacokinetic potential (Table 2). The physicochemical profile of the compounds was characterized through two complementary approaches: experimental determination of the retardation factor (***R_M_***) via reversed-phase thin-layer chromatography (RP-TLC) and computational prediction of the partition coefficient (**cLogP**) (Table 2).

The experimental ***R_M_*** values were determined to be in the range of 0.73 to 1.11. The parental compound, ibuprofen, exhibited the highest experimental lipophilicity (***R_M_*** = 1.11), serving as the baseline for the series. Among the derivatives, compound **3g** demonstrated the highest computational lipophilicity (**cLogP** = 5.411), exceeding the threshold defined by Lipinski’s Rule of Five, which may imply potential limitations in aqueous solubility. The correlation between ***R_M_*** and **cLogP** values highlights subtle variations in the molecular behavior within the chromatographic system. Notably, compound **3f** presents an interesting case, featuring the lowest calculated lipophilicity (**cLogP** = 3.562) despite a relatively high experimental ***R_M_*** value (1.01). This discrepancy suggests that specific intermolecular interactions with the stationary phase—not fully accounted for by fragment-based **cLogP** algorithms—significantly influence the hydrophobic partitioning of the substituted scaffold.

The pharmacological evaluation revealed a significant dependence of the anti-inflammatory activity (expressed as IC_50_ in the IAD assay) on the lipophilicity of the compounds. Derivatives **3h** (IC_50_ = 180 µM) and **3g** (IC_50_ = 233 µM) displayed the most potent anti-inflammatory effects, correlating with their higher experimental ***R_M_*** values (Table 2). In contrast, the parent compound, ibuprofen, exhibited lower inhibitory capacity, suggesting that the introduced chemical modifications significantly enhance the affinity towards the target protein. These results highlight a positive correlation between the degree of lipophilicity and the inhibitory efficiency against albumin denaturation, positioning compounds **3g** and **3h** as the most promising candidates for further pharmacological development.

### 2.4. In Silico Analysis

#### 2.4.1. DFT Calculations

The DFT calculations performed for compounds **3a**–**i** revealed significant variations in the frontier molecular orbital energies depending on both the substituent pattern and the solvent environment (Table 3). In vacuum, the HOMO energies ranged from −8.77 eV for compound **3b** (chlorine substituted benzene) to −7.44 eV for compound **3f** (dimethoxy substituted benzene), indicating that compound **3b** possesses the highest electronic stability and the lowest tendency to donate electrons. The highest LUMO energies were computed for compounds **3e** and **3f** at 2.04–2.06 eV (both compounds are dimethoxy-substituted benzenes), suggesting a lower electron affinity compared to the other derivatives. In contrast, compounds **3b** (chlorine-substituted benzene) and **3g** (two unsubstituted benzene rings) exhibited lower LUMO energy values, which may indicate an enhanced ability to participate in electron-accepting processes.

The solvent effect on the electronic parameters was relatively moderate; however, a slight stabilization of the frontier orbitals was observed with increasing solvent polarity. For most compounds, the transition from vacuum to polar and aqueous media produced a small shift in the HOMO levels toward less negative values accompanied by a slight increase in the LUMO energies. This behavior suggests electrostatic stabilization of the electronic distribution in media with higher dielectric constants. Nevertheless, compounds **3f** (dimethoxy-substituted benzene) and **3h** (two dimethoxy-substituted benzenes) displayed a different trend, characterized by a decrease in both HOMO and LUMO energies in polar solvents, which may reflect stronger solvent–solute interactions associated with localized electron density on specific structural fragments. The depiction of the HOMO and LUMO is presented in Figures S21–S29 (Supplementary Material).

Overall, the DFT results indicate that the electronic properties of the **3a**–**i** compounds are governed by both intrinsic structural factors and solvent effects. The relatively small differences observed between nonpolar, polar, and aqueous environments suggest good electronic stability of the investigated compounds. Furthermore, the variations in HOMO and LUMO energy levels may significantly influence the chemical reactivity, charge-transfer characteristics, and potential interactions of these compounds with biological targets.

The calculated molecular descriptors of compounds **3a**–**i** revealed important differences in their physicochemical properties, which may significantly influence their biological activity and pharmacokinetic behavior (Table 4). The lipophilicity values (LogP) ranged from 3.65 for compound **3f** (dimethoxy-substituted benzene) to 5.77 for compound **3g** (two unsubstituted benzenes), indicating an overall moderate-to-high hydrophobic character across the series, suggesting enhanced membrane permeability and strong hydrophobic interactions. On the other hand, compounds **3e** and **3f** (dimethoxy-substituted benzenes) displayed comparatively lower lipophilicity, which may favor improved aqueous solubility. The molecular surface area and volume also increased progressively for several derivatives, with compound **3h** (two dimethoxy-substituted benzenes) presenting the largest molecular dimensions (area = 507.08 Å^2^; volume = 486.62 Å^3^), due to the presence of bulkier structural fragments.

The polar surface area (PSA) values varied between 23.918 and 49.402 Å^2^, indicating moderate polarity for the investigated compounds. Lower PSA values were found for compounds **3a** (unsubstituted benzene), **3b** (chlorine-substituted benzene), and **3g** (two benzenes) due to their hydrophobic structural features, while higher PSA values for compounds **3h** (two dimethoxy-substituted benzenes) and **3i** (indole) indicate a greater contribution of polar functionalities capable of participating in intermolecular interactions such as hydrogen bonding. Since PSA is closely associated with membrane transport properties, the obtained values suggest that most compounds maintain a favorable balance between lipophilicity and polarity, which could support adequate permeability across biological membranes. The depiction of the electrostatic-potential maps of the compounds is presented in Figures S30–S38 (Supplementary Material).

The dipole moment values ranged from 1.95 to 4.16 D, demonstrating differences in charge distribution within the molecules. Compound **3i** (indole) exhibited the highest dipole moment, indicating a more pronounced electronic asymmetry and potentially stronger interactions with polar environments or biological targets. In contrast, compound **3b** (chlorine-substituted benzene) showed the lowest dipole moment, suggesting a more uniform electronic distribution. Taken together, these molecular descriptors indicate that structural modifications within the **3a**–**i** series substantially affect both steric and electronic properties, which may ultimately influence their reactivity, intermolecular interactions, and potential biological activity.

#### 2.4.2. Molecular Docking

The best binding affinities of the compounds **3a**–**i** to the four studied albumin sites, obtained through molecular docking with AutoDock Vina, are summarized in Table 5, while the AutoDock results are presented in Table 6.

Human serum albumin (HSA) was selected as the molecular docking target in accordance with the experimental IAD assay, in which albumin was used as the model protein. The HSA crystal structure PDB ID: 7JWN was employed because it contains ketoprofen bound at defined HSA binding sites, providing a structurally relevant model for investigating the interactions of the newly synthesized compounds with albumin. This approach allows the in silico results to complement the experimental IAD data and provides molecular-level insight into the observed effects.

The docking results obtained with AutoDock Vina revealed notable differences in the predicted binding affinities of the investigated compounds **3a**–**i** toward the four albumin binding regions. Overall, site 3 exhibited the most favorable binding energies for nearly all compounds, suggesting that this pocket provides the highest degree of structural and physicochemical complementarity for the studied ligands. In contrast, the cleft region and Sudlow site 1 generally displayed weaker interactions. Among the investigated compounds, **3g**–**i** (diphenyl or indole derivatives) exhibited the strongest predicted affinities across all binding sites. In particular, **3g** showed the lowest binding energy at site 3 (−9.9 kcal/mol), indicating a highly stable ligand–protein complex. Compounds **3h** and **3i** also displayed strong interactions with site 3, with binding energies of −9.2 kcal/mol. These findings suggest that structural features present in the respective molecules, such as two phenyl rings of indole, significantly enhance albumin binding.

Sudlow site 2 also appeared to be a favorable interaction region, especially for compounds **3g**–**3i**, which exhibited binding energies below −8.0 kcal/mol. The relatively consistent affinity trend observed across the four sites indicates that the compounds possess a broad capability for interaction with albumin, although with clear preference for site 3.

The results given by AutoDock were generally consistent with those obtained using AutoDock vina, confirming the reproducibility of the predicted affinities.

As observed previously, site 3 was identified as the most favorable binding region for almost all investigated compounds, while the cleft region and Sudlow site 1 displayed comparatively weaker interactions. Site 3 was selected as the binding region for subsequent molecular dynamics simulations and MM-PBSA calculations, as it consistently yielded the most favorable binding affinities for all investigated compounds across both docking programs employed, providing cross-validated evidence for its preferential interaction with the studied **3a**–**i** compounds.

Compounds **3g**, **3h**, and **3i** again emerged as the best binding compounds, having the best docking energies across multiple binding sites. Compound **3g** exhibited the strongest predicted interaction at site 3 (ΔG = −9.84 kcal/mol), followed closely by compound **3h**. The consistently high affinities predicted for compounds **3g**–**i** suggest that these derivatives possess structural complementarity with the albumin binding pockets.

**Figure 5:**
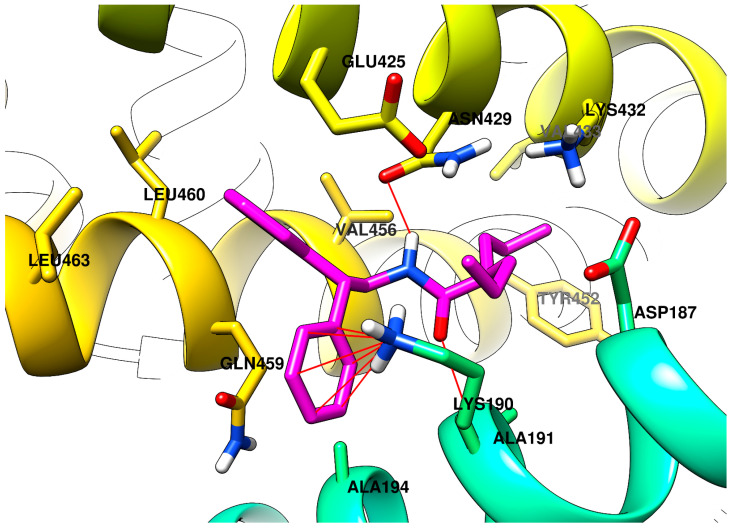
The top-ranked conformation of compound **3g** (carbon atoms depicted in magenta) in the cleft site of albumin (PDB: 7JWN). The amide oxygen atom interacts as a hydrogen-bond acceptor with the Lys190-Ala191 peptide bridge, while one of the benzenes interacts with the positively charged sidechain of Lys190 in a π-cation interaction. The other benzene fits well in a hydrophobic region surrounded by the sidechains of Leu460, Leu463 and Val456.

**Figure 6:**
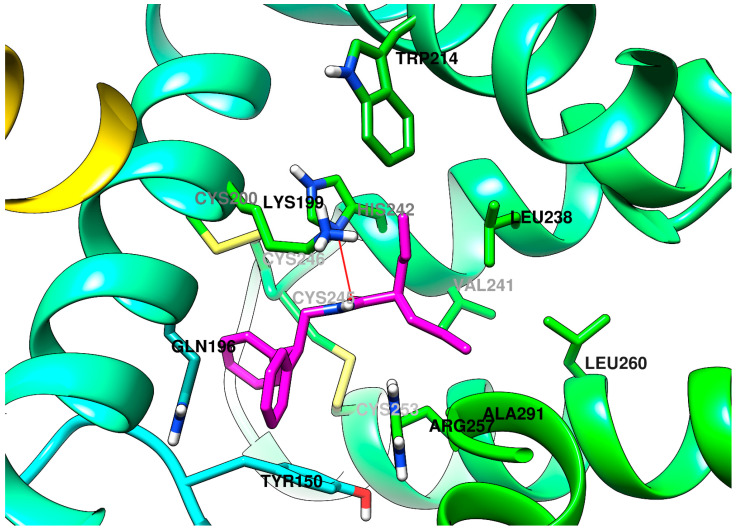
The top-ranked conformation of compound **3g** (carbon atoms depicted in magenta) bound in the Sudlow 1 site of albumin (PDB: 7JWN). The amide oxygen atom interacts with the sidechain of His242, while the flexible chain comprised by the sp^3^ carbon atoms from VPA is found in a hydrophobic environment comprised by the sidechains of Leu260, Leu238, Ala291, Ala241, and Trp214.

**Figure 7:**
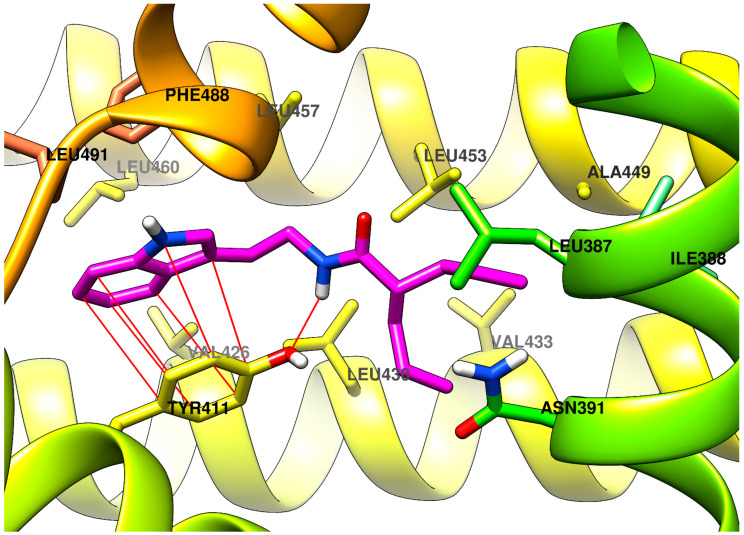
The top-ranked conformation of compound **3i** (carbon atoms depicted in magenta) bound in the Sudlow 2 site of albumin (PDB: 7JWN). The amide nitrogen of compound **3i** is involved in a hydrogen bond as donor to the phenol oxygen of Tyr411, while the indole of compound **3i** is predicted to interact with the benzene ring of Tyr411 in a π-π stacking interaction. The flexible chain comprised by the sp^3^ carbon atoms from VPA is found in a hydrophobic environment comprised by the sidechains of Leu430, Val433, Leu387, Ile388, Ala449 and Leu453.

**Figure 8:**
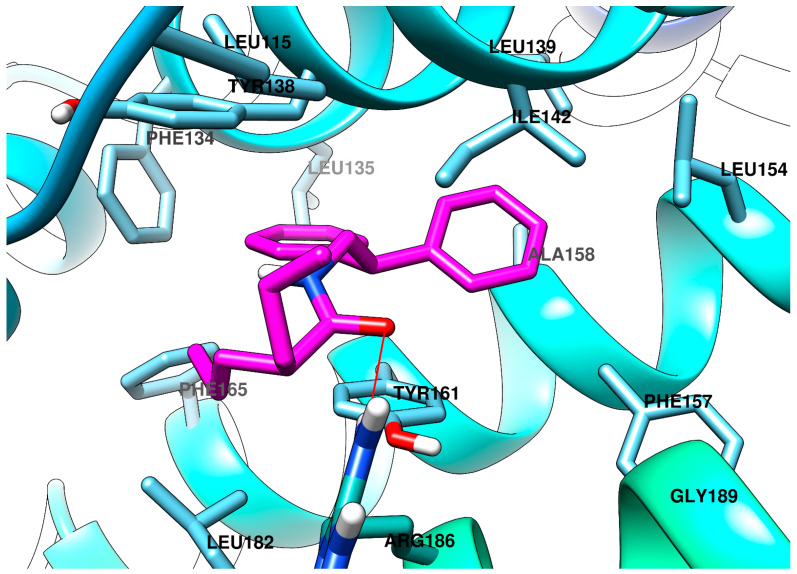
The top-ranked conformation of compound **3g** (carbon atoms depicted in magenta), bound in the site 3 of albumin (PDB: 7JWN). The amide oxygen is predicted to interact with the positively charged sidechain of Arg186, while the benzene rings and the flexible chain comprised by the sp^3^ carbon atoms from VPA are found in a hydrophobic environment comprised by the sidechains of Phe157, Leu154, Ala158, Ile142, Leu139, Tyr138, Leu115, Phe134, Leu135 and Phe165.

The conformation of the best binding compound in each of the four studied sites of albumin as given by AutoDock vina is depicted in Figure 5, Figure 6, Figure 7 and Figure 8.

#### 2.4.3. Molecular Dynamics

The stability of albumin-**3a**–**i** chimeric complexes created using the top binding conformation of the compounds **3a**–**i** in site 3 of HSA was assessed through molecular dynamics simulations. To investigate the structural stability of the complexes during molecular dynamics simulations, several parameters were analyzed, namely ligand heavy-atom RMSD, HSA backbone RMSD, HSA radius of gyration (Rg), and ligand–HSA hydrogen-bond interactions recorded along the trajectories. The summary of the resulting data after analysis of the molecular dynamics trajectories is presented in Table 7 and plotted in Figures S39–S47 (Supplementary Materials).

The ligands’ RMSDs ranged from 0.27 to 0.63 nm, suggesting different degrees of conformational adaptation of the ligands inside site 3 of HSA. The lowest deviation was observed for **3f** (0.27 nm), indicating a highly stable binding mode and limited ligand rearrangement during the simulation. Similarly, ligands **3e** and **3h** (0.39 nm both) showed relatively stable conformations. In contrast, **3c** (0.63 nm) and **3b** (0.57 nm) exhibited higher fluctuations, which indicate repositioning of the two ligands during the molecular dynamics simulation. It is worth mentioning the studied compounds have a high degree of intrinsic flexibility due to the flexible aliphatic sp^3^ carbon atoms connecting the nitrogen amide with the (hetero)arene and the heptan-4-il moiety from valproic acid. Thus, a higher degree of movement of the heavy atoms of the ligands could be expected. The visual inspection of the ligands’ position in the case of complexes of the compounds **3b**, **3c**, **3d** and **3i** indicated they had no drift outside of the binding pocket, just a reorientation in the binding site. Their molecular dynamics simulations were extended to 150 ns, to confirm their complexes’ stability in time.

The HSA backbone RMSD values varied between 0.23 and 0.41 nm for the ligand-bound systems, while the apo HSA (without ligand bound) had a backbone RMSD of 0.38 nm. The comparable values between the complexes and the free protein suggest that ligand binding did not induce major conformational changes in the HSA structure. The lowest protein backbone deviation was observed for **3a** (0.23 nm), followed by **3e** (0.24 nm) and **3g** (0.25 nm), indicating a stabilizing effect of these ligands on the protein conformation.

The radius of gyration values remained relatively constant across all simulations (2.73–2.78 nm), comparable to free HSA. This narrow variation indicates that the overall compactness and global structural organization of HSA were preserved during the simulations, with no significant unfolding being detected.

Hydrogen-bond analysis revealed differences in this type of ligand–HSA interaction. The highest number of hydrogen bonds was found for **3h** (1.80 bonds/ns), followed by **3e** (1.25 bonds/ns), suggesting a more persistent interaction with HSA. Ligands **3a**, **3c**, **3g**, and **3i** also maintained relatively favorable hydrogen-bonding interactions (0.84–0.89 bonds/ns). Interestingly, **3f** (0.06 bonds/ns) and **3b** (0.13 bonds/ns) showed limited hydrogen bonding despite their relatively low RMSD values, indicating that their stability may be primarily driven by hydrophobic interactions or other types of non-covalent contacts. For compound **3d**, the conformational reorientation inside the binding pocket observed in the latter part of the simulation was accompanied by a drastic increase in the average number of hydrogen bonds, indicating drift toward a more favorable position than the one initially predicted by molecular docking. A similar behavior was observed for **3g**, which immediately in the first 10 ns of the simulation changed its pose and started interacting with HSA via a hydrogen bond, which was not initially predicted in the molecular docking.

Considering all evaluated parameters, **3e** and **3h** appeared to form the most stable complexes, combining low ligand RMSD values with hydrogen-bonding interaction and minimal effects on HSA conformation. The complex of **3f** also demonstrated good positional stability; however, the reduced hydrogen-bonding frequency suggests a different binding stabilization mechanism than **3e** and **3h**. Overall, the molecular dynamics results support the formation of stable HSA–ligand complexes for the investigated compounds.

Among the evaluated complexes, **3g** and **3i** also exhibited favorable stability profiles during the molecular dynamics simulations, but with a higher movement from the initial position. The **3g**–HSA complex showed a relatively low ligand RMSD value (0.46 nm) together with a low protein backbone deviation (0.25 nm), indicating that ligand accommodation within the binding site occurred without inducing significant structural rearrangements of the protein. Moreover, the relatively high hydrogen-bond frequency (0.87 bonds/ns) suggests that the complex was stabilized by persistent polar interactions throughout the simulation. The constant radius of gyration (2.76 nm) further supports the unaffected preservation of the global HSA structure. Similarly, the **3i**–HSA complex displayed a moderate ligand RMSD (0.53 nm) and protein backbone RMSD (0.36 nm), suggesting a stable but somewhat more flexible binding arrangement compared with **3g**. Despite slightly higher conformational fluctuations, the hydrogen-bond frequency (0.84 bonds/ns) indicates that the ligand maintained relevant interactions with HSA during the trajectory. The radius of gyration value (2.74 nm) remained comparable to the other complexes, confirming that ligand binding did not affect the overall compactness of the protein. When considering all parameters together, **3g** and **3i** represent complexes with balanced dynamic behavior, maintaining stable ligand positioning in time while preserving the structure of HSA. Although their ligand RMSD values were not the lowest observed, the combination of moderate structural fluctuations and sustained hydrogen-bond interactions suggests a favorable binding mode.

#### 2.4.4. Molecular Mechanics—Poisson–Boltzmann Surface Area

To obtain detailed information about the interaction between ligands **3a**–**i** with HSA, the binding free energies of ligands **3a**–**i** to HSA were calculated and are presented in Table 8.

The MM-PBSA analysis revealed favorable binding free energies for all ligands **3a**–**i**, with ΔG ranging from −28.29 to −43.64 kcal/mol, indicating stable interactions with site 3 of HSA. The main contribution to ligand binding was provided by van der Waals interactions, which showed consistently favorable energetic contributions across all complexes, ranging from −32.77 kcal/mol for ligand **3a** to −51.91 kcal/mol for ligand **3h**. Electrostatic interactions also contributed to complex stabilization, although their magnitude varied between ligands; the strongest electrostatic contribution was observed for ligand **3e** (−20.52 kcal/mol), while ligand **3b**, the only halogenated compound in the present series, had an unfavorable electrostatic interaction profile (5.48 kcal/mol). The solvation energy contributions were unfavorable for all ligand–HSA complexes, with positive values ranging from 2.41 kcal/mol (compound **3b**) to 28.02 kcal/mol (compound **3h**), indicating that polar solvation effects partially oppose complex stabilization, although their impact is compensated by other favorable interactions. These results suggest that the enhanced affinity of these ligands is primarily driven by strong hydrophobic and van der Waals interactions within the binding pocket, supported by favorable electrostatic contributions for some compounds.

The contribution of decomposition to the interaction of the surrounding amino acids found in 5 Å near the ligands **3a**–**i** in site 3 of HSA is presented in Table 9, while the graphical depiction of the data is presented in Figures S48–S56 (Supplementary Material).

The MM-PBSA energy decomposition analysis revealed that the stabilization of ligands **3a**–**i** is mainly mediated by favorable interactions with aromatic and hydrophobic residues. TYR138 and ILE142 displayed the most favorable contributions, suggesting their important role in ligand accommodation within the binding pocket. The contribution of TYR138 came more from aromatic π interactions and hydrophobic contacts than from the interactions of the hydroxyl group. Other residues, including TYR161, PHE157, LEU182, and several leucine residues, also contributed favorably to complex stabilization, particularly for compounds **3g**, **3h**, and **3f**. Overall, the binding of compounds **3a**–**i** to HSA site 3 appears to be driven mainly by aromatic, van der Waals and hydrophobic contacts.

In opposition to stabilizing contacts, several residues exhibited unfavorable contributions for specific ligands, indicating the presence of transiently repulsive interactions. Notably, positively charged residues occasionally displayed positive energy contributions: **3d**, **3e** and **3i** (all three have a *p*-methoxy substituent and an ethylene linker) with ARG117 and **3g** and **3h** (both are diphenyl methane derivatives) with ARG145, suggesting electrostatic repulsion. These unfavorable interactions appear to be ligand structure-dependent, but they are outweighed by the dominant favorable hydrophobic and aromatic interactions.

## 3. Materials and Methods

All reagents and chemicals were purchased from commercial suppliers (Sigma-Aldrich S.A. and Riedel-de Haën, Sofia, Bulgaria) and were used without further purification. Mechanosynthesis was conducted using a Retsch Planetary Ball Mill PM 200 (University of Plovdiv; RETSCH, Haan, Germany) equipped with a 25 mL stainless steel grinding jar. The grinding media consisted of 28 g of 12 mm hardened-steel grinding balls. Nuclear magnetic resonance (NMR) spectra were recorded on a Bruker Avance II+600 spectrometer (BAS-IOCCP-Sofia, Bruker, Billerica, MA, USA), operating at 600 MHz for ^1^H and 150.9 MHz for ^13^C. Spectra were acquired in DMSO-*d*_6_, with chemical shifts (δ) referenced to tetramethylsilane (TMS, δ = 0.00 ppm) and coupling constants (*Js*) reported in hertz (Hz). All NMR measurements were performed at ambient temperature (approximately 295 K). Melting points were determined using a Boetius hot-stage apparatus (University of Plovdiv; Boetius, Bremen, Germany) and are reported uncorrected. Infrared (IR) spectra were obtained on a Bruker Alpha II FT-IR spectrometer (University of Plovdiv; Bruker, Billerica, MA, USA). High-resolution mass spectrometry (HRMS) analyses were conducted using a Q Exactive Plus mass spectrometer equipped with a heated electrospray ionization (HESI-II) source (Thermo Fisher Scientific, Bremen, Germany) and coupled to a Dionex Ultimate 3000RSLC UHPLC system (Thermo Fisher Scientific, Waltham, MA, USA). Thin-layer chromatography (TLC) was carried out on 0.2 mm silica gel 60 plates (Fluka, Merck KGaA, Darmstadt, Germany).

### 3.1. Synthetic Procedures

#### 3.1.1. Obtaining 2-Propylpentanoyl Chloride

Valproic acid (1.0 mmol, 0.1442 g) was dissolved in toluene (20 mL), and an excess of thionyl chloride (1.2 mmol, 0.087 mL) was added. The reaction mixture was refluxed for 2 h to convert the carboxylic acid into the corresponding acid chloride. After completion of the reaction, the volatile components and excess toluene were removed under reduced pressure using a rotary evaporator. The resulting crude residue was dissolved in a minimal volume of dichloromethane (5 mL) and used directly in the subsequent reaction without further purification.

#### 3.1.2. Synthesis of Amides **3a**–**i**

The corresponding amine (1 mmol), 2-propylpentanoyl chloride (1 mmol, 0.1626 g), and Et_3_N (0.168 mL, 1.2 mmol) were placed in a 25 mL stainless steel grinding jar, along with four 12 mm hardened-steel grinding balls. Mechanochemical reaction was performed in a Retsch PM 200 planetary ball mill with constant rotation (500 rpm) for 1 min. Progress of the reaction was monitored by thin-layer chromatography, confirming complete consumption of the amine starting material. Subsequent to that, the milling jar contents underwent a double wash with dichloromethane (10 mL) and water (5 mL) each, leading to the separation of the two layers. The aqueous layer was then subjected to extraction with dichloromethane (2 × 10 mL), and the combined organic fractions were washed with dilute aqueous hydrochloric acid (HCl:H_2_O = 1:4), saturated aqueous sodium carbonate (Na_2_CO_3_), and brine. The organic phase was separated, dried over anhydrous sodium sulfate, and concentrated under reduced pressure. The crude product was purified by short-column chromatography on neutral aluminum oxide (Al_2_O_3_) using CH_2_Cl_2_ as eluent to afford the hybrid molecules. Melting points were determined on the purified samples without further recrystallization. All compounds were fully characterized by ^1^H and ^13^C NMR and IR as well as HRMS spectra. 

##### *N*-Phenethyl-2-propylpentanamide **3a**

White solid, (m.p. 97–99 °C, crystalized in petroleum/diethyl ether = 1/1 *v/v*), yield 97% (0.240 g), *R_f_* = 0.46 (petroleum/diethyl ether = 1/1 *v*/*v*), ^1^H NMR (600 MHz, DMSO-*d*_6_) δ 7.86 (t, *J* = 5.6 Hz, 1H), 7.31–7.25 (m, 2H), 7.23–7.16 (m, 3H), 3.29 (q, *J* = 7.1 Hz, 2H), 2.71 (t, *J* = 7.2 Hz, 2H), 2.13–2.06 (m, 1H), 1.46–1.37 (m, 2H), 1.23–1.17 (m, 2H), 1.15–1.09 (m, 4H), 0.81 (t, *J* = 7.2 Hz, 6H). ^13^C NMR (151 MHz, DMSO-*d*_6_) δ 175.2 (C=O), 140.0 (Ar), 129.1 (Ar), 128.7 (Ar), 126.4 (Ar), 45.7 (CH), 40.3 (CH_2_-NH), 35.7 (C_sp_^2^-CH_2_), 35.3 (2× CHCH_2_), 20.6 (2× CH_2_CH_2_CH_3_), 14.5 (2× CH_3_). HRMS Electrospray ionization (ESI) *m*/*z* calcd for [M + H]^+^ C_16_H_26_NO^+^ = 248.2009, found 248.2001 (mass error ∆m = −3.22 ppm); for [M + Na]^+^ C_16_H_25_NONa^+^ = 270.1828, found 270.1819 (mass error ∆m = −3.33 ppm). IR (KBr) ν_max_., cm^−1^: 3288 *ν* (N-H), 2953 *ν_as_* (CH_3_), 2929 *ν_as_* (CH_2_), 2871 *ν_s_* (CH_3_), 1635 *ν* (C=O), 1454 *δ_as_* (CH_3_), 1381 *δ_s_* (CH_3_).

##### *N*-(3-Chlorophenethyl)-2-propylpentanamide **3b**

White solid (m.p. 89–91 °C, crystalized in petroleum/diethyl ether = 1/1 *v/v*), yield 93% (0.261 g), *R_f_* = 0.52 (petroleum/diethyl ether = 1/1 *v*/*v*), ^1^H NMR (600 MHz, DMSO-*d*_6_) δ 7.83 (t, *J* = 5.6 Hz, 1H), 7.30 (t, *J* = 7.8 Hz, 1H), 7.28–7.23 (m, 2H), 7.17 (dt, *J* = 7.5, 1.4 Hz, 1H), 3.32 (q, *J* = 6.9, 5.5 Hz, 2H), 2.73 (t, *J* = 6.9 Hz, 2H), 2.07 (tt, *J* = 9.6, 4.7 Hz, 1H), 1.44–1.34 (m, 2H), 1.22–1.14 (m, 2H), 1.14–1.06 (m, 4H), 0.80 (t, *J* = 7.3 Hz, 6H). ^13^C NMR (151 MHz, DMSO-*d*_6_) δ 175.2 (C=O), 142.7 (Ar), 133.3 (Ar), 130.4 (Ar), 129.1 (Ar), 128.0 (Ar), 126.4 (Ar), 45.7 (CH), 39.9 (NHCH_2_), 35.3 (CH_3_CH_2_CH_2_CHCH_2_CH_2_CH_3_), 35.1 (CH_2_CH_2_NH), 20.6 (CH_3_CH_2_CH_2_CHCH_2_CH_2_CH_3_), 14.5 (CH_3_CH_2_CH_2_CHCH_2_CHCH_3_). HRMS electrospray ionization (ESI) *m*/*z* calcd for [M + Na]^+^ C_16_H_24_ClNONa^+^ = 304.1439, found 304.1431 (mass error ∆m = −2.63 ppm). IR (KBr) ν_max_., cm^−1^: 3287 *ν* (N-H), 2959 *ν_as_* (CH_3_), 2930 *ν_as_* (CH_2_), 2873 *ν_s_* (CH_3_), 1636 *ν* (C=O), 1461 *δ_as_* (CH_3_), 1383 *δ_s_* (CH_3_).

##### *N*-(3-Methoxyphenethyl)-2-propylpentanamide **3c**

The compound was synthesized in our previous study [78].

White solid (m.p. 88–90 °C, crystalized in petroleum/diethyl ether = 1/1 *v/v*), yield 96% (0.266 g), *R_f_* = 0.45 (petroleum ether/diethyl ether = 1/1 *v*/*v*), ^1^H NMR (600 MHz, DMSO-*d*_6_) δ 7.85 (t, *J* = 5.3 Hz, 1H), 7.19 (t, *J* = 7.9 Hz, 1H), 6.80–6.73 (m, 3H), 3.73 (s, 3H), 3.29 (q, 2H), 2.68 (t, *J* = 7.2 Hz, 2H), 2.13–2.05 (m, 1H), 1.41 (ddt, *J* = 12.4, 9.3, 6.8 Hz, 2H), 1.23–1.17 (m, 2H), 1.16–1.09 (m, 4H), 0.81 (t, *J* = 7.3 Hz, 6H). ^13^C NMR (151 MHz, DMSO-*d*_6_) δ 175.2 (C=O), 159.7 (Ar), 141.6 (Ar), 129.6 (Ar), 121.4 (Ar), 114.7 (Ar), 112.0 (Ar), 55.3 (OCH_3_), 45.7 (CH), 40.2 (CH_2_CH_2_NH), 35.8 (ArCH_2_CH_2_NH), 35.4 (2× CHCH_2_CH_2_CH_3_), 20.6 (2× CH_2_CH_3_), 14.5 (2× CH_3_). HRMS electrospray ionization (ESI) *m*/*z* calcd for [M + Na]^+^ C_17_H_27_NNaO_2_^+^ = 300.1934 found 300.1924 (mass error ∆m = −3.33 ppm), IR (KBr) ν_max_., cm^−1^: 3289, 3085 *ν* (N-H), 1626 *ν* (C=O), 2832 *ν_s_* (O–CH_3_), 1310, 1050 *δ* (C_sp_^3^–O–C_sp_^2^).

##### *N*-(4-Methoxyphenethyl)-2-propylpentanamide **3d**

The compound was synthesized in our previous study [79].

White solid (m.p. 103–104 °C, crystalized in petroleum/diethyl ether = 1/1 *v/v*), yield 97% (0.2701 g), *R_f_* = 0.51 (petroleum/diethyl ether = 1/1 *v*/*v*), ^1^H NMR (600 MHz, DMSO-*d*_6_) δ 7.75 (t, *J* = 5.7 Hz, 1H), 7.04 (d, *J* = 8.9 Hz, 2H), 6.77 (d, *J* = 8.9 Hz, 2H), 3.64 (s, 3H), 3.18 (q, *J* = 12.8, 7.3 Hz, 2H), 2.56 (t, *J* = 7.3 Hz, 2H), 2.05–1.98 (m, 1H), 1.40–1.28 (m, 2H), 1.15–1.09 (m, 2H), 1.08–1.02 (m, 4H), 0.74 (t, *J* = 7.2 Hz, 6H). ^13^C NMR (151 MHz, DMSO-*d*_6_) δ 175.1 (C=O), 158.1 (Ar), 131.9 (Ar), 130.0 (Ar), 114.1 (Ar), 55.4 (CH_3_O), 45.7 (CH_2_CH(C=O)CH_2_), 40.6 (CH_2_NH), 35.4 (CH_2_CH_2_NH), 34.9 (CHCH_2_CH_2_CH_3_), 20.6 (CHCH_2_CH_2_CH_3_), 14.5 (CHCH_2_CH_2_CH_3_). HRMS electrospray ionization (ESI) *m*/*z* calcd for [M + Na]^+^ C_17_H_27_NNaO_2_^+^ = 300.1934, found 300.1925 (mass error ∆m = −3.00 ppm). IR (KBr) ν_max_., cm^−1^: 3293 *ν* (N-H), 2954 *ν_as_* (CH_3_), 2930 *ν_as_* (CH_2_), 2869 *ν_s_* (CH_3_), 1641, 1614 *ν* (C=O), 1457 *δ_as_* (CH_3_), 1380 *δ_s_* (CH_3_).

##### *N*-(3,4-Dimethoxyphenethyl)-2-propylpentanamide **3e**

The compound was synthesized in our previous study [80].

White solid (m.p. 108–109 °C, crystalized in petroleum/diethyl ether = 1/3), yield 93% (0.2859 g), *R_f_* = 0.47 (petroleum/diethyl ether = 1/3 *v*/*v*), ^1^H NMR (600 MHz, DMSO-*d*_6_) δ 7.74 (t, *J* = 5.7 Hz, 1H), 6.77 (d, *J* = 8.1 Hz, 1H), 6.73 (d, *J* = 2.0 Hz, 1H), 6.63 (dd, *J* = 8.2, 2.1 Hz, 1H), 3.67 (s, 3H), 3.64 (s, 3H), 3.20 (q, *J* = 7.1, 5.8 Hz, 2H), 2.56 (t, *J* = 7.2 Hz, 2H), 2.05–1.98 (m, 1H), 1.37–1.30 (m, 2H), 1.16–1.10 (m, 2H), 1.09–1.02 (m, 4H), 0.74 (t, *J* = 7.2 Hz, 6H). ^13^C NMR (151 MHz, DMSO-*d*_6_) δ 175.6 (C=O), 149.1 (Ar), 147.7 (Ar), 132.5 (Ar), 120.9 (Ar), 113.0 (Ar), 112.3 (Ar), 56.0 (OCH_3_), 55.8 (OCH_3_), 45.7 (CH), 40.5 (CH_2_NH), 35.4 (CH_2_CH_2_NH), 35.3 (2× CHCH_2_CH_2_CH_3_), 20.7 (2× CH_2_CH_2_CH_3_), 14.5 (2× CH_3_). HRMS electrospray ionization (ESI) *m*/*z* calcd for [M + Na]^+^ C_18_H_29_NNaO_3_^+^ = 330.2040, found 330.2031 (mass error ∆m = −2.73 ppm). IR (KBr) ν_max_., cm^−1^: 3289 *ν* (N-H), 2955 *ν_as_* (CH_3_), 2930 *ν_as_* (CH_2_), 2871 *ν_s_* (CH_3_), 1640 *ν* (C=O), 1452 *δ_as_* (CH_3_), 1389 *δ_s_* (CH_3_).

##### *N*-(3,4-Dimethoxybenzyl)-2-propylpentanamide **3f**

White solid (m.p. 135–136 °C, crystalized in petroleum/diethyl ether = 1/2 *v/v*), yield 97% (0.285 g), *R_f_* = 0.44 (petroleum/diethyl ether = 1/2 *v*/*v*), ^1^H NMR (600 MHz, DMSO-*d*_6_) δ 8.28 (t, *J* = 6.0 Hz, 1H), 6.88 (d, *J* = 8.2 Hz, 1H), 6.84 (d, *J* = 2.0 Hz, 1H), 6.76 (dd, *J* = 8.2, 2.1 Hz, 1H), 4.21 (d, *J* = 6.0 Hz, 2H), 3.73 (s, 3H), 3.72 (s, 3H), 2.26–2.18 (m, 1H), 1.53–1.44 (m, 2H), 1.29–1.24 (m, 2H), 1.24–1.18 (m, 4H), 0.85 (t, *J* = 7.2 Hz, 6H). ^13^C NMR (151 MHz, DMSO-*d*_6_) δ 175.2 (C=O), 149.1 (Ar), 148.1 (Ar), 132.9 (Ar), 119.6 (Ar), 112.1 (Ar), 111.4 (Ar), 56.0 (OCH_3_), 55.7 (OCH_3_), 45.7 (CH_2_NH), 42.0 (CH), 35.4 (CH_3_CH_2_CH_2_CHCH_2_CH_2_CH_3_), 20.7 (CH_3_CH_2_CH_2_CHCH_2_CH_2_CH_3_), 14.5 (CH_3_CH_2_CH_2_CHCH_2_CH_2_CH_3_). HRMS electrospray ionization (ESI) *m*/*z* calcd for [M + Na]^+^ C_17_H_27_NO_3_Na^+^ = 316.1883, found 316.1874 (mass error ∆m = −2.85 ppm). IR (KBr) ν_max_., cm^−1^: 3285 *ν* (N-H), 2951 *ν_as_* (CH_3_), 2925 *ν_as_* (CH_2_), 2872 *ν_s_* (CH_3_), 1643 *ν* (C=O), 1454 *δ_as_* (CH_3_), 1388 *δ_s_* (CH_3_).

##### *N*-(2,2-Diphenylethyl)-2-propylpentanamide **3g**

White solid (m.p. 94–95 °C, crystalized in petroleum/diethyl ether = 1/1 *v/v*), yield 97% (0.313 g), *R_f_* = 0.68 (petroleum/diethyl ether = 1/1 *v*/*v*), ^1^H NMR (600 MHz, DMSO-*d*_6_) δ 7.83 (t, *J* = 5.7 Hz, 1H), 7.31–7.25 (m, 8H), 7.21–7.15 (m, 2H), 4.24 (t, *J* = 8.0 Hz, 1H), 3.71 (dd, *J* = 8.1, 5.6 Hz, 2H), 2.04–1.99 (m, 1H), 1.37–1.29 (m, 2H), 1.15–1.07 (m, 2H), 1.02–0.94 (m, 4H), 0.71 (t, *J* = 7.3 Hz, 6H). ^13^C NMR (151 MHz, DMSO-*d*_6_) δ 175.2 (C=O), 143.4 (Ar), 128.8 (Ar), 128.4 (Ar), 128.4 (Ar), 128.4 (Ar), 126.7 (Ar), 50.7 (CH(CH_2_CH_2_CH_3_)_2_), 45.5 (NHCH_2_CH), 43.4 (CHCH_2_NH), 35.3 (CH_3_CH_2_CH_2_CHCH_2_CH_2_CH_3_), 20.4 (CH_3_CH_2_CH_2_CHCH_2_CH_2_CH_3_), 14.4 (CH_3_CH_2_CH_2_CHCH_2_CH_2_CH_3_). HRMS electrospray ionization (ESI) *m*/*z* calcd for [M + Na]^+^ C_22_H_29_NONa^+^ = 346.2141, found 346.2131 (mass error ∆m = −2.89 ppm). IR (KBr) ν_max_., cm^−1^: 3331 *ν* (N-H), 2952 *ν_as_* (CH_3_), 2923 *ν_as_* (CH_2_), 2871 *ν_s_* (CH_3_), 1640, 1601 *ν* (C=O), 1456 *δ_as_* (CH_3_), 1379 *δ_s_* (CH_3_).

##### *N*-(2,2-Bis(3,4-dimethoxyphenyl)ethyl)-2-propylpentanamide **3h**

White solid (m.p. 130–131 °C, crystalized in diethyl ether), yield 92% (0.407 g), *R_f_* = 0.61 (diethyl ether), ^1^H NMR (600 MHz, DMSO-*d*_6_) δ 7.74 (t, *J* = 5.7 Hz, 1H), 6.88 (d, *J* = 2.1 Hz, 2H), 6.84 (d, *J* = 8.4 Hz, 2H), 6.77 (dd, *J* = 8.4, 2.1 Hz, 2H), 4.07 (t, *J* = 8.1 Hz, 1H), 3.74 (s, 6H), 3.70 (s, 6H), 3.66 (dd, *J* = 8.1, 5.6 Hz, 2H), 2.02 (tt, *J* = 9.6, 4.7 Hz, 1H), 1.38–1.29 (m, 2H), 1.16–1.08 (m, 2H), 1.05–0.94 (m, 4H), 0.72 (t, *J* = 7.3 Hz, 6H). ^13^C NMR (151 MHz, DMSO-*d*_6_) δ 175.1 (C=O), 149.0 (Ar), 147.7 (Ar), 136.2 (Ar), 120.2 (Ar), 112.3 (Ar), 112.3 (Ar), 56.0 (OCH_3_), 55.9 (OCH_3_), 49.9 (CH), 45.6 (CHCH_2_NH), 43.7 (CHCH_2_NH), 35.4 (CH_3_CH_2_CH_2_CHCH_2_CH_2_CH_3_), 20.5 (CH_3_CH_2_CH_2_CHCH_2_CH_2_CH_3_), 14.4 (CH_3_CH_2_CH_2_CHCH_2_CH_2_CH_3_). HRMS electrospray ionization (ESI) *m*/*z* calcd for [M + Na]^+^ C_26_H_37_NO_5_Na^+^ = 466.2564, found 466.2557 (mass error ∆m = −1.50 ppm). IR (KBr) ν_max_., cm^−1^: 3270 *ν* (N-H), 2956 *ν_as_* (CH_3_), 2927 *ν_as_* (CH_2_), 2870 *ν_s_* (CH_3_), 1642 *ν* (C=O), 1464 *δ_as_* (CH_3_), 1384 *δ_s_* (CH_3_).

##### *N*-(2-(1H-Indol-3-yl)ethyl)-2-propylpentanamide **3i**

The compound was synthesized in our previous study [81].

White solid (m.p. 88–89 °C, crystalized in petroleum/diethyl ether = 1/3 *v/v*), yield 95% (0.2734 g), *R_f_* = 0.56 (petroleum/diethyl ether = 1/3 *v*/*v*), ^1^H NMR (600 MHz, DMSO-*d*_6_) δ 10.80 (s, 1H), 7.91 (t, *J* = 5.7 Hz, 1H), 7.55 (d, *J* = 7.8 Hz, 1H), 7.34 (d, *J* = 8.1 Hz, 1H), 7.13 (d, *J* = 2.3 Hz, 1H), 7.07 (ddd, *J* = 8.1, 6.9, 1.2 Hz, 1H), 6.98 (ddd, *J* = 7.9, 6.9, 1.1 Hz, 1H), 3.39–3.32 (m, 2H, overlapped with H_2_O), 2.82 (t, *J* = 7.5 Hz, 2H), 2.17–2.08 (m, 1H), 1.49–1.41 (m, 2H), 1.26–1.21 (m, 2H), 1.20–1.14 (m, 4H), 0.84 (t, *J* = 7.2 Hz, 6H). ^13^C NMR (151 MHz, DMSO-*d*_6_) δ 175.2 (C=O), 136.7 (Ar), 127.7 (Ar), 123.1 (NHC=C), 121.3 (Ar), 118.7 (Ar), 118.6 (Ar), 112.3 (NHC=C), 111.8 (Ar), 45.7 (CH), 35.4 (2× CHCH_2_CH_2_CH_3_), 25.9 (CH_2_CH_2_NH), 20.7 (2× CH_2_CH_3_), 14.5 (2×CH_3_). HRMS electrospray ionization (ESI) *m*/*z* calcd for [M + Na]^+^ C_18_H_26_N_2_NaO^+^ = 309.1937, found 309.1928 (mass error ∆m = −2.91 ppm). IR (KBr) ν_max_., cm^−1^: 3283, 3107 *ν* (N-H), 2956 *ν_as_* (CH_3_), 2927 *ν_as_* (CH_2_), 2873 *ν_s_* (CH_3_), 1614 *ν* (C=O), 1458 *δ_as_* (CH_3_), 1378 *δ_s_* (CH_3_).

### 3.2. Preparation of DES—Urea: Propylene Glycol (1:4) (U^1^PG^4^)

The new DES was synthesized by weighing urea and propylene glycol in a 1:4 molar ratio. The mixture was placed in a vial under a nitrogen atmosphere to prevent moisture absorption from the air. The sample was homogenized and heated at 70 °C in a glycerol bath at 800 rpm for 10 min until a clear, homogeneous liquid was obtained. After the reaction was complete, the mixture was cooled to room temperature and allowed to stand overnight to confirm its stability and the absence of precipitation [82]. The synthesized compounds and the corresponding reference standards were dissolved in the prepared deep eutectic solvent, after which their in vitro biological activities were assessed.

These solvatochromic parameters can be calculated using the following equations, where ν is the wavenumber [83,84,85].

(1) $$E_{NR}=\frac{28591}{\lambda_{max}},\frac{kcal}{mol}$$

(2) $$E_{T}\left(30\right)=\frac{E_{NR}-67.312}{-0.3039},\frac{kcal}{mol}\;\;$$

(3) $$E_{T}^{N}=\frac{E_{T}\left(30\right)-E_{T}(TMS)}{E_{T}\left(H_{2}O\right)-E_{T}(TMS)}=\frac{E_{T}\left(30\right)-30.7}{32.4}$$

(4) $$\alpha =\frac{19.9657-1.0241\pi^{*}-\upsilon_{NR}}{1.6078}$$

(5) $$\beta =\frac{1.0434\upsilon_{ANS}-0.57-\upsilon_{NP}}{2}$$

(6) $$\pi^{*}=\frac{\upsilon_{ANS}-34.12}{-2.4}\;\;\;$$

(7) $$\upsilon =\frac{1}{\lambda_{max}10^{-4}}\;\;\;$$

### 3.3. In Vitro Biological Assessment

#### 3.3.1. Hydrogen Peroxide Scavenging Activity (HPSA)

The hydrogen peroxide scavenging activity was evaluated according to the method of Manolov et al. [86]. A 43 mM H_2_O_2_ solution was prepared in 0.2 M potassium phosphate buffer (pH 7.4). The assay mixture consisted of 0.6 mL H_2_O_2_ solution (43 mM), 1.0 mL of sample or standard solution at different concentrations (20–1000 µg/mL), and 2.4 mL potassium phosphate buffer. The mixtures were vortexed and incubated in the dark at 37 °C for 10 min. Absorbance was measured at 230 nm using a Camspec M508 (Spectronic Camspec Ltd., Leeds, UK) against a blank containing phosphate buffer and H_2_O_2_ without the sample. Ascorbic acid and quercetin were used as reference standards. The percentage of hydrogen peroxide scavenging activity (HPSA) was calculated in comparison with the blank according to the following equation:

(8) $$I,\;\%\;\left(HPSA\right)=\left[\frac{A_{blank}-\left(A_{TS}-A_{CS}\right)}{A_{blank}}\right]\times 100$$

where *A_blank_* is the absorbance of the blank sample, *A_CS_* is the absorbance of the control sample, and *A_TS_* is the absorbance of the test sample.

#### 3.3.2. Hydroxyl Radical-Scavenging Activity (HRSA)

The hydroxyl radical-scavenging activity (HRSA) of the samples was determined according to the method described by Luo et al., based on hydroxyl radical generation via the Fenton reaction. In this assay system, hydroxyl radicals oxidize Fe^2+^ to Fe^3+^, whereas only Fe^2+^ forms a stable red–orange complex with 1,10-phenanthroline, exhibiting maximum absorbance at 536 nm. Thus, the extent of decolorization of the reaction mixture reflects the hydroxyl radical concentration and, consequently, the radical-scavenging capacity of the tested samples [87].

Briefly, the reaction mixture consisted of 1.0 mL 1,10-phenanthroline solution (0.75 mM), 2.0 mL phosphate-buffered saline (0.2 M, pH 7.4), and 1.0 mL sample or standard solution. After thorough mixing, 1.0 mL FeSO_4_·7H_2_O solution (0.75 mM) was added, followed by initiation of the reaction with 1.0 mL H_2_O_2_ solution (0.03%, *v*/*v*). The mixtures were incubated at 37 °C for 60 min in a thermostatically controlled water bath. Subsequently, absorbance was measured at 536 nm (Camspec M508 Ltd., UK) against a reagent blank. A reaction mixture lacking antioxidant served as the negative control, whereas the mixture without H_2_O_2_ was used as the blank. The hydroxyl radical-scavenging activity was calculated using the following equation:

(9) $$I,\%\;\left(HRSA\right)=\;\left[\frac{A_{S}-A_{n}}{A_{b}-A_{n}}\right]\times 100$$

where *A_s_*, *A_n_*, and *A_b_* were the absorbance values determined at 536 nm of the sample, the negative control, and the blank after reaction, respectively. Quercetin was used as positive control.

#### 3.3.3. Inhibition of Albumin Denaturation (IAD)

The in vitro anti-inflammatory activity of the investigated compounds was assessed by determining the inhibition of albumin denaturation (IAD) according to the method of Manolov et al. [88], with slight modifications. Human serum albumin was employed as the protein substrate, and a 1% (*w*/*v*) albumin solution was prepared in distilled water adjusted to pH 7.4. To minimize potential interference associated with conventional organic solvents and to preserve albumin stability, a pharmacologically relevant deep eutectic solvent (DES) was utilized as the solubilizing medium [89]. For this purpose, a novel DES composed of urea and propylene glycol in a molar ratio of 1:4 (U^1^PG^4^) was prepared. Test compounds and reference standards were initially dissolved in U^1^PG^4^ to obtain stock solutions at a final concentration of 1300 μg/mL. Subsequently, working solutions in the concentration range of 5–1000 μg/mL were prepared using phosphate-buffered saline (PBS).

The reaction mixture consisted of 2.0 mL of sample or standard solution at the desired concentration and 1.0 mL of 1% albumin solution. The mixtures were incubated at 37 °C for 15 min, followed by thermal denaturation at 70 °C for an additional 15 min in a thermostatically controlled water bath. After cooling to room temperature, turbidity was measured at 660 nm using a Camspec M508. Ibuprofen served as the reference anti-inflammatory agent. All measurements were performed in triplicate.

The percentage inhibition of albumin denaturation was calculated relative to the control. To further characterize the in vitro anti-inflammatory potential of the synthesized amine–valproic acid derivatives, dose–response relationships were analyzed using nonlinear regression models, specifically four-parameter logistic (4PL, Equation (10)) and five-parameter logistic (5PL, Equation (11)) functions [90,91,92].

(10) $$Y=A_{2}+\frac{A_{1}-A_{2}}{\;10^{Hill\;slope\times \left({logIC}_{50}-x\right)}+1}\;$$

(11) $$Y=A_{2}+\frac{A_{1}-A_{2}}{{\left[10^{Hill\;slope\times \left({logIC}_{50}-x\right)}+1\right]}^{s}}$$

#### 3.3.4. Statistical Analysis

All experiments were conducted in triplicate, and the results are presented as mean ± standard deviation (SD). Statistical analyses were performed using GraphPad Prism 10.4.0 and Microsoft Excel. Statistical significance was established at *p* < 0.05. The IC_50_ values were calculated as the mean of three independent determinations.

To evaluate the inhibitory effects of the synthesized amine–valproic acid derivatives on albumin denaturation, dose–response data were analyzed using nonlinear regression models. The choice of the 5PL model over the 4PL model was made based on the Extra sum-of-squares F-test (*F*, (*DFn*, *DFd*)), in which statistical significance was determined at a threshold of *p* < 0.05.

Comparative assessment of the four-parameter logistic (4PL) and five-parameter logistic (5PL) models was performed using statistical criteria that account for differences in model complexity and goodness of fit. The adjusted coefficient of determination ($R_{adj}^{2}$), Akaike information criterion (*AIC*), and residual sum of squares (*RSS*) were calculated based on the same experimental dataset [91,93],

(12) $$RSS=\sum_{i=1}^{n}{\left(y_{i}-{\hat{y}}_{i}\right)}^{2}$$

(13) $$AIC=nln\left(\frac{RSS}{n}\right)+2k\;$$

(14) $$R_{adj}^{2}=1-\frac{RSS}{TSS}\;\;\;$$

where $y_{i}$ and ${\hat{y}}_{i}$ are the observed and predicted values of the model, respectively; *n*—number of observations; *k*—number of parameters; *TSS*—total sum of squares.

These statistical parameters enabled the identification of the most appropriate mathematical model for describing the inhibition of albumin denaturation (IAD).

### 3.4. In Silico Evaluation

#### 3.4.1. DFT Calculations

For investigation of the electronic and structural characteristics of the compounds **3a**–**i**, DFT in silico calculations were performed with the ωB97X-D functional method and the 6-31G* basis set using Spartan 24 1.1.0 (Wavefunction, Inc., Irvine, CA, USA).

#### 3.4.2. Molecular Docking

To investigate the binding behavior of ligands **3a**–**i** toward human serum albumin (HSA), molecular docking calculations were performed using two complementary pieces of docking software, AutoDock Vina 1.1.2 (ADV) and AutoDock 4.2 (AD) [94,95]. The application of both software packages, which rely on different scoring functions and search algorithms, enabled cross-validation of the predicted binding modes and minimized the likelihood of obtaining false-positive docking results [96,97,98,99]. The crystallographic structure of HSA was obtained from the Protein Data Bank (PDB ID: 7JWN), consistent with the model employed in our previous investigations [100,101]. Protein preprocessing included the addition of polar hydrogen atoms and assignment of partial atomic charges according to previously reported procedures [100,102]. The files of the ligands were constructed using Avogadro [103], while ligand and receptor preparation steps were completed with AutoDockTools [95]. Docking experiments focused on four major ligand-binding regions of HSA, namely Sudlow site I (subdomain IIA), Sudlow site II (subdomain IIIA), site III, and the cleft region, selected due to their recognized importance in drug–protein interactions. The grid box center coordinates were defined at x = 30.62, y = 25.50, z = 12.43 for Sudlow site I; x = 5.95, y = 18.22, z = 21.06 for Sudlow site II; x = 30.15, y = 26.98, z = 37.99 for site III; and x = 20.89, y = 21.74, z = 22.43 for the cleft region respectively. For the docking protocol, cubic search spaces of 20 Å per side were applied in AutoDock Vina, whereas for AutoDock 54 Å per side with a spacing of 0.375 Å was used [100,104,105,106,107,108,109]. During the simulations, AutoDock Vina generated 20 docking poses for each ligand at every investigated binding site, while AutoDock calculations produced 200 conformational poses per site. The resulting docking poses and protein–ligand interactions were further analyzed and visualized with UCSF Chimera [110].

#### 3.4.3. Molecular Dynamics

For each ligand, the top-ranked binding pose in site 3 identified by AutoDock Vina was used to create the corresponding chimeric human serum albumin (HSA) complex. Molecular dynamics simulations were subsequently performed using GROMACS 2023 with the CHARMM36 force field and the TIP3P water model in an orthorhombic simulation box [111,112,113]. Ligand parameters were generated using CGenFF, while system preparations such as charge neutralization and energy minimization were carried out according to previously reported procedures [114,115,116]. Each system was simulated for 100 ns to evaluate the stability of the binding of the compounds in site 3 of the albumin. Because of the inconclusive results of the molecular dynamics simulations after the initial 100 ns simulation period for the complexes of compounds **3b**, **3c**, **3d**, and **3i**, they were extended to 150 ns. Calculations were run on a workstation equipped with an AMD Ryzen 9 7900 processor and an NVIDIA RTX 3060 GPU using CUDA 12.8 acceleration under Debian Linux 12. Visualization was performed with VMD 1.9.4 [117], whereas trajectory analyses were conducted using the GROMACS analysis tools.

#### 3.4.4. Molecular Mechanics—Poisson–Boltzmann Surface Area

The frames between 75 ns and 100 ns of the molecular dynamics simulations of the HSA-**3a**–**i** complexes were analyzed for computing the average free binding energy of the ligands to the amino acids of HSA found in within 5 Å, using the energy decomposition strategy, based on the Molecular Mechanics—Poisson–Boltzmann Surface Area (MM-PBSA) by gmx_MMPBSA, based on the formula ΔGbinding = Gcomplex − (Greceptor + Gligand) [118,119,120,121].

#### 3.4.5. Determination of Lipophilicity as cLogP

The lipophilicity of the compounds was calculated using the software ACD/ChemSketch/LogP Predictor v.14.08.

#### 3.4.6. Experimental Determination of Lipophilicity (R_M_)

The method employed for assessing the lipophilicity of VPA derivatives followed the procedure outlined by Hadjipavlou-Litina [122].

## 4. Conclusions

A series of nine amino–valproic acid-based hybrids were successfully synthesized using a rapid mechanochemical approach, providing an efficient and operationally simple route to structurally diverse derivatives. The synthesized compounds were formulated in a newly developed deep eutectic solvent based on urea and propylene glycol (1:4), which exhibited high polarity and favorable solvatochromic properties. Biological evaluation revealed that several derivatives possessed notable antioxidant and anti-inflammatory activities. Among them, compound **3h** consistently demonstrated the highest activity in the hydrogen peroxide scavenging, hydroxyl radical scavenging, and albumin denaturation assays, surpassing the reference standard ibuprofen in the anti-inflammatory model and exhibiting activity comparable to or greater than that of quercetin in the antioxidant assays. Structure–activity relationship analysis indicated that the presence of methoxy substituents on the aromatic rings positively influenced biological activity. Statistical analysis demonstrated that the five-parameter logistic model provided a superior description of the concentration–response relationships compared with the conventional four-parameter model. Lipophilicity studies and in silico calculations further supported the favorable physicochemical characteristics of the investigated compounds. Overall, the results identify compound **3h** as the most promising member of the series and highlight amino–valproic acid hybrids as attractive candidates for further pharmacological investigation.

## Figures and Tables

**Table 1:** Yields of compounds **3a**–**i**.

Compounds 3	R	R^1^	R^2^	Yield, %
**a**	H	H	H	97
**b**	H	Cl	H	93
**c**	H	OCH_3_	H	96
**d**	OCH_3_	H	H	97
**e**	OCH_3_	OCH_3_	H	93
**f**	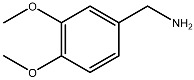			97
**g**	H	H	C_6_H_5_	97
**h**	OCH_3_	OCH_3_	3,4-(OCH_3_)_2_-C_6_H_3_	92
**i**	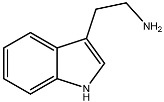			95

**Table 2:** Dose–response parameters and IC_50_ values for standard ibuprofen and synthesized amine–valproic acid derivatives determined by inhibition of albumin denaturation (IAD) using four- and five-parameter logistic models (4PL and 5PL), lipophilicity (***R_M_***), partition coefficient (**cLogP**), and calculated anti-inflammatory activity (**cAnti-I**). The data are presented as mean ± SD (n = 3). Lipophilicity (***R_M_***) is a non-dimensional quantity because it is acquired via thin-layer chromatography and is a function of ***R_f_***. The term “calculated anti-inflammatory activity (**cAnti-I**)” is expressed as Pa (probability “to be active”) and assesses the likelihood that the investigated molecule is a member of the sub-class of active compounds (resembles the most typical molecular structures in a subset of “actives” in the PASS training set). The molecule with the highest level of activity has a value of 1.

Compound	4PL—Model		5PL—Model			*R_M_* ± SD	cLogP	cAnti-I
	*Hill Slope (p)*	IC_50_, µM	*Hill Slope (p)*	*Asymmetry (s)*	IC_50_, µM			Pa
**Ibu**	5.29 ± 0.52	343 ± 13	8.16 ± 1.12	0.30 ± 0.04	356 ± 21	1.11 ± 0.010	3.720	0.901
**3a**	4.42 ± 0.34	305 ± 8	8.94 ± 1.63	0.21 ± 0.05	323 ± 16	0.78 ± 0.012	3.973	0.267
**3b**	3.98 ± 0.34	252 ± 3	5.12 ± 0.90	0.42 ± 0.09	255 ± 3	0.81 ± 0.006	4.686	-
**3c**	4.73 ± 0.14	268 ± 5	11.81 ± 2.24	0.18 ± 0.06	286 ± 15	0.73 ± 0.010	3.892	0.298
**3d**	3.94 ± 0.18	252 ± 3	4.91 ± 0.34	0.57 ± 0.14	253.5 ± 1.9	0.79 ± 0.01	3.892	0.314
**3e**	5.03 ± 0.62	243 ± 6	8.57 ± 0.25	0.24 ± 0.04	251.9 ± 1.3	0.83 ± 0.005	3.631	0.269
**3f**	4.74 ± 0.37	259 ± 6	9.07 ± 1.15	0.25 ± 0.04	272 ± 8	1.01 ± 0.010	3.562	0.33
**3g**	4.16 ± 0.54	228.90 ± 0.79	7.97 ± 0.95	0.25 ± 0.03	233 ± 4	1.04 ± 0.005	5.411	0.267
**3h**	4.92 ± 0.49	169 ± 7	8.01 ± 0.95	0.28 ± 0.01	180 ± 22	0.90 ± 0.010	4.727	0.269
**3i**	4.58 ± 0.31	244 ± 8	6.47 ± 0.68	0.38 ± 0.03	250 ± 16	0.82 ± 0.011	3.963	-

**Table 3:** The energy levels of the frontier molecular orbitals of compounds **3a**–**i** (eV).

Compound	Vacuum		Nonpolar Solvent (ε = 7.43)		Polar Solvent (ε = 37.22)		Water	
	HOMO	LUMO	HOMO	LUMO	HOMO	LUMO	HOMO	LUMO
**3a**	−8.58	1.78	−8.55	1.87	−8.54	1.88	−8.54	1.89
**3b**	−8.77	1.41	−8.71	1.55	−8.69	1.57	−8.69	1.57
**3c**	−8.03	1.75	−7.97	1.80	−7.97	1.81	−7.96	1.81
**3d**	−7.89	1.79	−7.84	1.84	−7.83	1.85	−7.83	1.85
**3e**	−7.50	2.04	−7.49	2.06	−7.50	2.06	−7.50	2.06
**3f**	−7.44	2.06	−7.53	2.02	−7.54	2.01	−7.55	2.01
**3g**	−8.34	1.56	−8.38	1.58	−8.38	1.58	−8.38	1.58
**3h**	−7.59	1.72	−7.70	1.67	−7.71	1.65	−7.72	1.65
**3i**	−7.45	1.55	−7.39	1.59	−7.37	1.60	−7.37	1.60

**Table 4:** Some molecular descriptors of compounds **3a**–**i**.

Compound	LogP	Area (Å^2^)	Volume (Å^3^)	PSA (Å^2^)	Dipole (D)
**3a**	4.18	327.08	296.71	23.934	3.17
**3b**	4.74	342.50	310.35	24.291	1.95
**3c**	4.05	356.40	323.57	30.998	3.09
**3d**	4.05	356.65	323.65	31.036	3.52
**3e**	3.93	385.56	350.42	37.268	3.19
**3f**	3.65	359.81	331.31	38.127	2.94
**3g**	5.77	396.95	378.97	23.918	3.09
**3h**	5.26	507.08	486.62	49.402	3.09
**3i**	4.10	357.77	329.38	36.481	4.16

**Table 5:** The binding affinity (ΔG) of the compounds **3a**–**i** to the four targeted HSA sites—AutoDock Vina (kcal/mol).

Compound	Cleft	Sudlow 1	Sudlow 2	Site 3
**3a**	−7.6	−6.6	−7.7	−8.5
**3b**	−7.9	−6.8	−8.0	−8.9
**3c**	−7.8	−6.9	−7.8	−8.7
**3d**	−7.3	−6.9	−7.6	−8.3
**3e**	−7.2	−6.8	−7.5	−8.3
**3f**	−7.2	−6.5	−7.6	−8.2
**3g**	−8.0	−7.9	−8.2	−9.9
**3h**	−7.6	−7.4	−7.8	−9.2
**3i**	−7.9	−7.2	−8.4	−9.2

**Table 6:** The binding affinity (ΔG) of the compounds **3a**–**i** to the four targeted HSA sites—AutoDock (kcal/mol).

Compound	Cleft	Sudlow 1	Sudlow 2	Site 3
**3a**	−6.20	−6.56	−6.82	−7.65
**3b**	−6.43	−6.58	−7.38	−8.14
**3c**	−6.33	−6.35	−7.20	−7.94
**3d**	−6.33	−6.47	−6.92	−8.44
**3e**	−6.37	−6.44	−6.68	−8.02
**3f**	−6.32	−6.52	−6.96	−8.01
**3g**	−7.51	−7.42	−8.26	−9.84
**3h**	−6.92	−6.75	−8.53	−9.77
**3i**	−7.31	−6.67	−7.93	−8.94

**Table 7:** The main descriptors of the stability of the complexes in the molecular dynamics simulation.

Ligand in Complex	RMSD Ligand (nm)	RMSD HSA Backbone (nm)	Radius of Gyration of HSA (nm)	Ligand–HSA Hydrogen Bonds (no/ns)
**3a** ^1^	0.47	0.23	2.77	0.84
**3b** ^2^	0.57	0.41	2.74	0.13
**3c** ^2^	0.63	0.37	2.73	0.89
**3d** ^2^	0.53	0.34	2.74	0.47
**3e** ^1^	0.39	0.24	2.78	1.25
**3f** ^1^	0.27	0.28	2.78	0.06
**3g** ^1^	0.46	0.25	2.76	0.87
**3h** ^1^	0.39	0.26	2.77	1.80
**3i** ^2^	0.53	0.36	2.74	0.84
none	N/A	0.38	2.78	N/A

^1^—100 ns duration of simulation; ^2^—150 ns duration of simulation, due to inconclusive results after 100 ns.

**Table 8:** The free energy of binding of compounds **3a**–**i** to HSA site 3 and the decomposition in types of interaction (kcal/mol).

Compound	van der Waals	Electrostatic	Solvation	ΔG Total
**3a**	−32.77	−6.87	11.35	−28.29
**3b**	−39.51	5.48	2.41	−31.62
**3c**	−41.38	−17.37	17.43	−41.32
**3d**	−38.72	−6.85	15.62	−29.94
**3e**	−44.64	−20.52	24.13	−41.03
**3f**	−44.62	−14.85	18.71	−40.76
**3g**	−44.21	−9.06	9.64	−43.64
**3h**	−51.91	−17.69	28.02	−41.58
**3i**	−41.51	−8.73	14.16	−36.08

**Table 9:** The energy decomposition of the interaction of compounds **3a**–**i** with the amino acids from site 3 of HSA (kcal/mol).

Amino Acid	Compound								
	3a	3b	3c	3d	3e	3f	3g	3h	3i
LEU115	−0.98	−0.23	−0.21	−0.89	−0.57	−0.35	−0.89	−0.78	−0.97
VAL116	-	-	-	-	-	-	0.29	−0.33	-
ARG117	-	-	-	2.14	1.08	-	−0.33	0.11	0.67
PRO118	-	-	-	-	-	-	−1.10	−0.49	−0.41
MET123	-	-	-	-	-	-	−1.02	−0.64	−0.57
PHE134	−0.16	−0.61	−0.16	−0.22	−0.24	−0.21	−0.70	−0.49	−0.33
LEU135	−0.20	−0.28	−0.23	−0.52	−0.21	−0.70	−0.74	−0.59	−0.26
TYR138	−0.97	−2.24	−0.92	−1.55	−1.32	−1.41	−2.73	−1.79	−2.02
LEU139	−0.10	−0.31	−0.12	−0.66	−0.27	−1.12	−0.64	−0.45	−0.17
GLU141	−0.16	-	-	−0.42	−0.70	−0.30	−0.58	-	−0.26
ILE142	−1.68	−1.94	0.11	−1.83	−1.73	−2.10	−1.37	−1.64	−1.71
ARG145	-	-	-	-	-	-	0.48	1.07	-
HIS146	-	0.31	-	−0.17	−1.17	−0.72	-	−1.17	−0.12
PHE149	−0.41	-	-	−0.33	−0.61	−0.69	-	−0.25	−0.35
GLU153	−0.10	−0.06	-	−0.17	-	−0.40	-	−0.26	-
LEU154	−0.52	-	-	−0.40	−0.45	−0.91	−0.15	−0.40	−0.45
PHE157	−0.72	−0.22	−0.09	−0.54	−1.09	−1.55	−0.10	−0.26	−0.84
ALA158	−0.12	-	−0.24	−0.12	−0.15	−0.77	−0.16	−0.46	−0.11
TYR161	−0.33	−1.28	−0.17	-	−2.02	−2.02	−3.18	−1.29	−0.75
LYS162	-	−0.69	-	0.13	-	-	-	-	−0.06
PHE165	-	−0.86	-	-	−0.23	-	−0.62	−0.26	−0.35
LEU182	-	−0.87	−0.14	−0.83	−1.58	−0.18	−1.68	−1.97	−0.55
LEU185	-	−0.11	-	-	−0.60	-	-	−0.12	−0.33
ARG186	−0.51	0.18	-	−0.56	−0.23	−1.72	−0.39	0.53	−0.54
GLY189	−0.14	0.25	-	−0.52	−0.72	−0.32	-	-	−0.17
LYS190	−0.55	0.14	-	−0.21	−0.25	−1.70	-	-	−0.36
SER192	-	0.13	-	−0.09	-	-	-	-	0.24
SER193	-	0.10	-	−0.08	−0.27	0.13	-	-	0.27

- Non-interacting or the interaction is less than ±0.05 kcal/mol.

## Data Availability

Data are contained within the article and Supplementary Materials.

## Supplementary Materials

The following supporting information can be downloaded at https://www.mdpi.com/article/10.3390/molecules31142535/s1, Figure S1: ^1^H-NMR spectrum of compound **3a**; Figure S2: ^1^H-NMR spectrum of compound **3b**; Figure S3: ^1^H-NMR spectrum of compound **3f**; Figure S4: ^1^H-NMR spectrum of compound **3g**; Figure S5: ^1^H-NMR spectrum of compound **3h**; Figure S6: ^13^C-NMR spectrum of compound **3a**; Figure S7: ^13^C-NMR spectrum of compound **3b**; Figure S8: ^13^C-NMR spectrum of compound **3f**; Figure S9: ^13^C-NMR spectrum of compound **3g**; Figure S10: ^13^C-NMR spectrum of compound **3h**; Figure S11: IR spectrum of compound **3a**; Figure S12: IR spectrum of compound **3b**; Figure S13: IR spectrum of compound **3f**; Figure S14: IR spectrum of compound **3g**; Figure S15: IR spectrum of compound **3h**; Figure S16: ESI-HRMS of compound **3a**; Figure S17: ESI-HRMS of compound **3b**; Figure S18: ESI-HRMS of compound **3f**; Figure S19: ESI-HRMS of compound **3g**; Figure S20: ESI-HRMS of compound **3h**; Figure S21: HOMO (left) and LUMO (right) of compound **3a**; Figure S22: HOMO (left) and LUMO (right) of compound **3b**; Figure S23: HOMO (left) and LUMO (right) of compound **3c**; Figure S24: HOMO (left) and LUMO (right) of compound **3d**; Figure S25: HOMO (left) and LUMO (right) of compound **3e**; Figure S26: HOMO (left) and LUMO (right) of compound **3f**; Figure S27: HOMO (left) and LUMO (right) of compound **3g**; Figure S28: HOMO (left) and LUMO (right) of compound **3h**; Figure S29: HOMO (left) and LUMO (right) of compound **3i**; Figure S30: Electrostatic-potential map of compound **3a**; Figure S31: Electrostatic-potential map of compound **3b**; Figure S32: Electrostatic-potential map of compound 3c; Figure S33: Electrostatic-potential map of compound **3d**; Figure S34: Electrostatic-potential map of compound **3e**; Figure S35: Electrostatic-potential map of compound **3f**; Figure S36: Electrostatic-potential map of compound **3g**; Figure S37: Electrostatic-potential map of compound **3h**; Figure S38: Electrostatic-potential map of compound **3i**; Figure S39: Analysis of the evolution in time of the complex of the compound **3a** docked in site 3 of HAS; Figure S40: Analysis of the evolution in time of the complex of the compound **3b** docked in site 3 of HAS; Figure S41: Analysis of the evolution in time of the complex of the compound **3c** docked in site 3 of HAS; Figure S42: Analysis of the evolution in time of the complex of the compound **3d** docked in site 3 of HAS; Figure S43: Analysis of the evolution in time of the complex of the compound **3e** docked in site 3 of HAS; Figure S44: Analysis of the evolution in time of the complex of the compound **3f** docked in site 3 of HAS; Figure S45: Analysis of the evolution in time of the complex of the compound **3g** docked in site 3 of HAS; Figure S46: Analysis of the evolution in time of the complex of the compound **3h** docked in site 3 of HAS; Figure S47: Analysis of the evolution in time of the complex of the compound **3i** docked in site 3 of HAS; Figure S48: Binding energy decomposition for compound **3a**; Figure S49: Binding energy decomposition for compound **3b**; Figure S50: Binding energy decomposition for compound **3c**. Figure S51: Binding energy decomposition for compound **3d**; Figure S52: Binding energy decomposition for compound **3e**; Figure S53: Binding energy decomposition for compound **3f**; Figure S54: Binding energy decomposition for compound **3g**; Figure S55: Binding energy decomposition for compound **3h**; Figure S56: Binding energy decomposition for compound **3i**; Table S1: Antioxidant activity (HPSA, HRSA) of amine–valproic acid derivatives **3a**–**i**; Table S2: Statistical comparison of 4PL and 5PL models for anti-inflammatory activity.
