# Supplementary material for: Mechanochemical Synthesis of Valproic Acid-Based Hybrids: Antioxidant, Anti-Inflammatory, and In Silico Evaluation
Source: Molecules. 2026 Jul 21;31(14):2535. doi: 10.3390/molecules31142535 (PMC13414799; doi:10.3390/molecules31142535)
Supplement: Supplementary file 1 [file molecules-31-02535-s001.zip › molecules-4425349-supplementary.pdf]

Supplementary Materials:

Mechanochemical Synthesis of Valproic Acid-Based Hybrids: Antioxidant, Anti-Inflammatory, and In Silico Evaluation

Diyana Dimitrova [1], Iliyan Ivanov [1], Dimitar Bojilov [1], Gabriel Marc [2], Smaranda Oniga [3], Ovidiu Oniga [4], Ovidiu Crișan [2], and Stanimir Manolov [1,*],

Table of Contents:

Table S1. Antioxidant activity (HPSA, HRSA) of amine–valproic acid derivatives **3a-i**. ***page 4***

Table S2. Statistical Comparison of 4PL and 5PL Models for Anti-Inflammatory Activity. ***page 5***

Figure S1. ^{1}H-NMR spectrum of compound **3a**. ***page 6***

Figure S2. ^{1}H-NMR spectrum of compound **3b**. ***page 7***

Figure S3. ^{1}H-NMR spectrum of compound **3f**. ***page 8***

Figure S4. ^{1}H-NMR spectrum of compound **3g**. ***page 9***

Figure S5. ^{1}H-NMR spectrum of compound **3h**. ***page 10***

Figure S6. ^{13}C-NMR spectrum of compound **3a**. ***page 11***

Figure S7. ^{13}C-NMR spectrum of compound **3b**. ***page 12***

Figure S8. ^{13}C-NMR spectrum of compound **3f**. ***page 13***

Figure S9. ^{13}C-NMR spectrum of compound **3g**. ***page 14***

Figure S10. ^{13}C-NMR spectrum of compound **3h**. ***page 15***

Figure S11. IR spectrum of compound **3a**. ***page 16***

Figure S12. IR spectrum of compound **3b**. ***page 17***

Figure S13. IR spectrum of compound **3f**. ***page 18***

Figure S14. IR spectrum of compound **3g**. ***page 19***

Figure S15. IR spectrum of compound **3h**. ***page 20***

Figure S16. ESI-HRMS of compound **3a**. ***page 21***

Figure S17. ESI-HRMS of compound **3b**. ***page 22***

Figure S18. ESI-HRMS of compound **3f**. ***page 23***

Figure S19. ESI-HRMS of compound **3g**. ***page 24***

Figure S20. ESI-HRMS of compound **3h**. ***page 25***

Figure S21. HOMO (left) and LUMO (right) of compound **3a**. ***page 26***

Figure S22. HOMO (left) and LUMO (right) of compound **3b**. ***page 26***

Figure S23. HOMO (left) and LUMO (right) of compound **3c**. ***page 27***

Figure S24. HOMO (left) and LUMO (right) of compound **3d**. ***page 27***

Figure S25. HOMO (left) and LUMO (right) of compound **3e**. ***page 27***

Figure S26. HOMO (left) and LUMO (right) of compound **3f**. ***page 28***

Figure S27. HOMO (left) and LUMO (right) of compound **3g**. ***page 28***

Figure S28. HOMO (left) and LUMO (right) of compound **3h**. ***page 28***

Figure S29. HOMO (left) and LUMO (right) of compound **3i**. ***page 29***

Figure S30. Electrostatic potential map of compound **3a** ***page 29***

Figure S31. Electrostatic potential map of compound **3b**. ***page 30***

Figure S32. Electrostatic potential map of compound **3c**. ***page 31***

Figure S33. Electrostatic potential map of compound **3d**. ***page 32***

Figure S34. Electrostatic potential map of compound **3e**. ***page 32***

Figure S35. Electrostatic potential map of compound **3f**. ***page 33***

Figure S36. Electrostatic potential map of compound **3g**. ***page 33***

Figure S37. Electrostatic potential map of compound **3h**. ***page 34***

Figure S38. Electrostatic potential map of compound **3i**. ***page 35***

Figure S39. Analysis of the evolution in time of the complex of the compound **3a** docked in site 3 of HSA. a) RMSD of heavy atoms of the ligand, b) RMSD of HSA backbone apo (black) and in complex with the ligand (red), c) RG of HSA backbone apo (black) and in complex with the ligand (red), d) hydrogen bonds between the ligand and HSA (5 ns moving average). ***page 36***

Figure S40. Analysis of the evolution in time of the complex of the compound **3b** docked in site 3 of HSA. a) RMSD of heavy atoms of the ligand, b) RMSD of HSA backbone apo (black) and in complex with the ligand (red), c) RG of HSA backbone apo (black) and in complex with the ligand (red), d) hydrogen bonds between the ligand and HSA (5 ns moving average). ***page 37***

Figure S41. Analysis of the evolution in time of the complex of the compound **3c** docked in site 3 of HSA. a) RMSD of heavy atoms of the ligand, b) RMSD of HSA backbone apo (black) and in complex with the ligand (red), c) RG of HSA backbone apo (black) and in complex with the ligand (red), d) hydrogen bonds between the ligand and HSA (5 ns moving average). ***page 38***

Figure S42. Analysis of the evolution in time of the complex of the compound **3d** docked in site 3 of HSA. a) RMSD of heavy atoms of the ligand, b) RMSD of HSA backbone apo (black) and in complex with

the ligand (red), c) RG of HSA backbone apo (black) and in complex with the ligand (red), d) hydrogen bonds between the ligand and HSA (5 ns moving average). **page 39**

Figure S43. Analysis of the evolution in time of the complex of the compound **3e** docked in site 3 of HSA. a) RMSD of heavy atoms of the ligand, b) RMSD of HSA backbone apo (black) and in complex with the ligand (red), c) RG of HSA backbone apo (black) and in complex with the ligand (red), d) hydrogen bonds between the ligand and HSA (5 ns moving average). **page 40**

Figure S44. Analysis of the evolution in time of the complex of the compound **3f** docked in site 3 of HSA. a) RMSD of heavy atoms of the ligand, b) RMSD of HSA backbone apo (black) and in complex with the ligand (red), c) RG of HSA backbone apo (black) and in complex with the ligand (red), d) hydrogen bonds between the ligand and HSA (5 ns moving average). **page 41**

Figure S45. Analysis of the evolution in time of the complex of the compound **3g** docked in site 3 of HSA. a) RMSD of heavy atoms of the ligand, b) RMSD of HSA backbone apo (black) and in complex with the ligand (red), c) RG of HSA backbone apo (black) and in complex with the ligand (red), d) hydrogen bonds between the ligand and HSA (5 ns moving average). **page 42**

Figure S46. Analysis of the evolution in time of the complex of the compound **3h** docked in site 3 of HSA. a) RMSD of heavy atoms of the ligand, b) RMSD of HSA backbone apo (black) and in complex with the ligand (red), c) RG of HSA backbone apo (black) and in complex with the ligand (red), d) hydrogen bonds between the ligand and HSA (5 ns moving average). **page 43**

Figure S47. Analysis of the evolution in time of the complex of the compound **3i** docked in site 3 of HSA. a) RMSD of heavy atoms of the ligand, b) RMSD of HSA backbone apo (black) and in complex with the ligand (red), c) RG of HSA backbone apo (black) and in complex with the ligand (red), d) hydrogen bonds between the ligand and HSA (5 ns moving average). **page 44**

Figure S48. Binding energy decomposition for compound **3a**. Favorable interactions are depicted in black, while unfavorable interactions are depicted in red. **page 45**

Figure S49. Binding energy decomposition for compound **3b**. Favorable interactions are depicted in black, while unfavorable interactions are depicted in red. **page 45**

Figure S50. Binding energy decomposition for compound **3c**. Favorable interactions are depicted in black, while unfavorable interactions are depicted in red. **page 46**

Figure S51. Binding energy decomposition for compound **3d**. Favorable interactions are depicted in black, while unfavorable interactions are depicted in red. **page 46**

Figure S52. Binding energy decomposition for compound **3e**. Favorable interactions are depicted in black, while unfavorable interactions are depicted in red. **page 47**

Figure S53. Binding energy decomposition for compound **3f**. Favorable interactions are depicted in black, while unfavorable interactions are depicted in red. **page 47**

Figure S54. Binding energy decomposition for compound **3g**. Favorable interactions are depicted in black, while unfavorable interactions are depicted in red. **page 48**

Figure S55. Binding energy decomposition for compound **3h**. Favorable interactions are depicted in black, while unfavorable interactions are depicted in red. **page 48**

Figure S56. Binding energy decomposition for compound **3i**. Favorable interactions are depicted in black, while unfavorable interactions are depicted in red. **page 49**

Table S1. Antioxidant activity (HPSA, HRSA) of synthesized amine–valproic acid derivatives (**3a-i**). Results are presented as $IC_{50} \pm SD/\mu M$. Quercetin (Qrc) was used as standard.

**Compounds**	**HPSA**	**HRSA**
	IC₅₀, µM	
**3a**	490 ± 6	278 ± 4
**3b**	399 ± 2	234 ± 5
**3c**	926 ± 27	232 ± 3
**3d**	1110 ± 43	227 ± 4
**3e**	388 ± 6	203 ± 6
**3f**	1647 ± 28	225 ± 5
**3g**	522 ± 18	269 ± 12
**3h**	291 ± 46	163 ± 4
**3i**	5075 ± 238	219 ± 2
**Standard**		
Qrc	225 ± 3	232 ± 4

Table S2. Comparative analysis of the goodness of fit between four-parameter (4PL) and five-parameter (5PL) logistic models for assessing the anti-inflammatory activity of ibuprofen (Ibu) and the new synthesized amine-valproic acid derivatives, including statistical indicators R^2_{adj}, *RSS* and *AIC*. R^2_{adj} – adjusted coefficient of determination; *RSS* – residual sum of squares; *AIC* – Akaike Information Criterion.

**Comparison of Fits**	**Ibu**	**3a**	**3b**	**3c**	**3d**	**3e**	**3f**	**3g**	**3h**	**3i**
Null hypothesis	4PL	4PL	4PL	4PL	4PL	4PL	4PL	4PL	4PL	4PL
Alternative hypothesis	5PL	5PL	5PL	5PL	5PL	5PL	5PL	5PL	5PL	5PL
P value	0.017	0.0002	0.0027	<0.0001	0.0034	0.0079	<0.0001	<0.0001	0.0097	0.0062
Conclusion ($\alpha = 0.05$)					Reject Null hypothesis					
Preferred model	5PL	5PL	5PL	5PL	5PL	5PL	5PL	5PL	5PL	5PL
F (DFn, DFd)	6.715 (1, 19)	21.05 (1, 19)	11.93 (1, 19)	40.38 (1, 19)	5.177 (1, 19)	8.801 (1, 19)	32.78 (1, 19)	30.05 (1, 19)	8.262 (1, 19)	9.065 (1, 19)
R^2_{adj} (4PL/5PL)	0.9954/0.9964	0.9961/0.9981	0.9964/0.9974	0.9958/0.9986	0.9975/0.9979	0.9964/0.9974	0.9969/0.9986	0.9947/0.9978	0.9955/0.9967	0.9965/0.9975
RSS (4PL/5PL)	182/134	128/61	138/84	135/43	84/66	118/80	139/51	173/67	141/98	107/72
AIC (4PL/5PL)	62/58	54/39	55/47	55/31	44/41	52/46	54/35	61/42	56/50	49/43

The comparison between the two models was performed using the Extra sum-of-squares F-test, which assesses whether the addition of the fifth parameter (asymmetry) improves the statistical fit in a way that is mathematically justified. Model selection was based on the levels of statistical significance ($p < 0.05$) and minimization of the Akaike Information Criterion (*AIC*). In the presence of statistical significance, the 5PL model was selected for the final determination of IC_{50} values, providing greater precision in accounting for deviations from standard sigmoid kinetics.

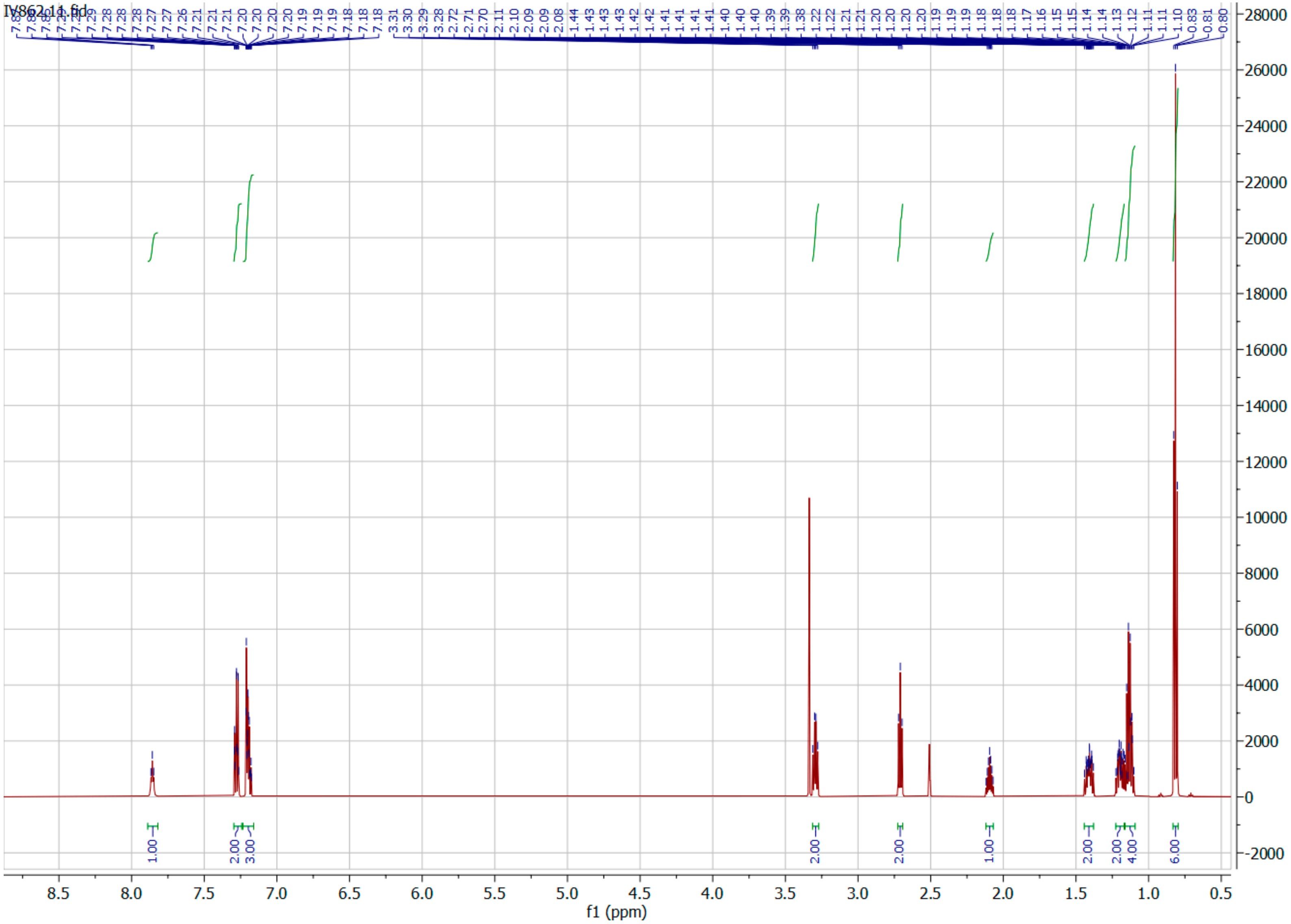

Figure S1. ^{1}H-NMR spectrum of compound **3a**.

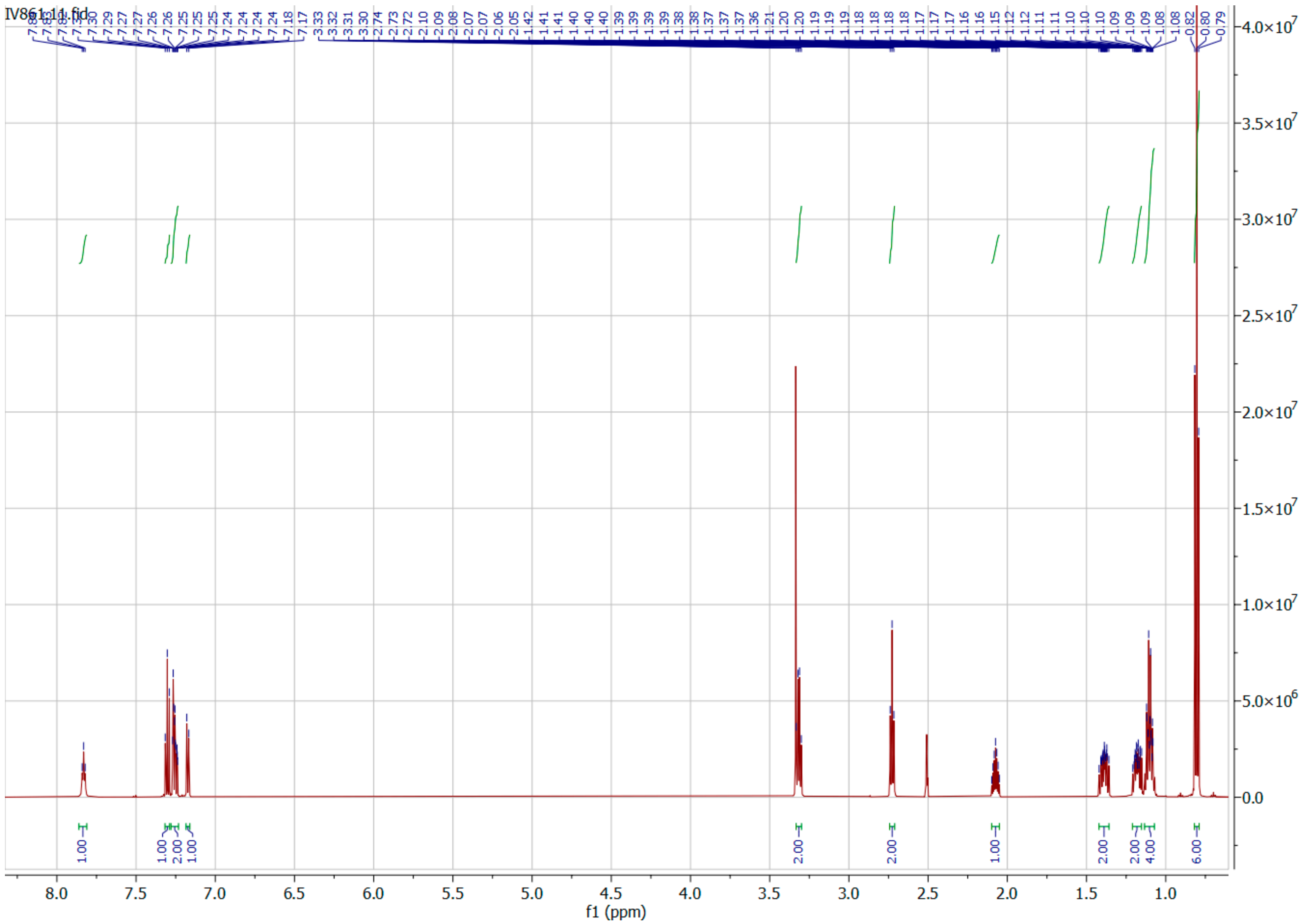

Figure S2. ^{1}H-NMR spectrum of compound **3b**.

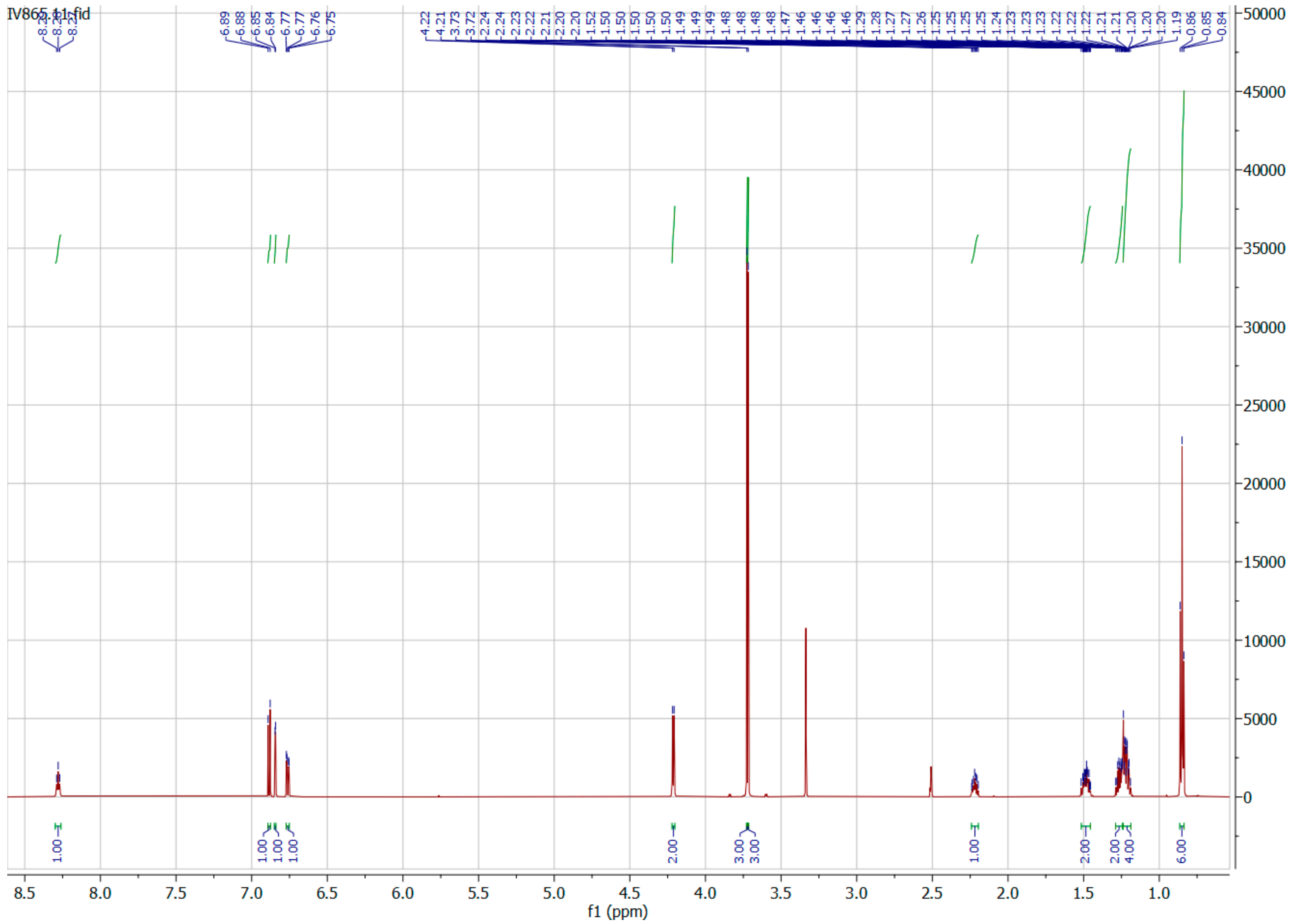

Figure S3. ^{1}H-NMR spectrum of compound **3f**.

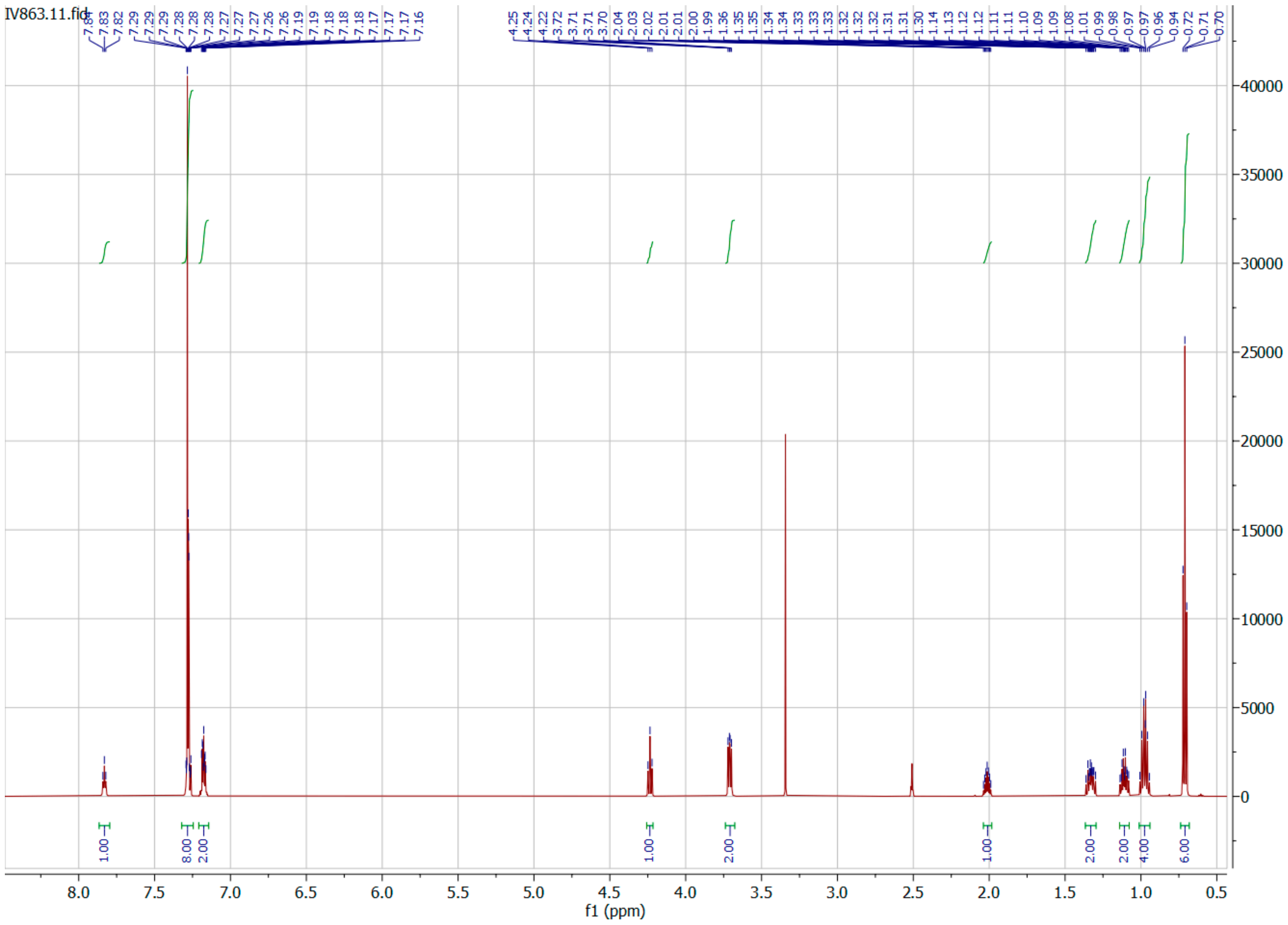

Figure S4. ^{1}H-NMR spectrum of compound **3g**.

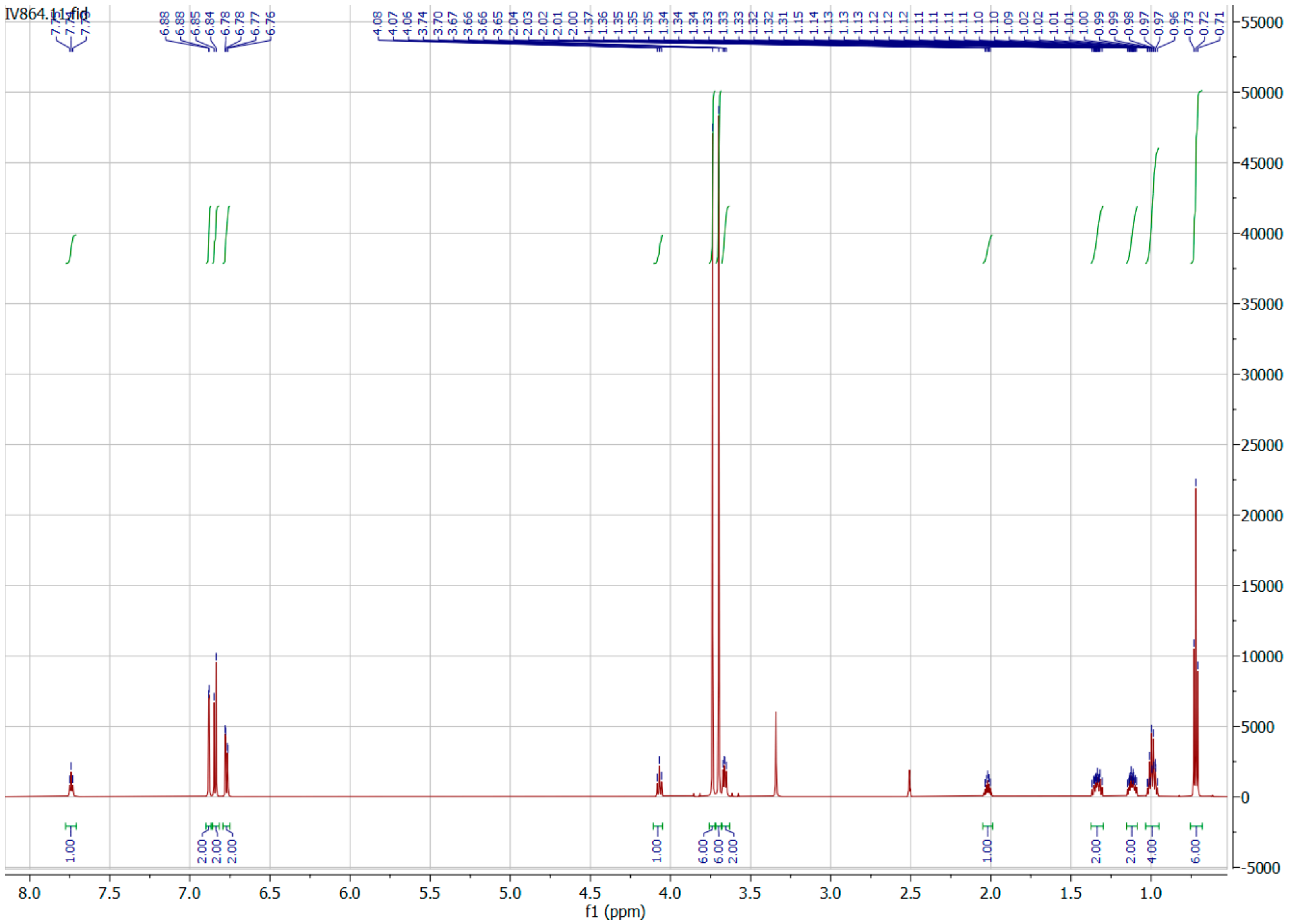

Figure S5. ^{1}H-NMR spectrum of compound **3h**.

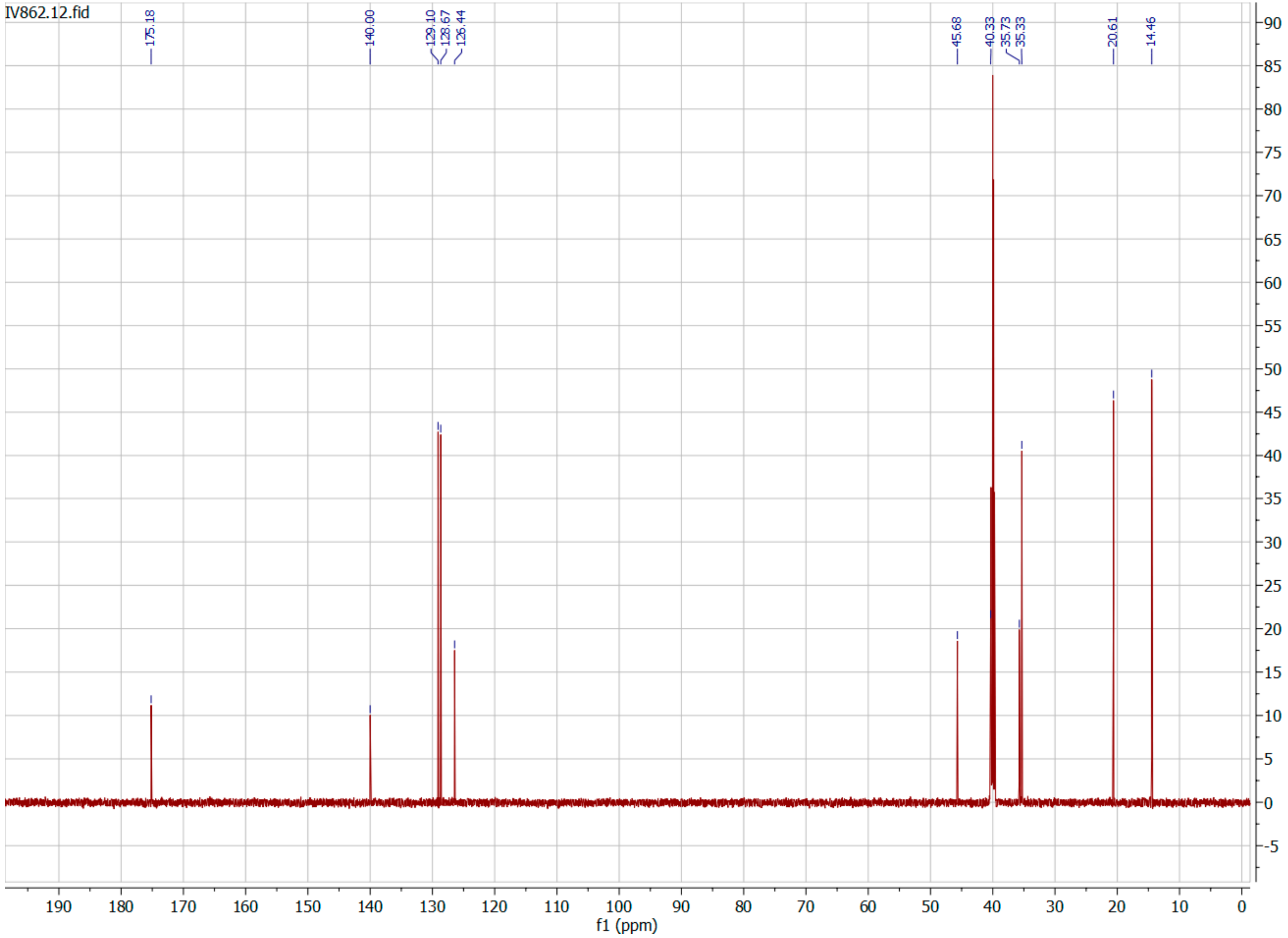

Figure S6. ^{13}C-NMR spectrum of compound **3a.**

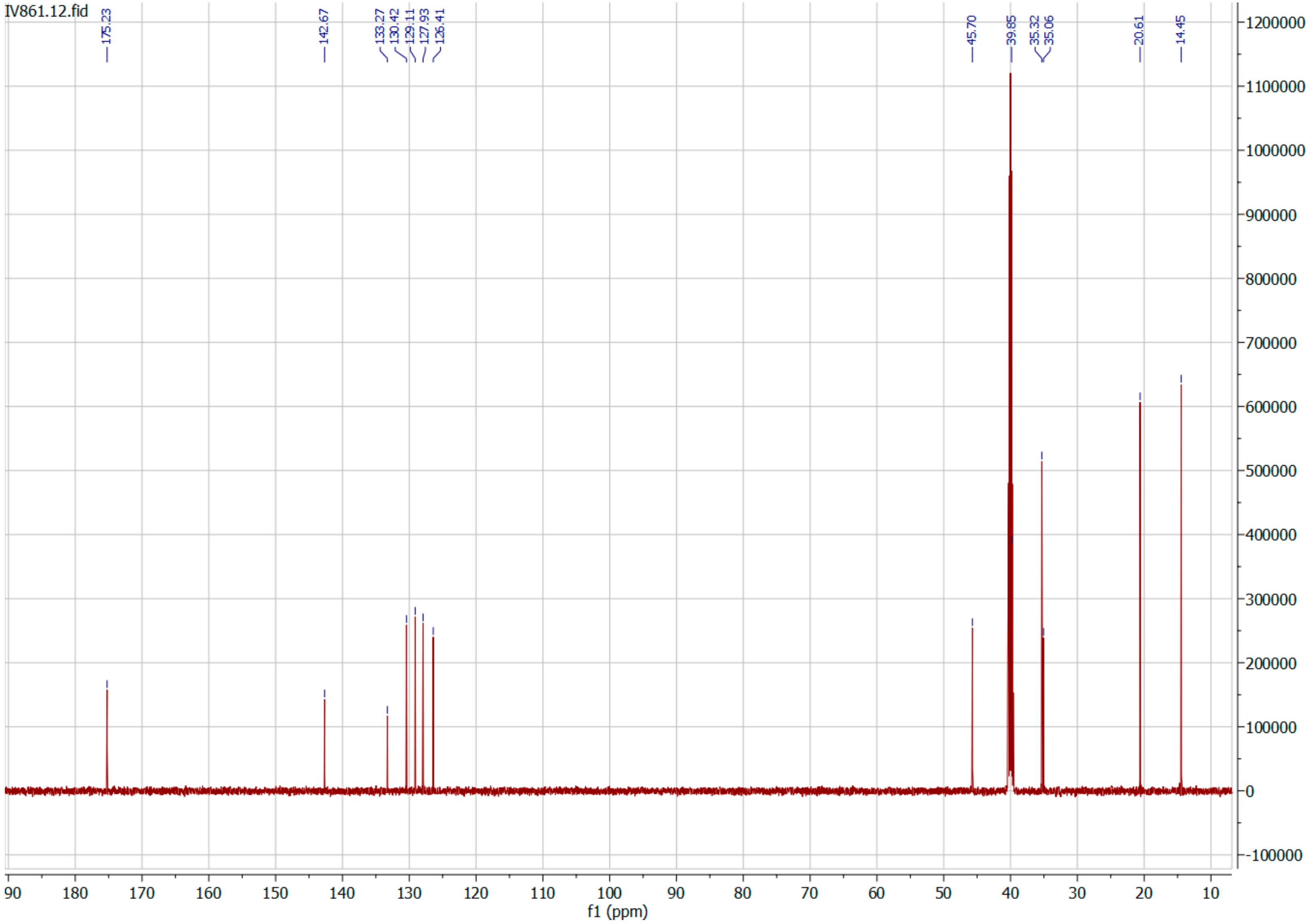

Figure S7. ^{13}C-NMR spectrum of compound **3b.**

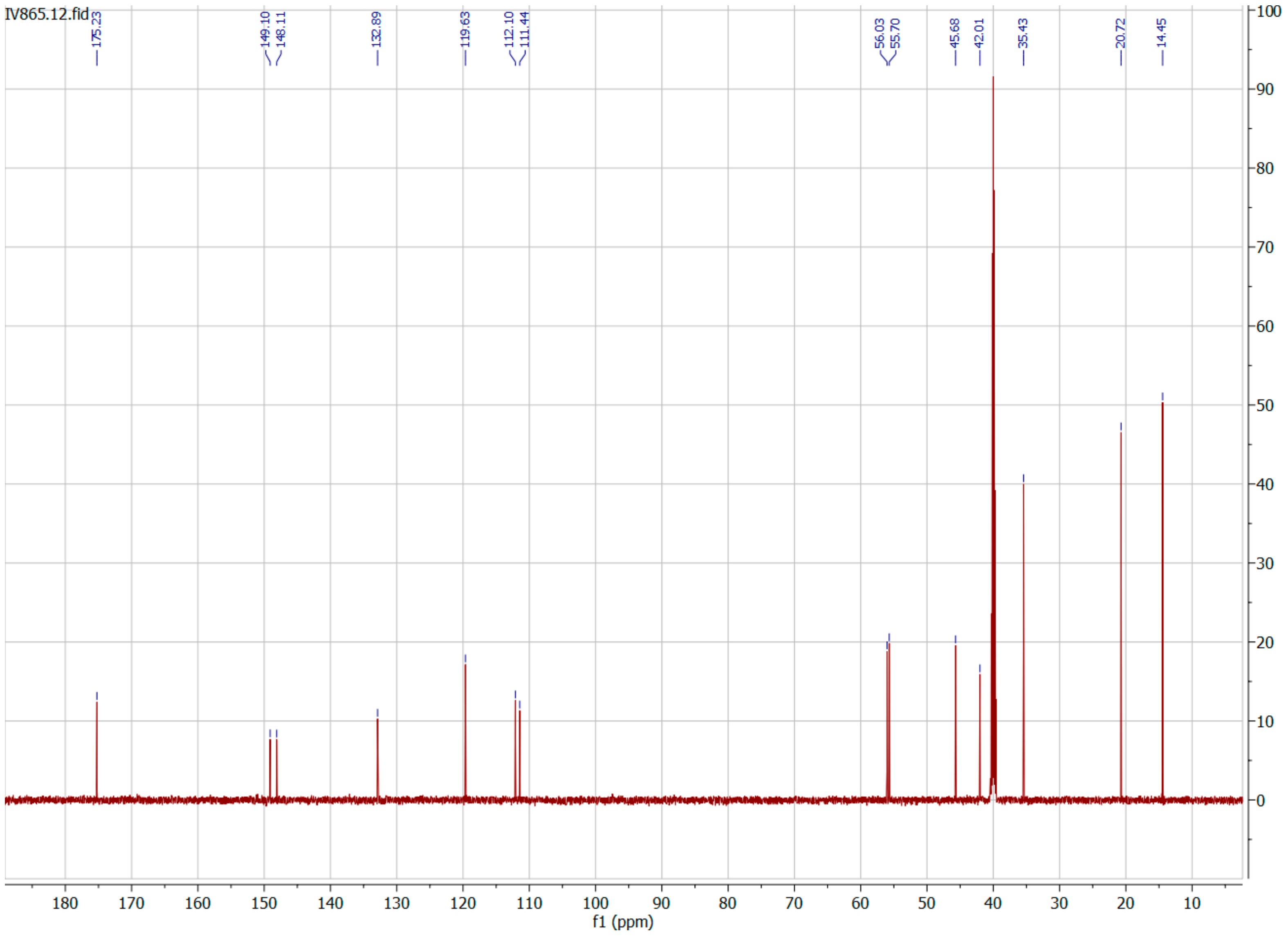

Figure S8. ^{13}C-NMR spectrum of compound **3f**.

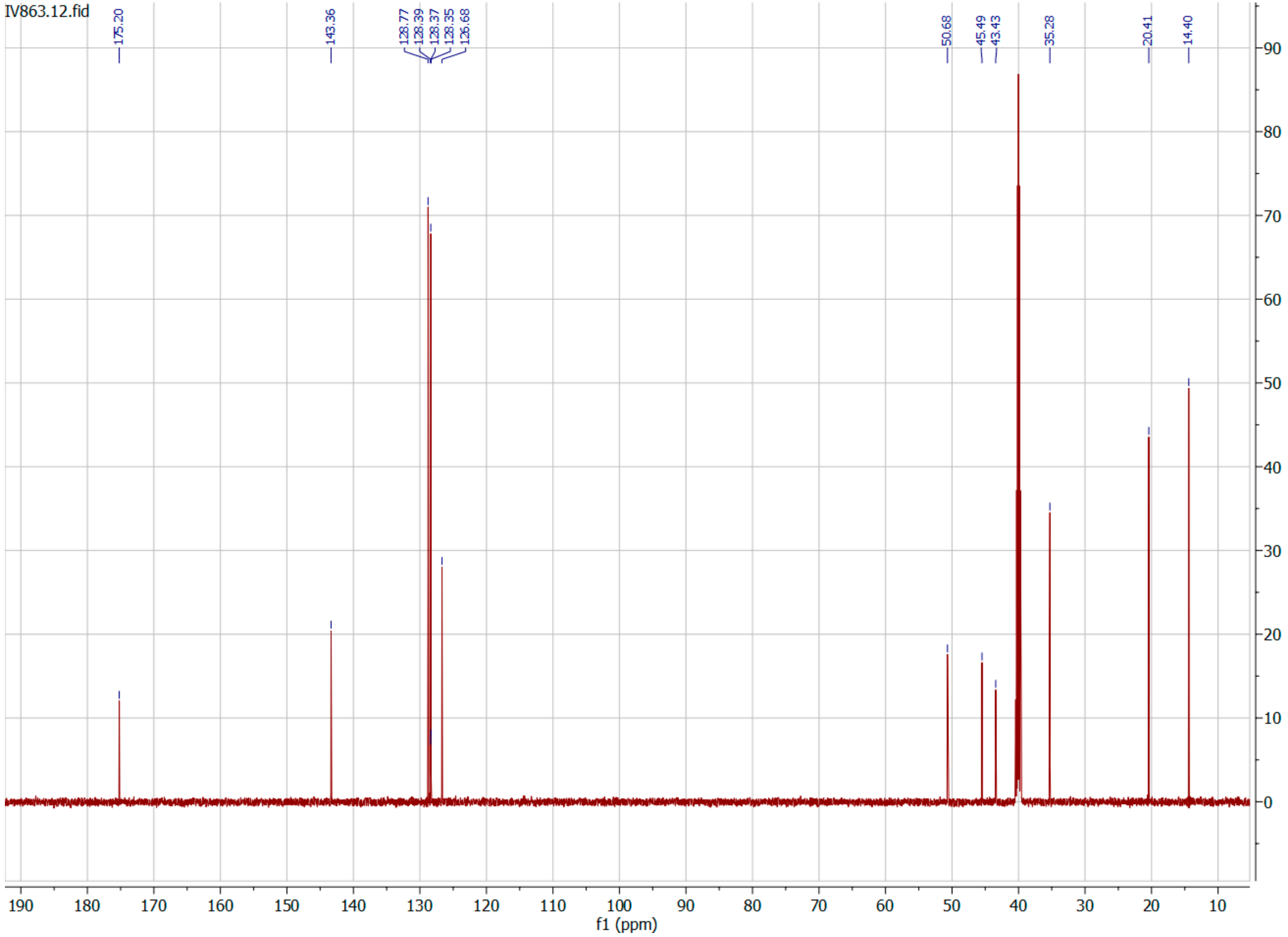

Figure S9. ^{13}C-NMR spectrum of compound **3g**.

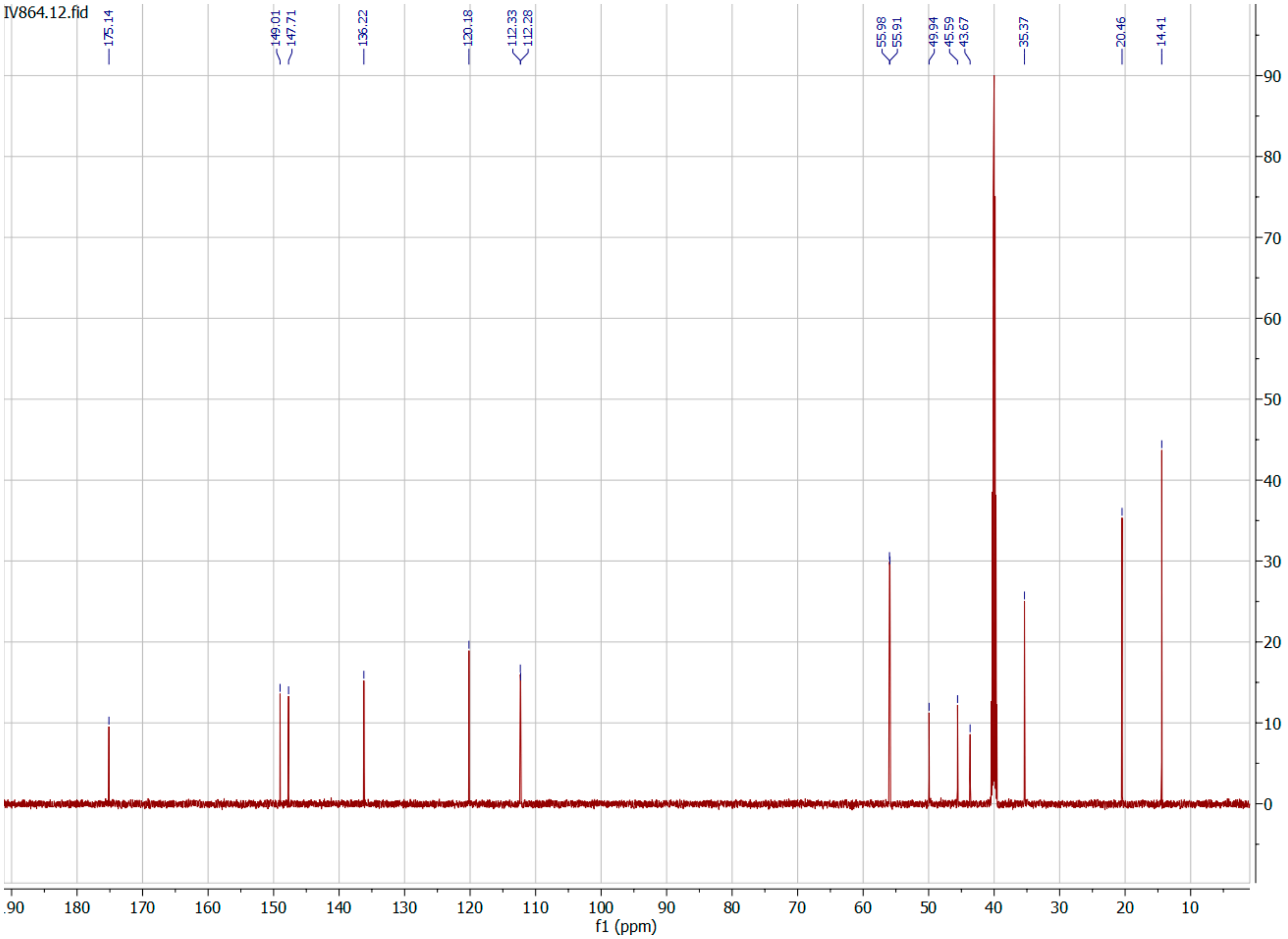

Figure S10. ^{13}C-NMR spectrum of compound **3h**.

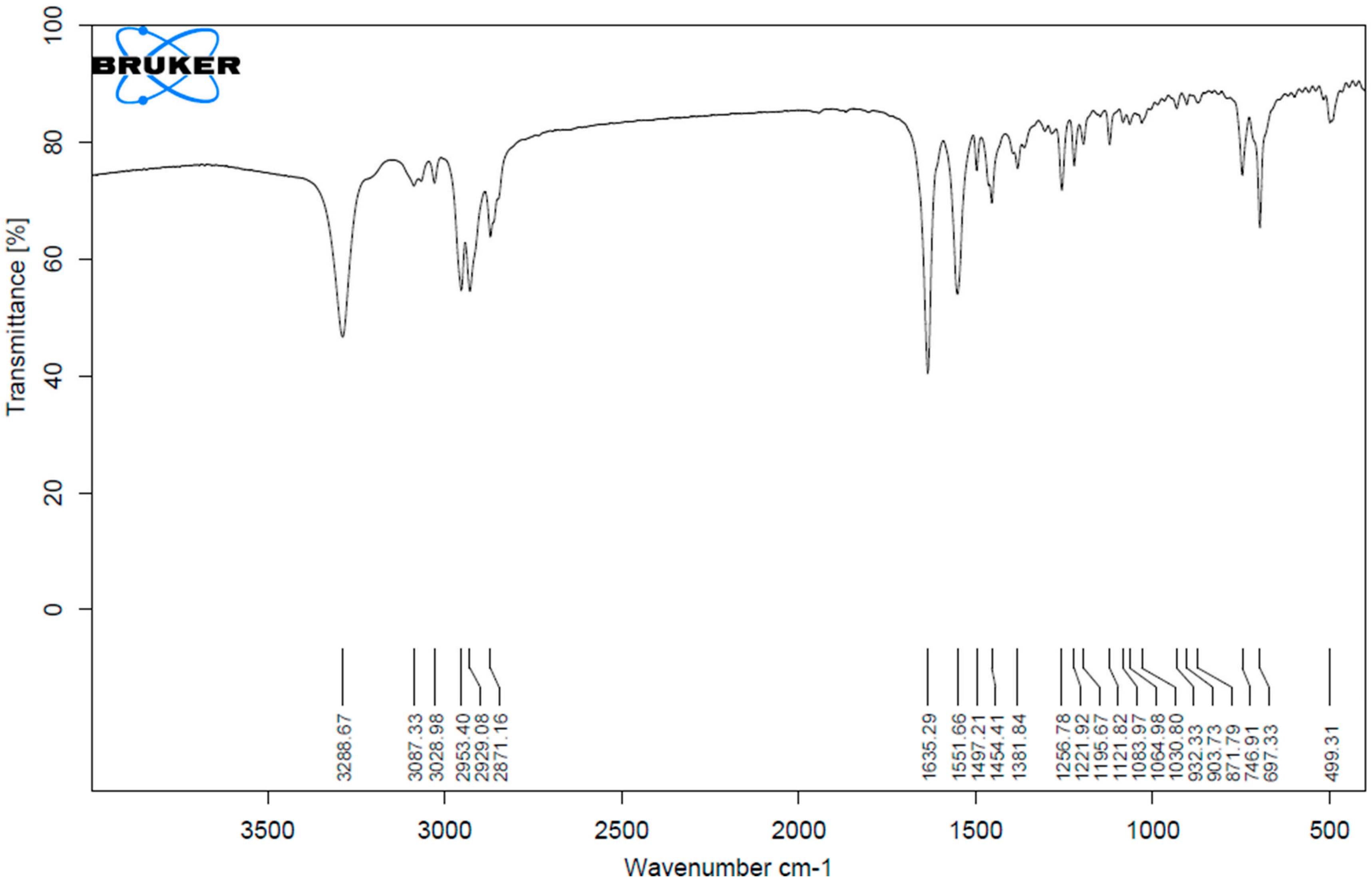

Figure S11. IR spectrum of compound **3a**.

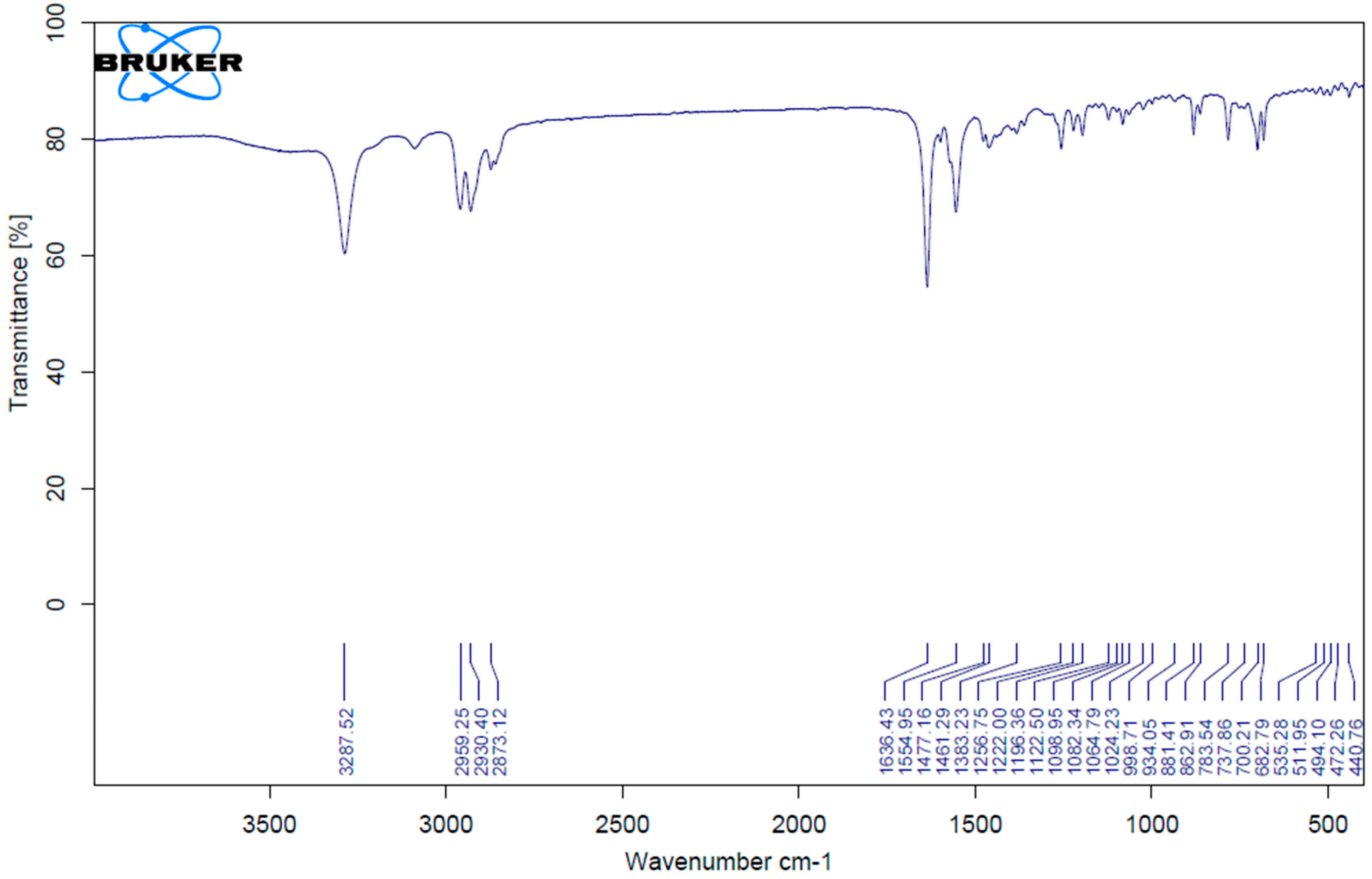

Figure S12. IR spectrum of compound **3b**.

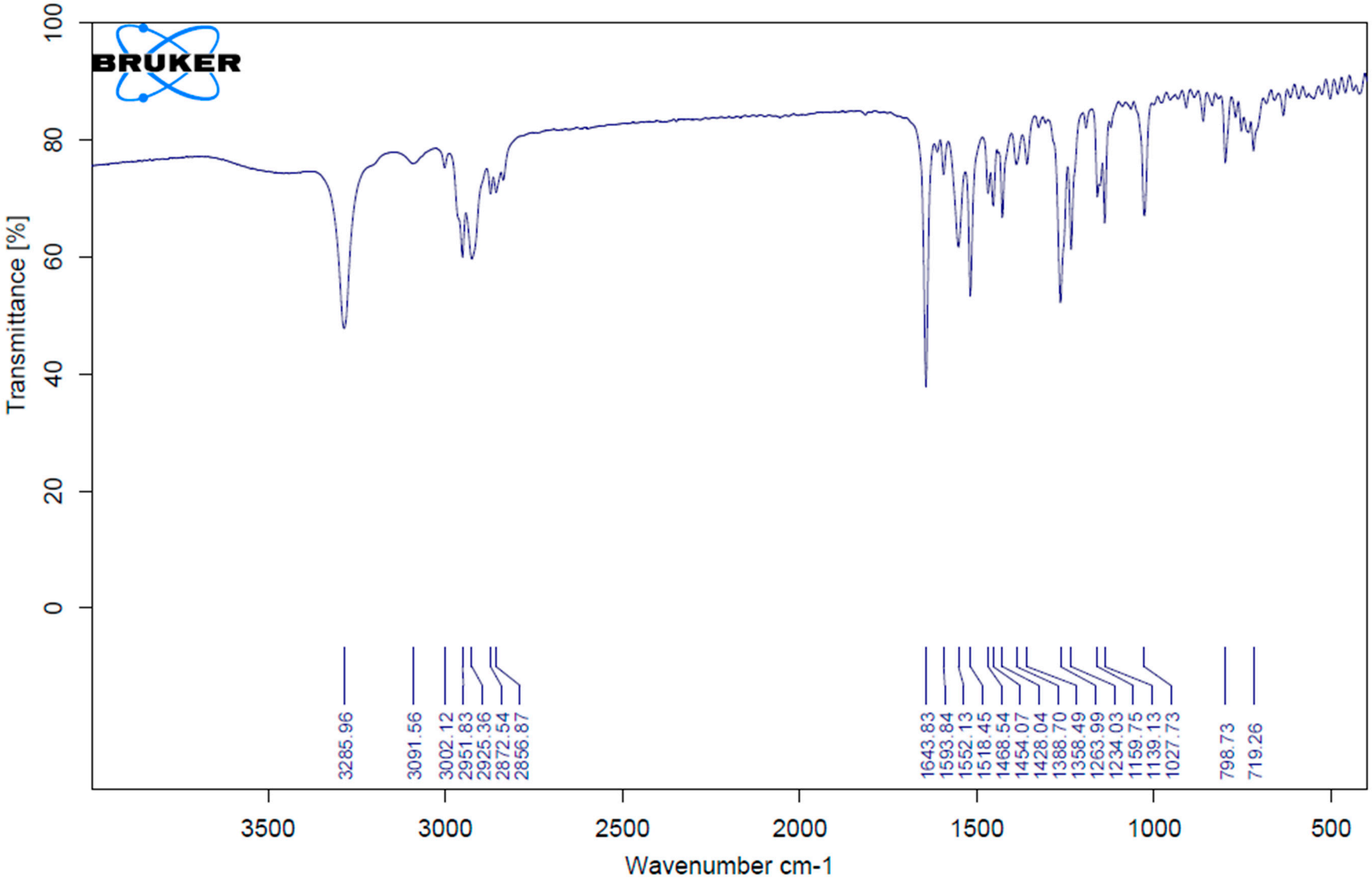

Figure S13. IR spectrum of compound **3f**.

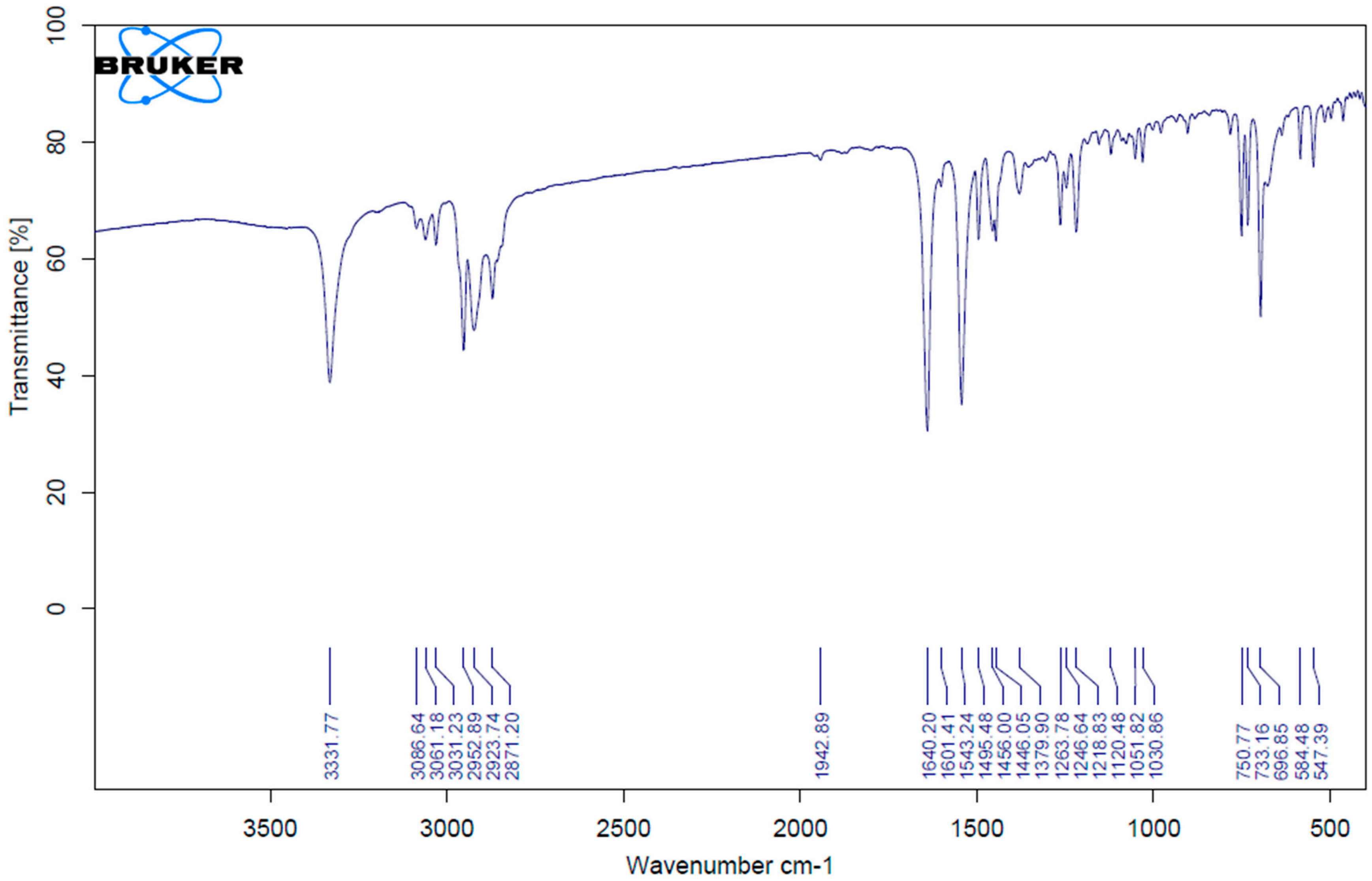

Figure S14. IR spectrum of compound **3g**.

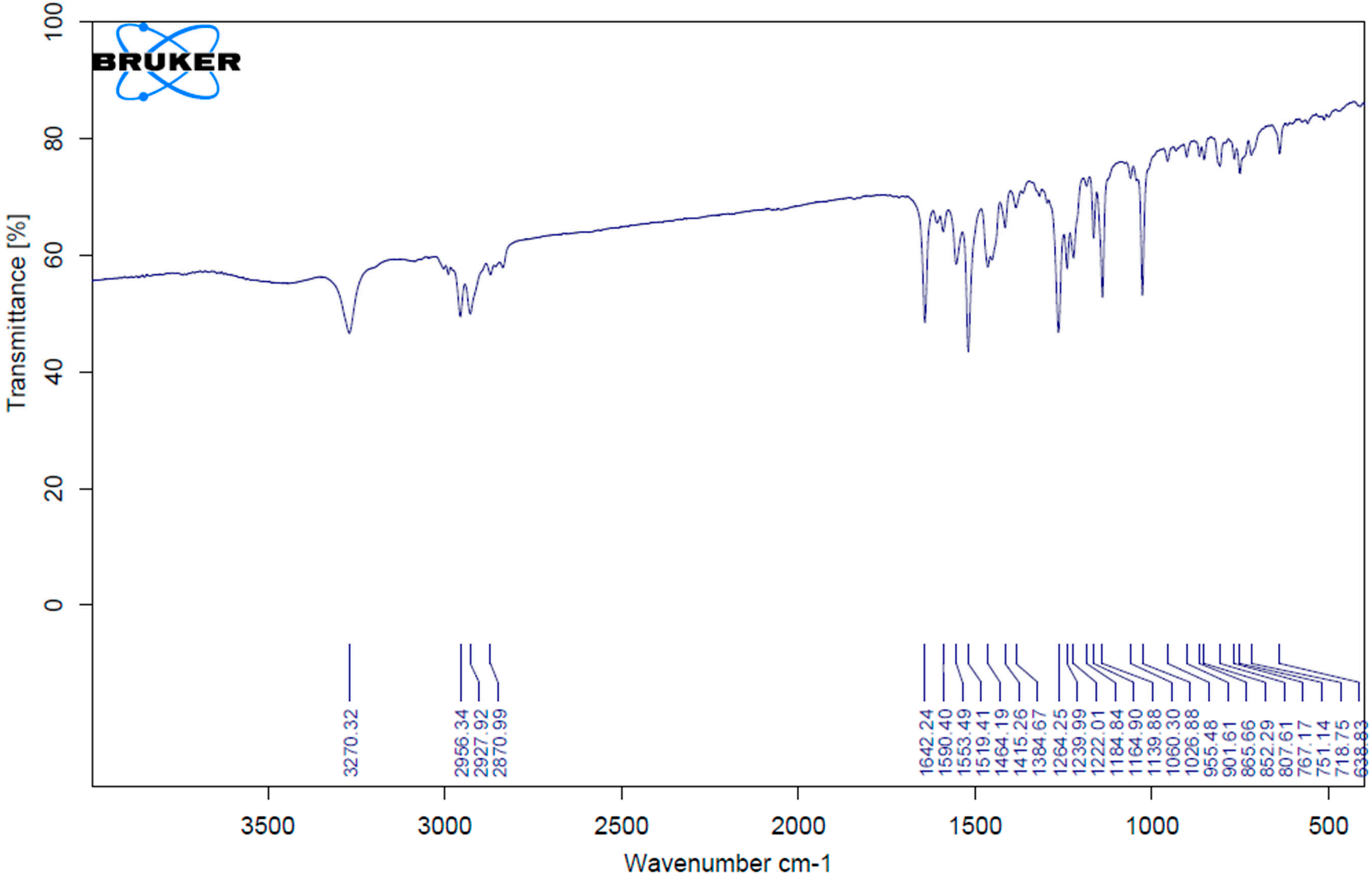

Figure S15. IR spectrum of compound **3h**.

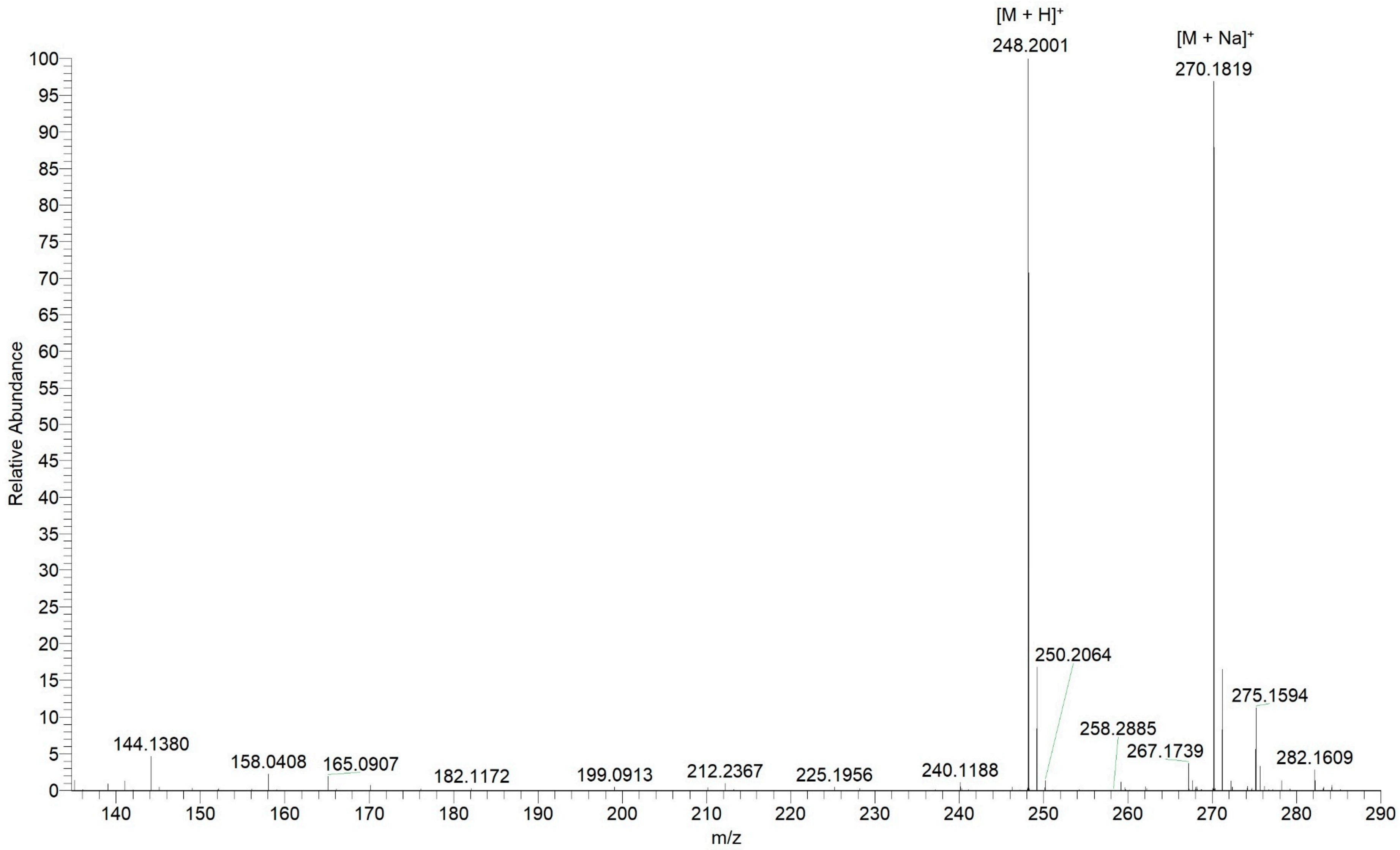

Figure S16. ESI-HRMS of compound **3a**.

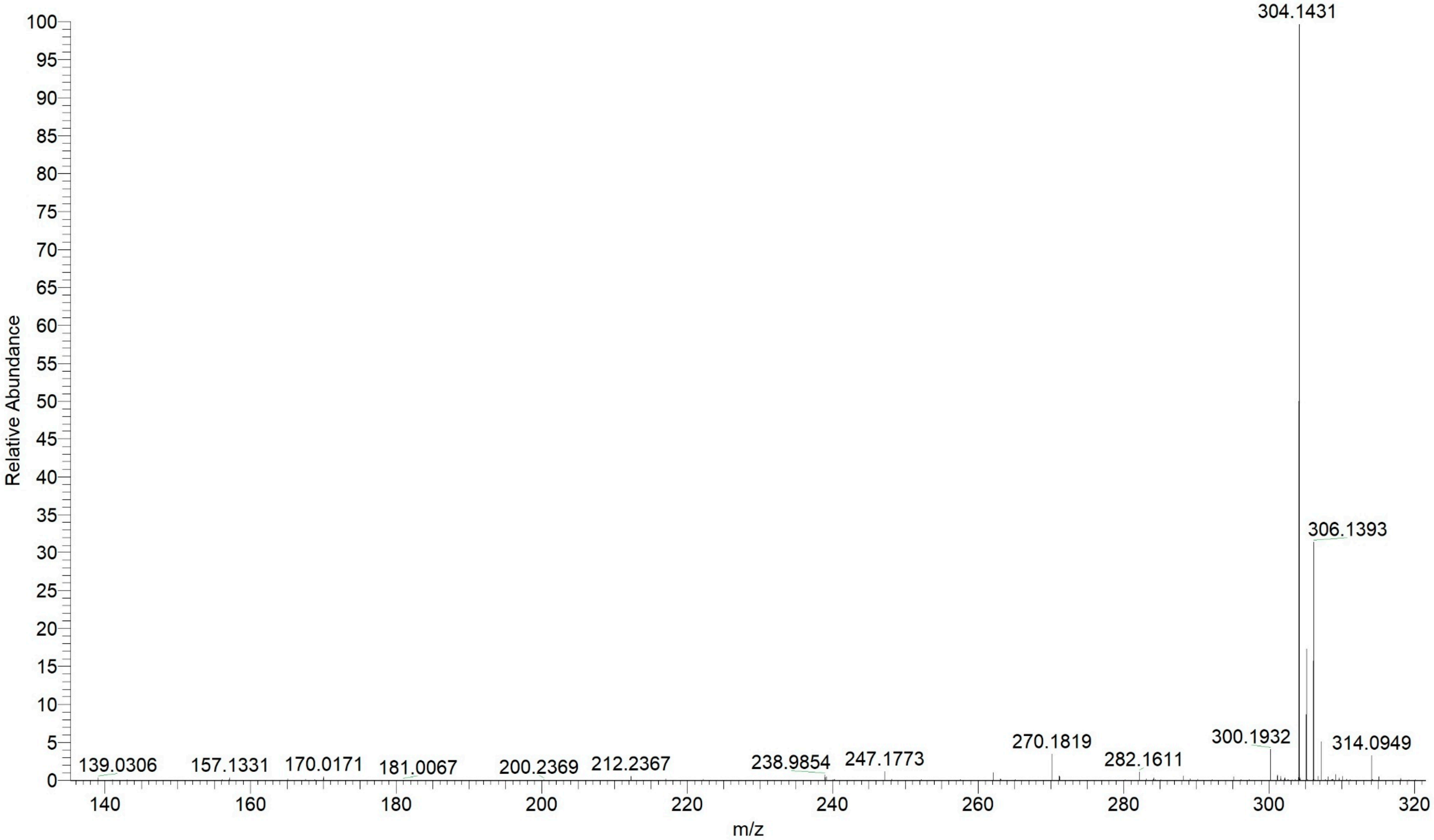

Figure S17. ESI-HRMS of compound **3b**.

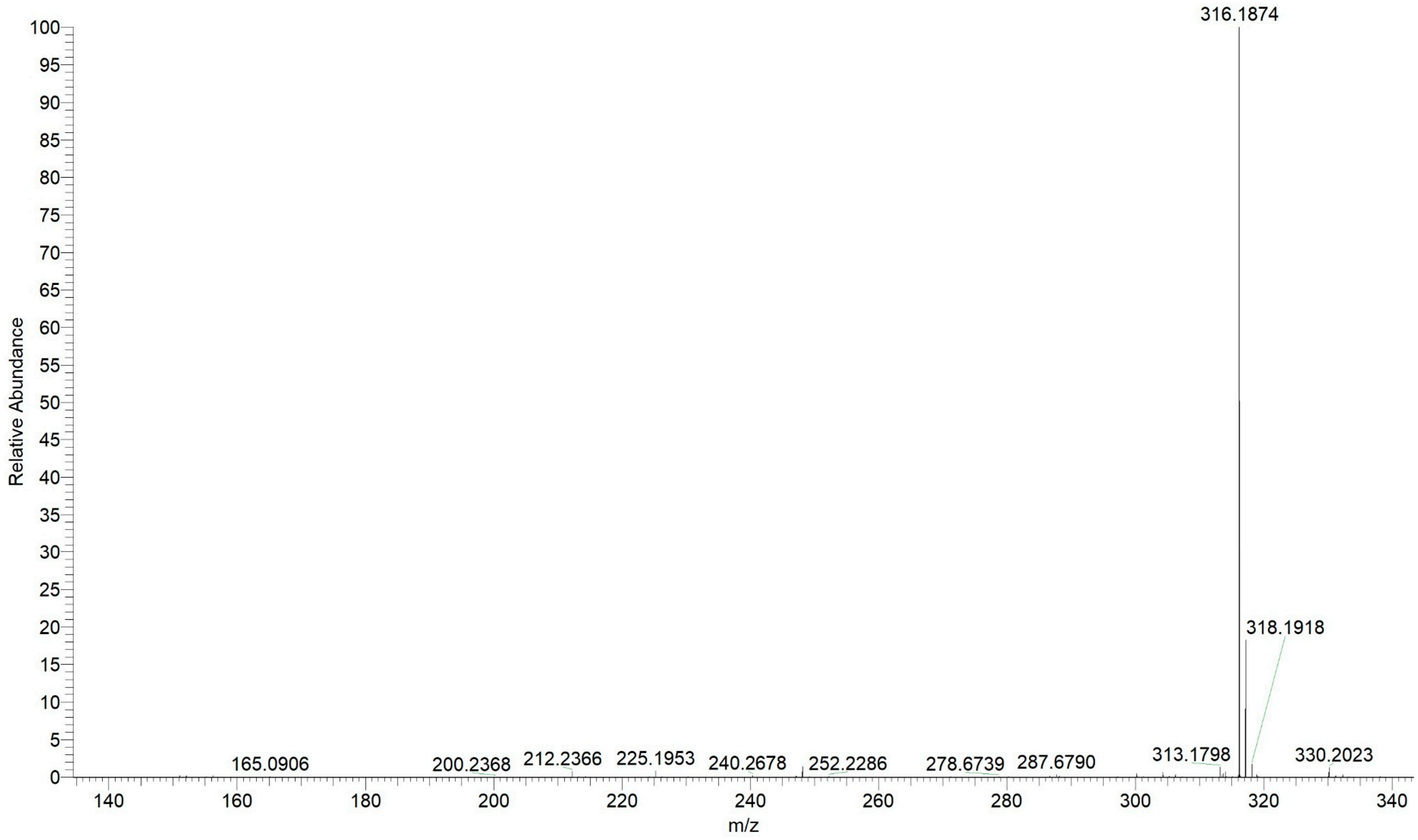

Figure S18. ESI-HRMS of compound **3f**.

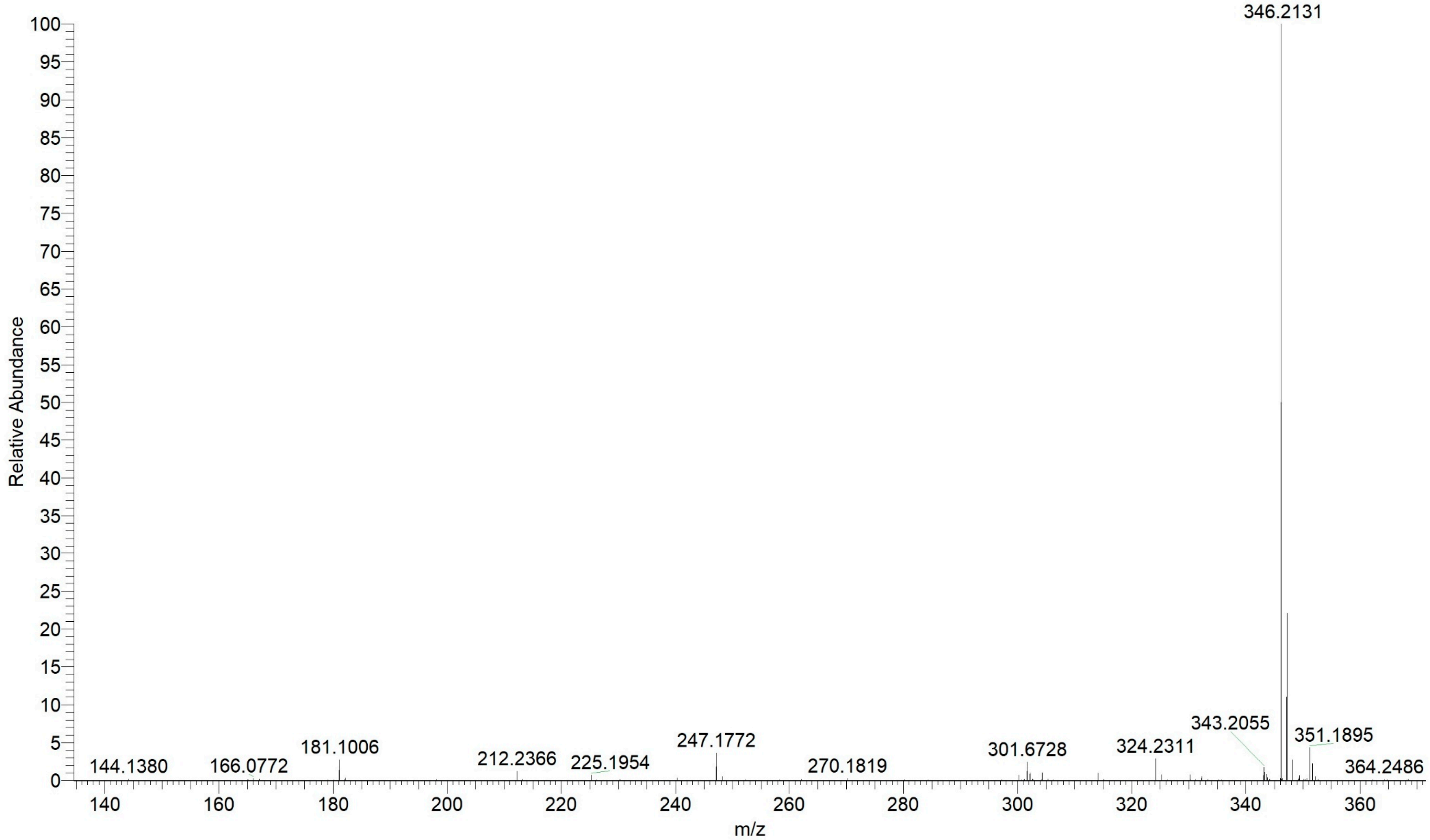

Figure S19. ESI-HRMS of compound **3g**.

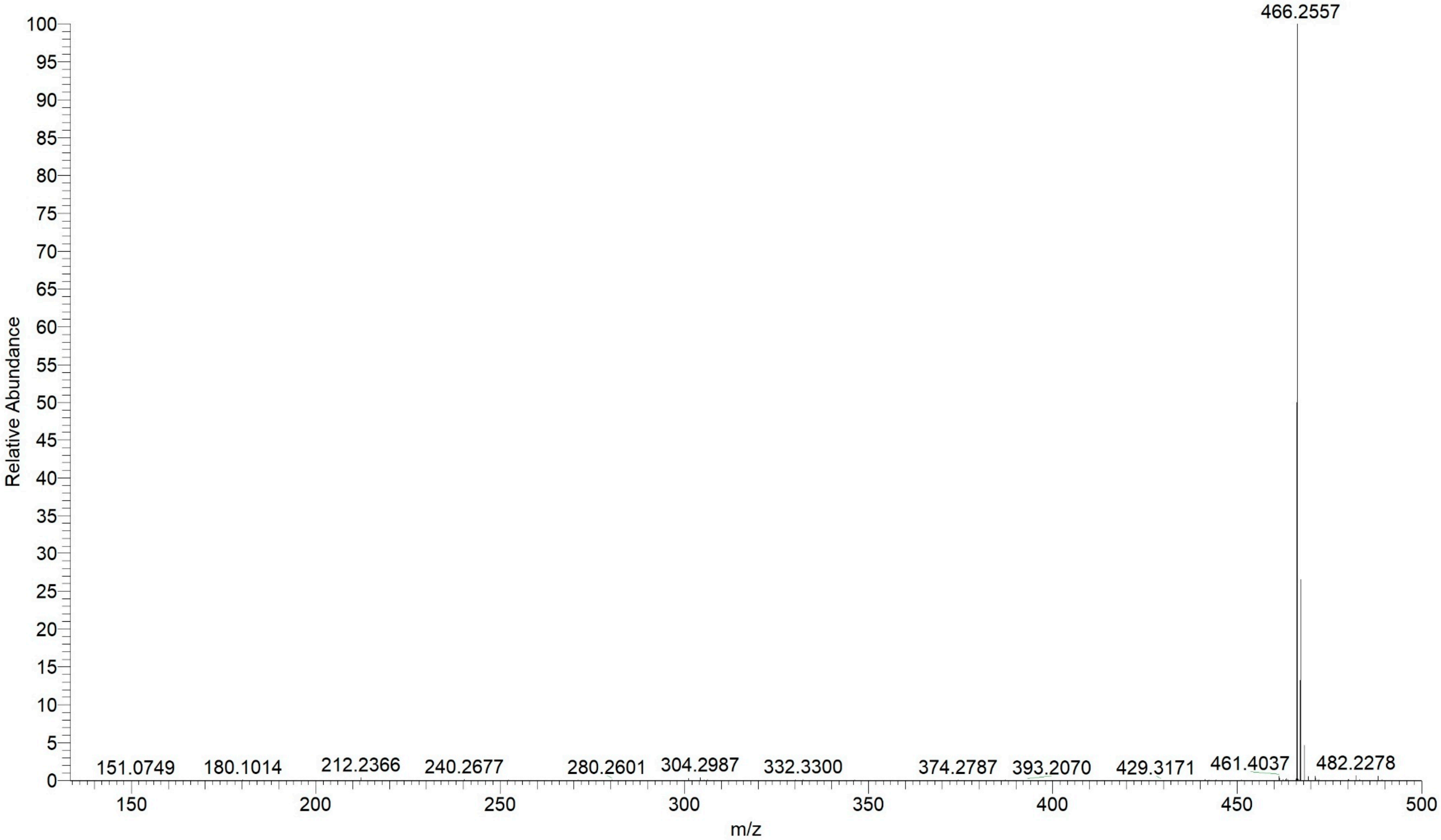

Figure S20. ESI-HRMS of compound **3h**.

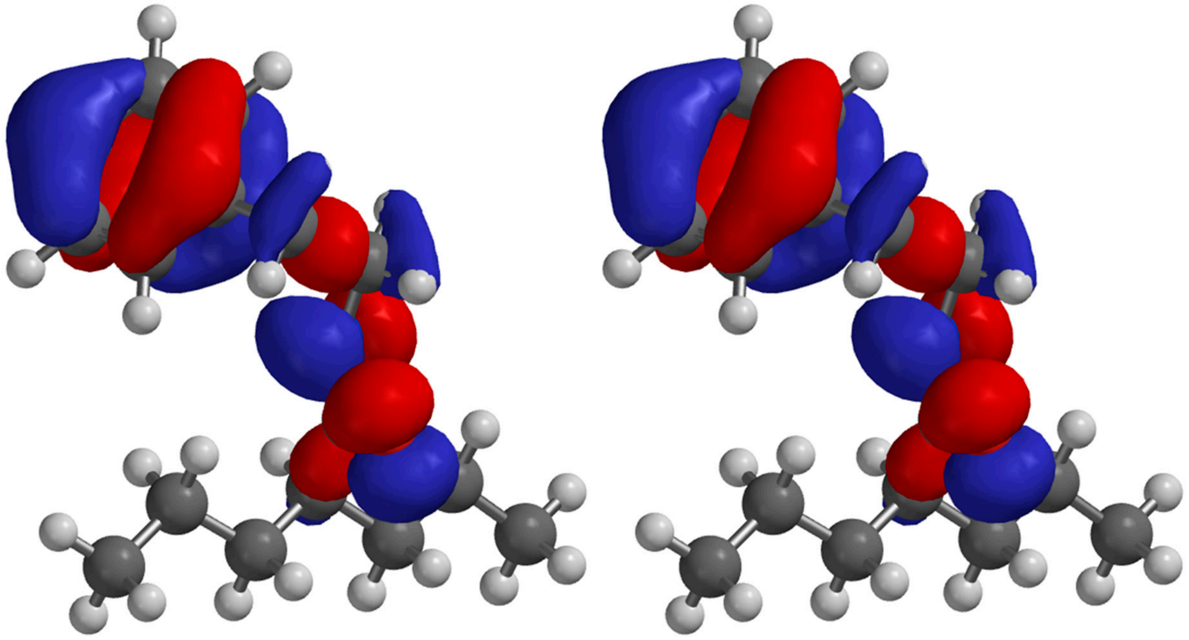

Figure S21. HOMO (left) and LUMO (right) of compound **3a**.

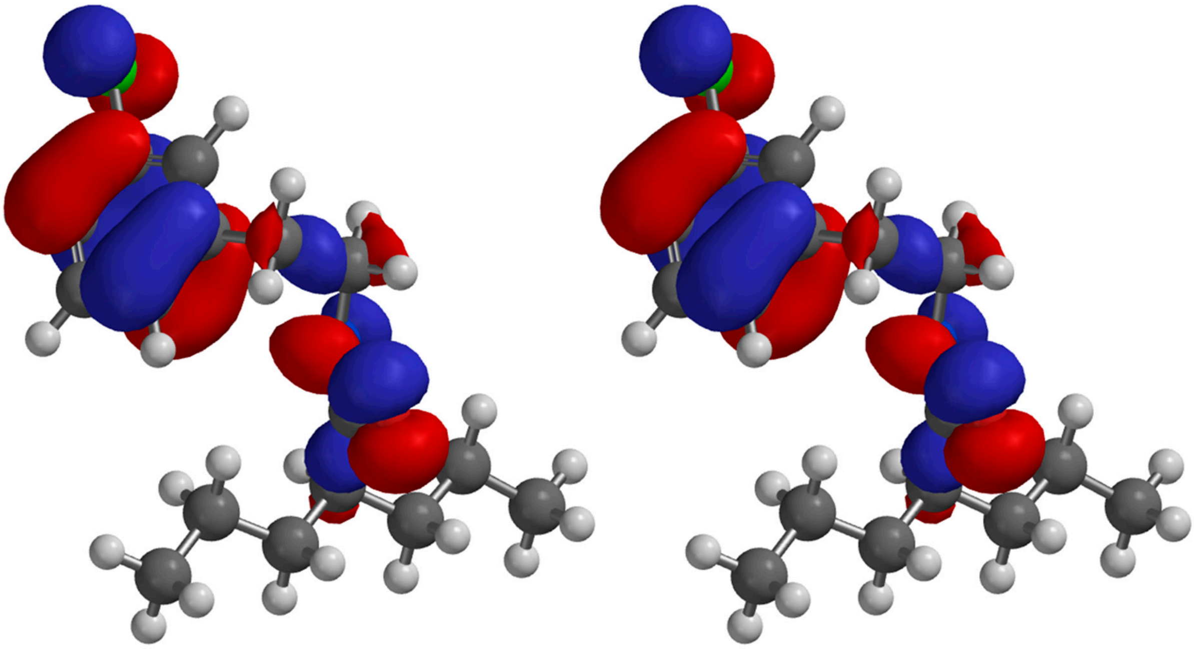

Figure S22. HOMO (left) and LUMO (right) of compound **3b**.

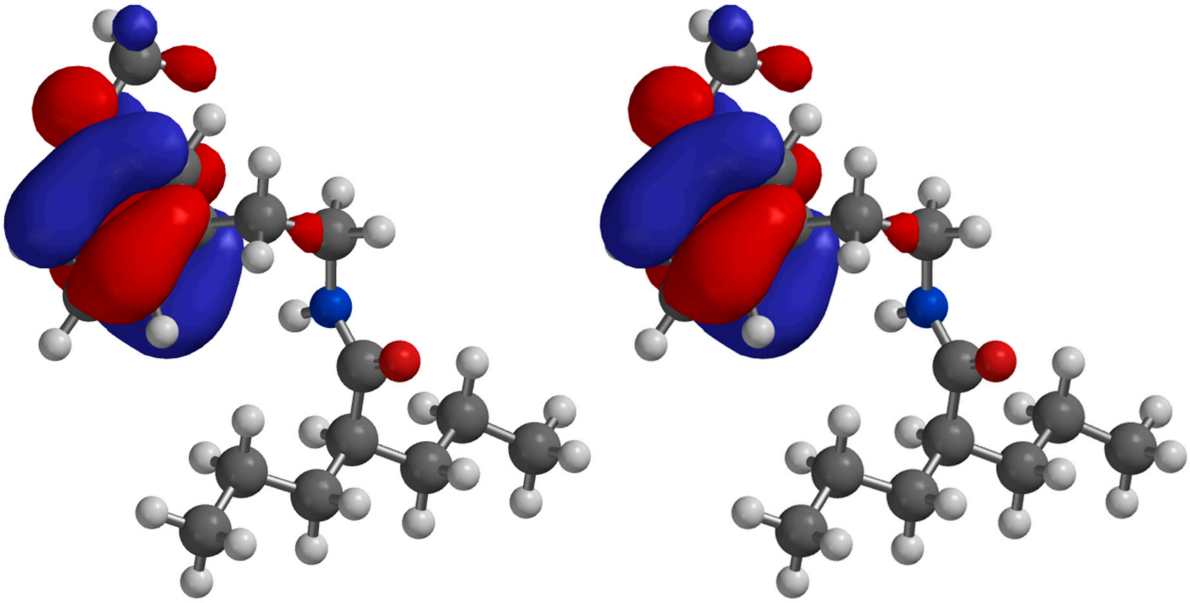

Figure S23. HOMO (left) and LUMO (right) of compound **3c**.

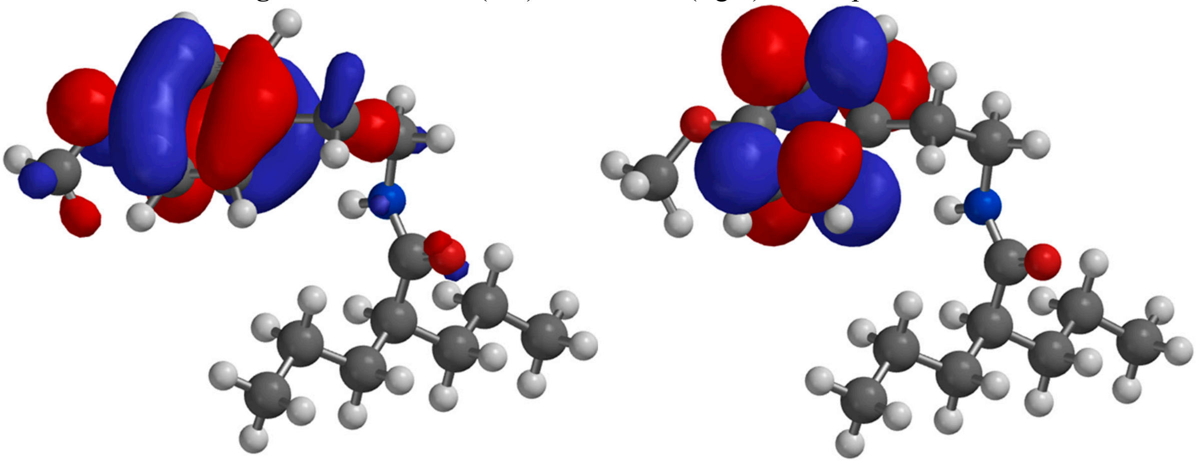

Figure S24. HOMO (left) and LUMO (right) of compound **3d**.

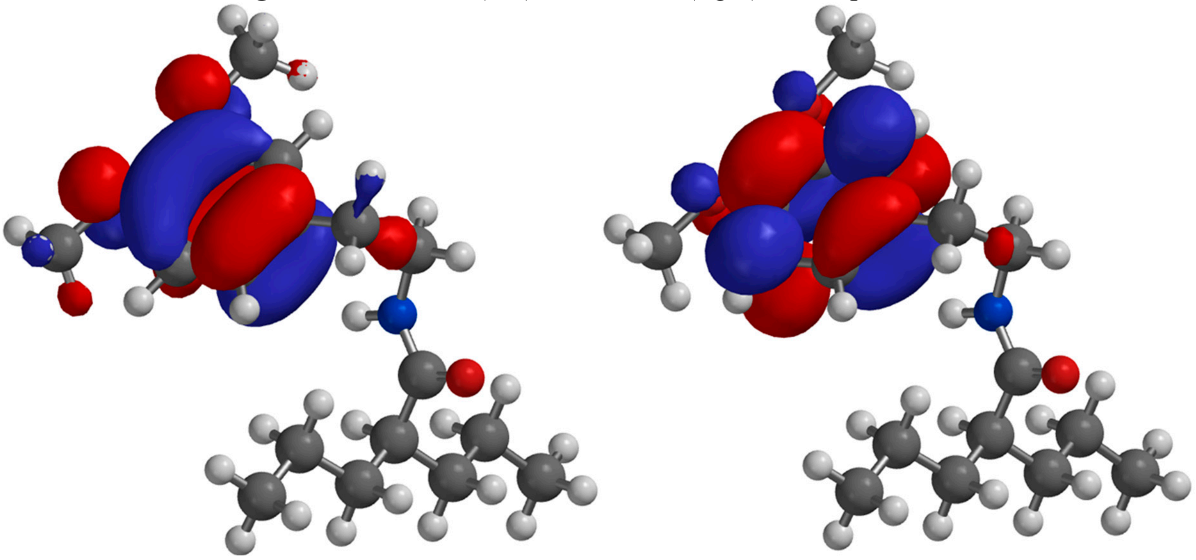

Figure S25. HOMO (left) and LUMO (right) of compound **3e**.

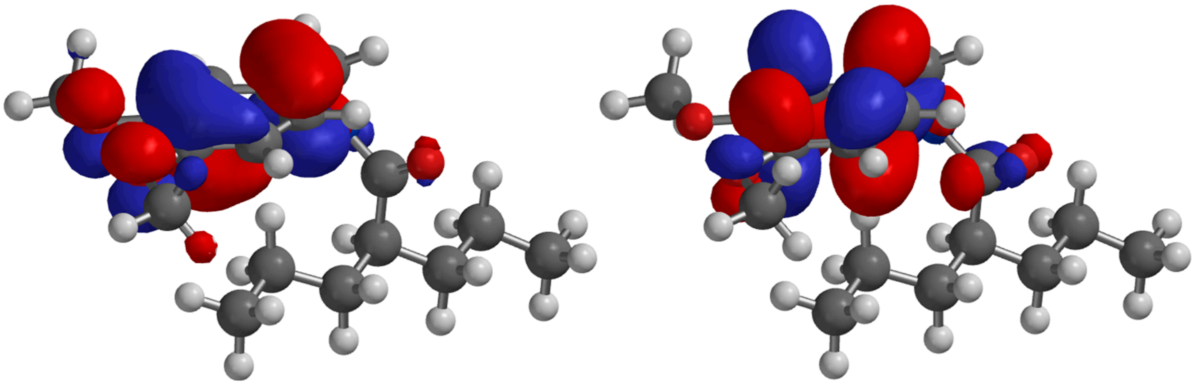

Figure S26. HOMO (left) and LUMO (right) of compound **3f**.

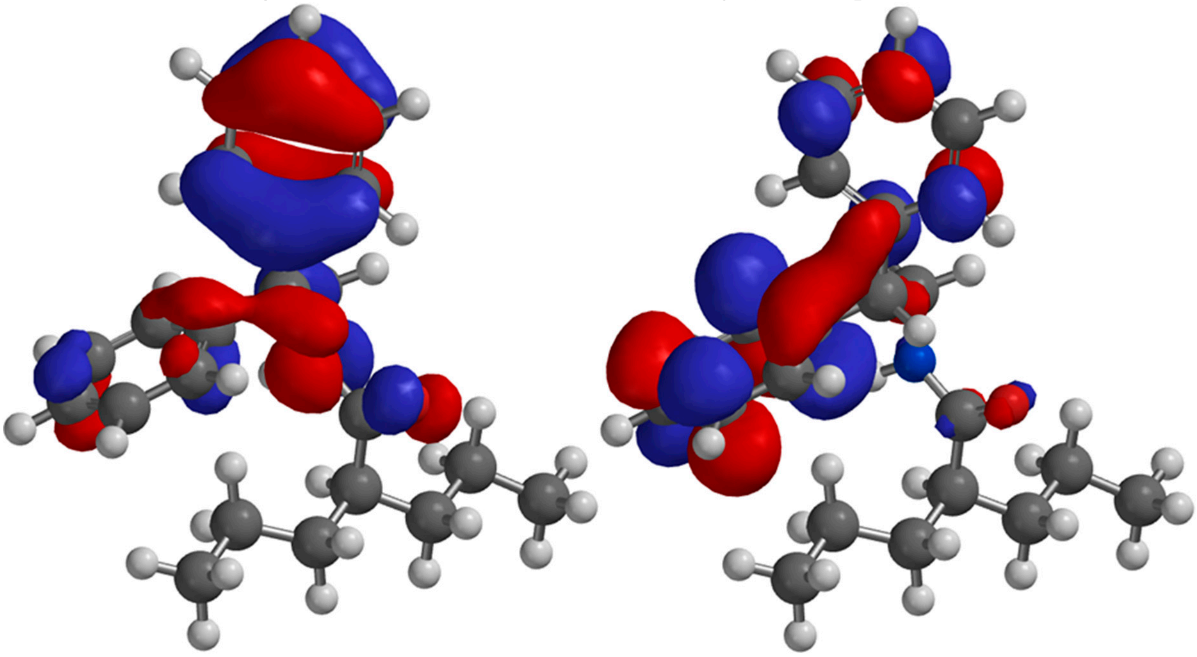

Figure S27. HOMO (left) and LUMO (right) of compound **3g**.

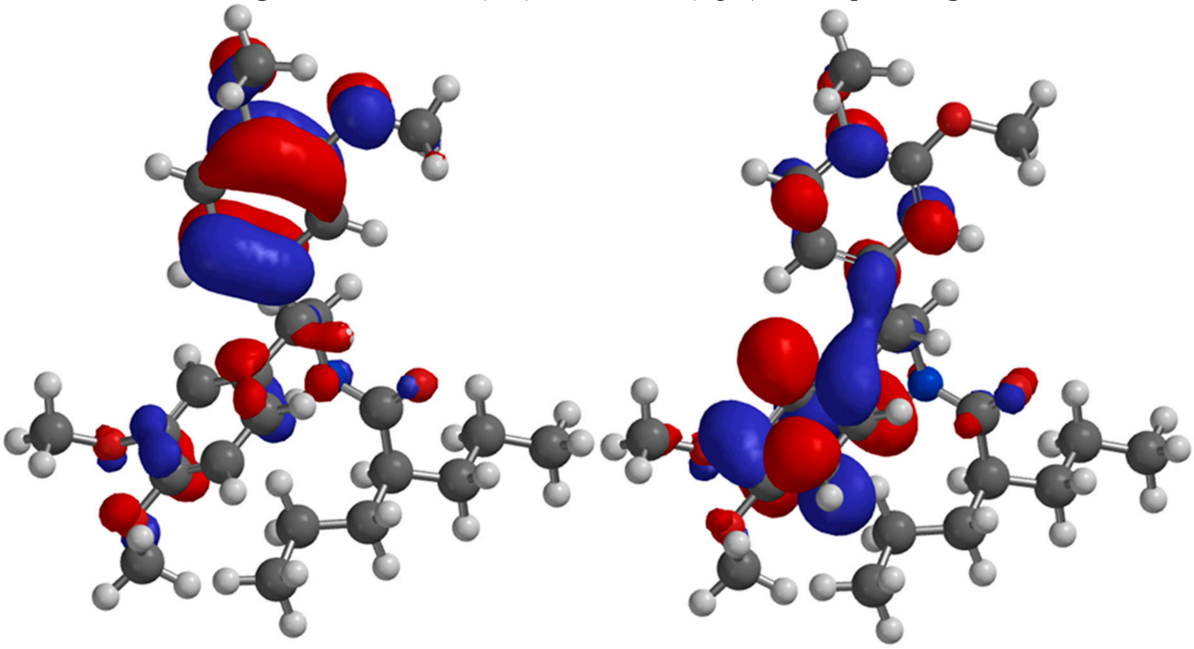

Figure S28. HOMO (left) and LUMO (right) of compound **3h**.

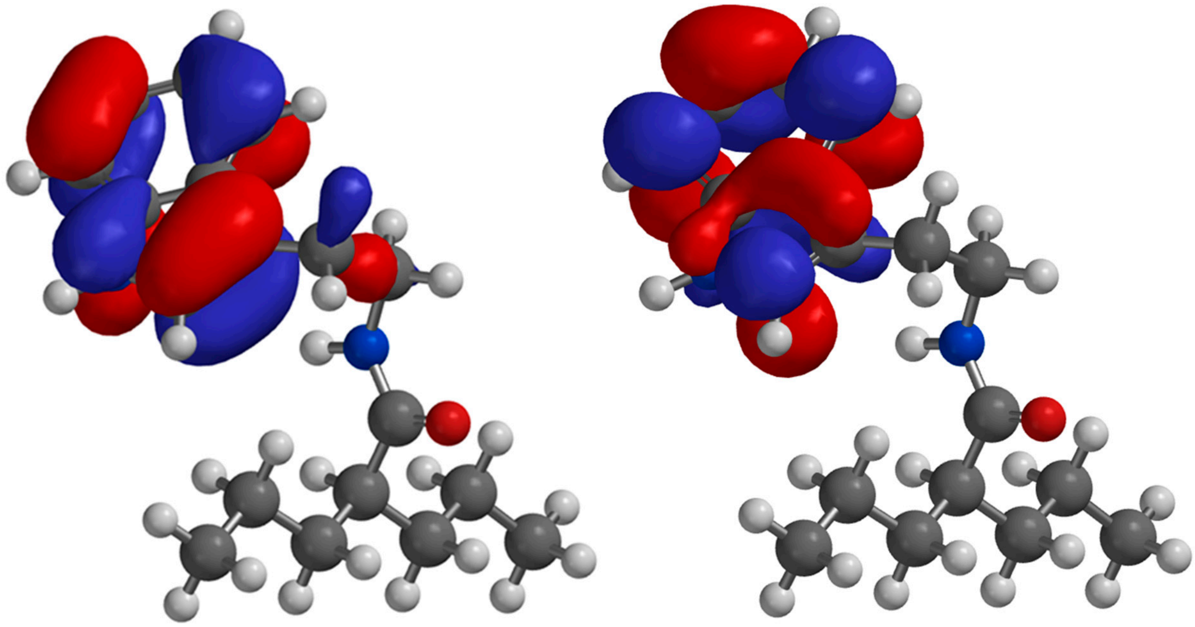

Figure S29. HOMO (left) and LUMO (right) of compound **3i**.

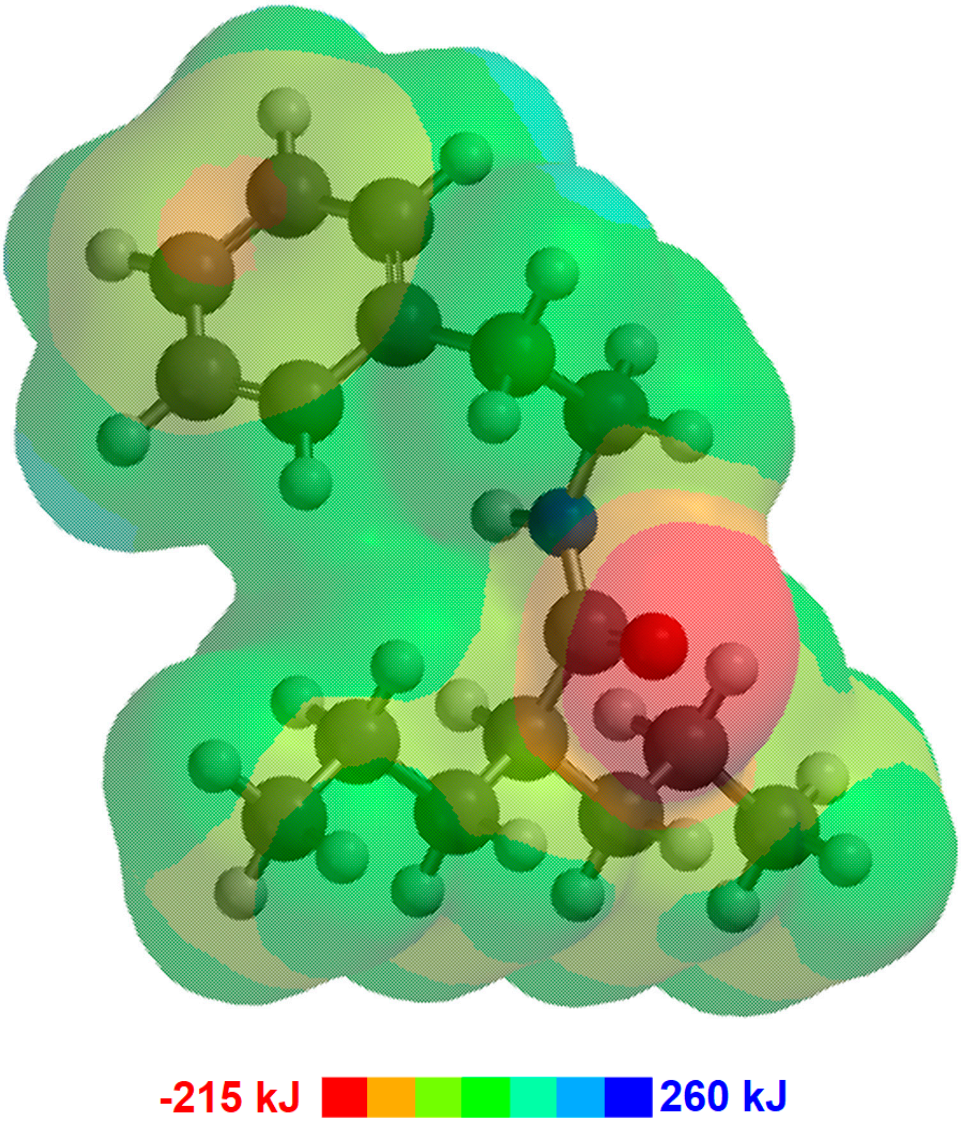

Figure S30. Electrostatic potential map of compound **3a**

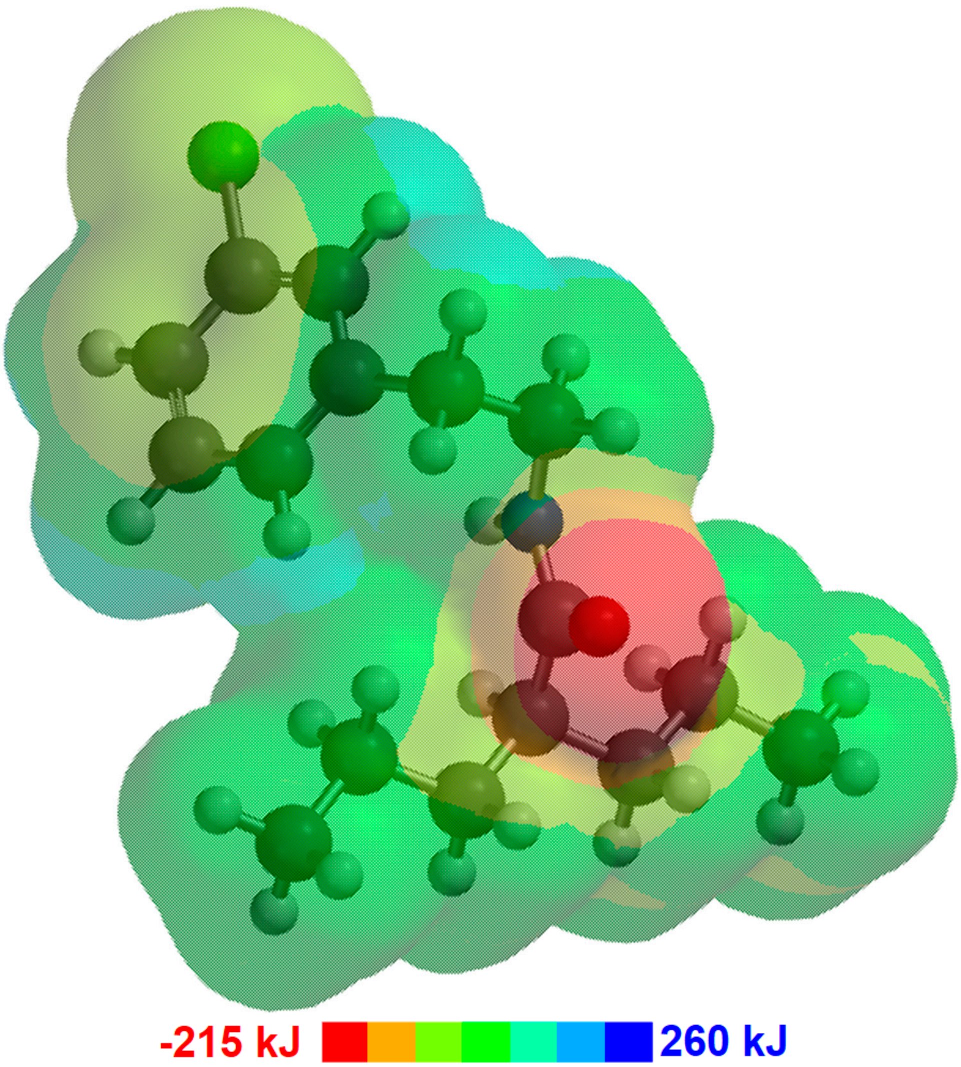

Figure S31. Electrostatic potential map of compound **3b**.

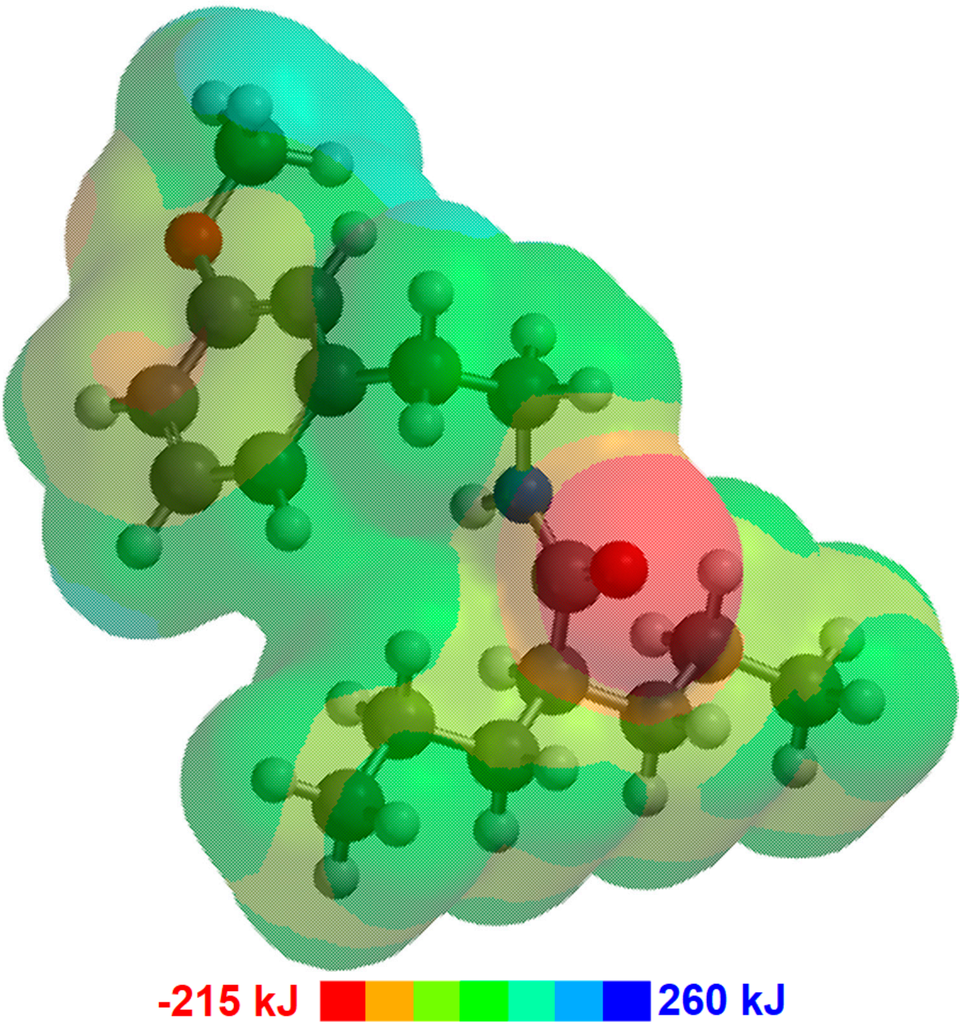

Figure S32. Electrostatic potential map of compound **3c**.

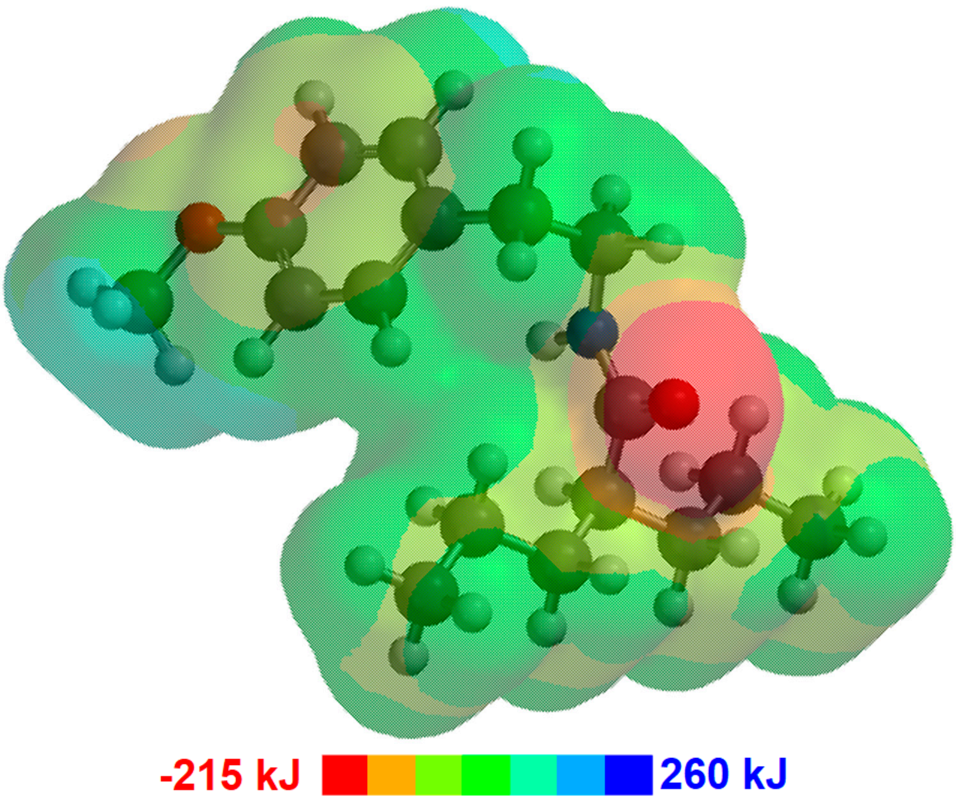

Figure S33. Electrostatic potential map of compound **3d**.

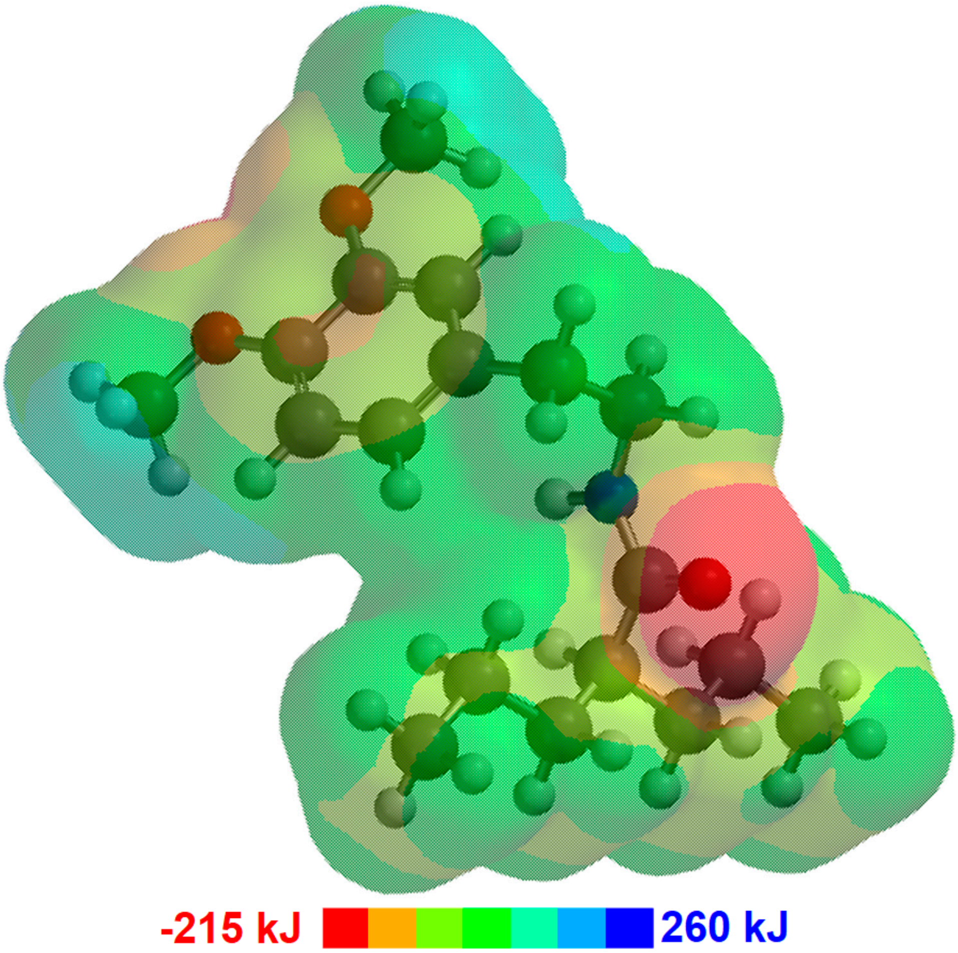

Figure S34. Electrostatic potential map of compound **3e**.

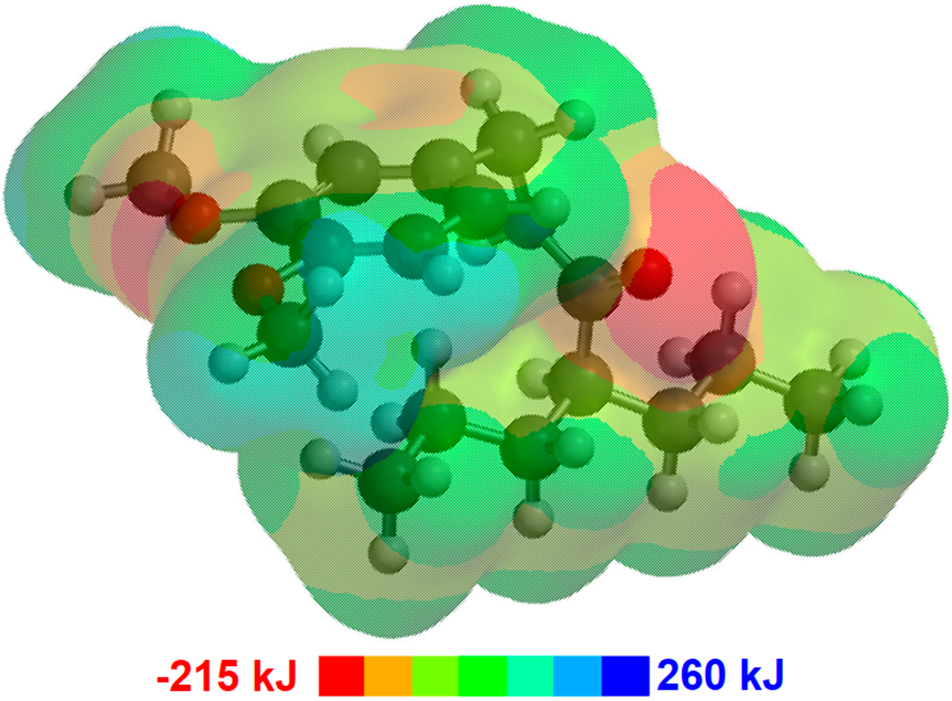

Figure S35. Electrostatic potential map of compound **3f**.

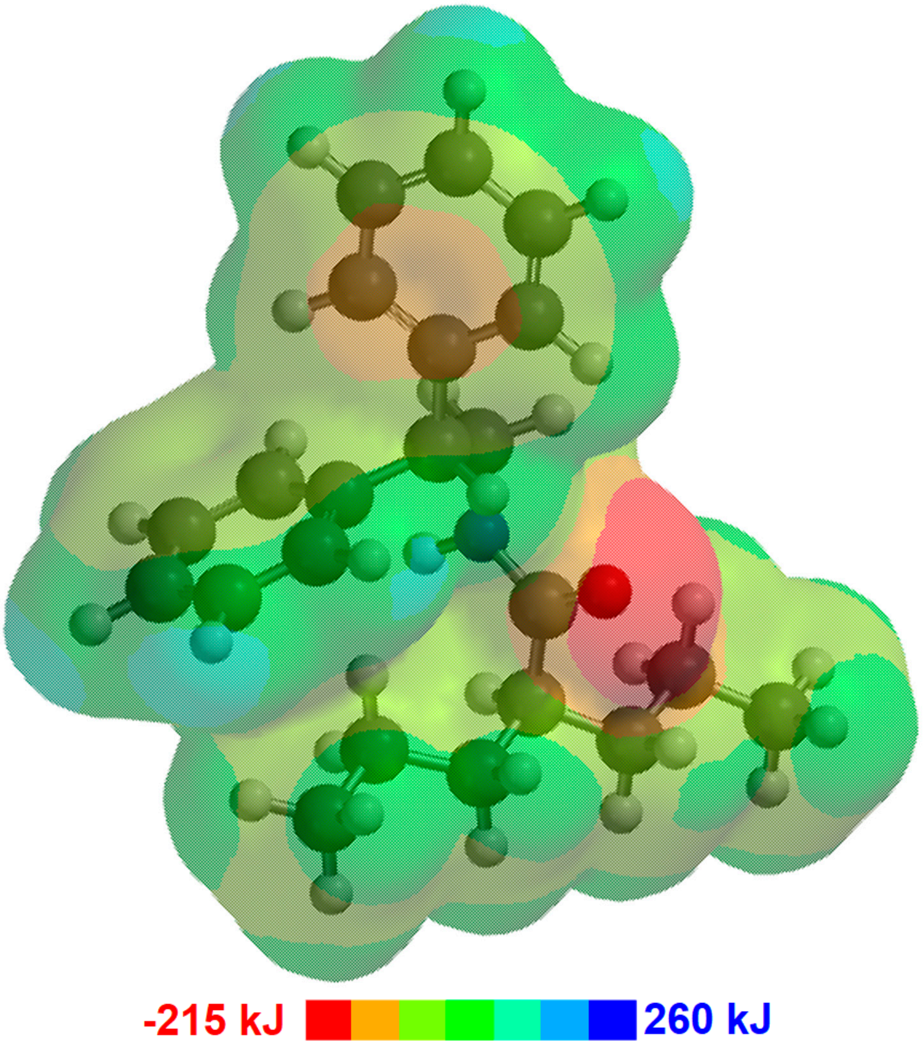

Figure S36. Electrostatic potential map of compound **3g**.

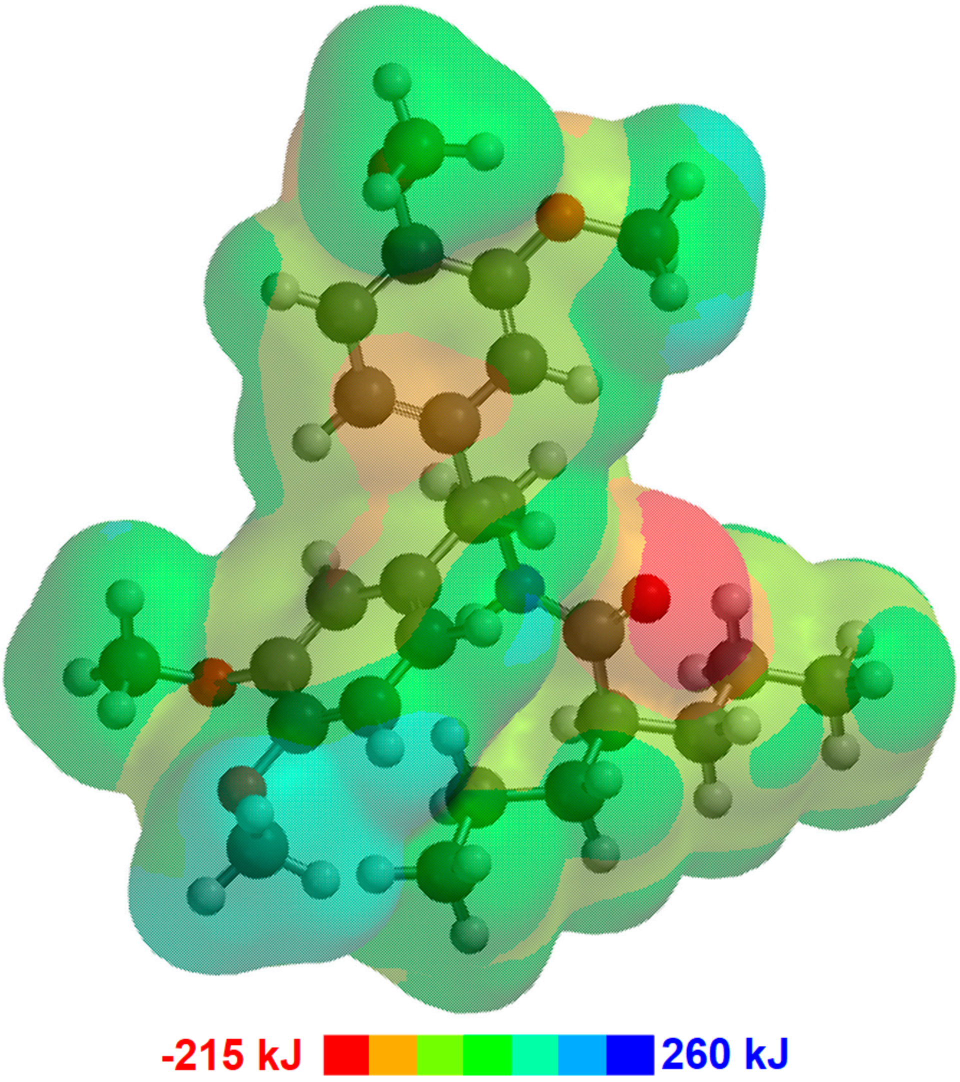

Figure S37. Electrostatic potential map of compound **3h**.

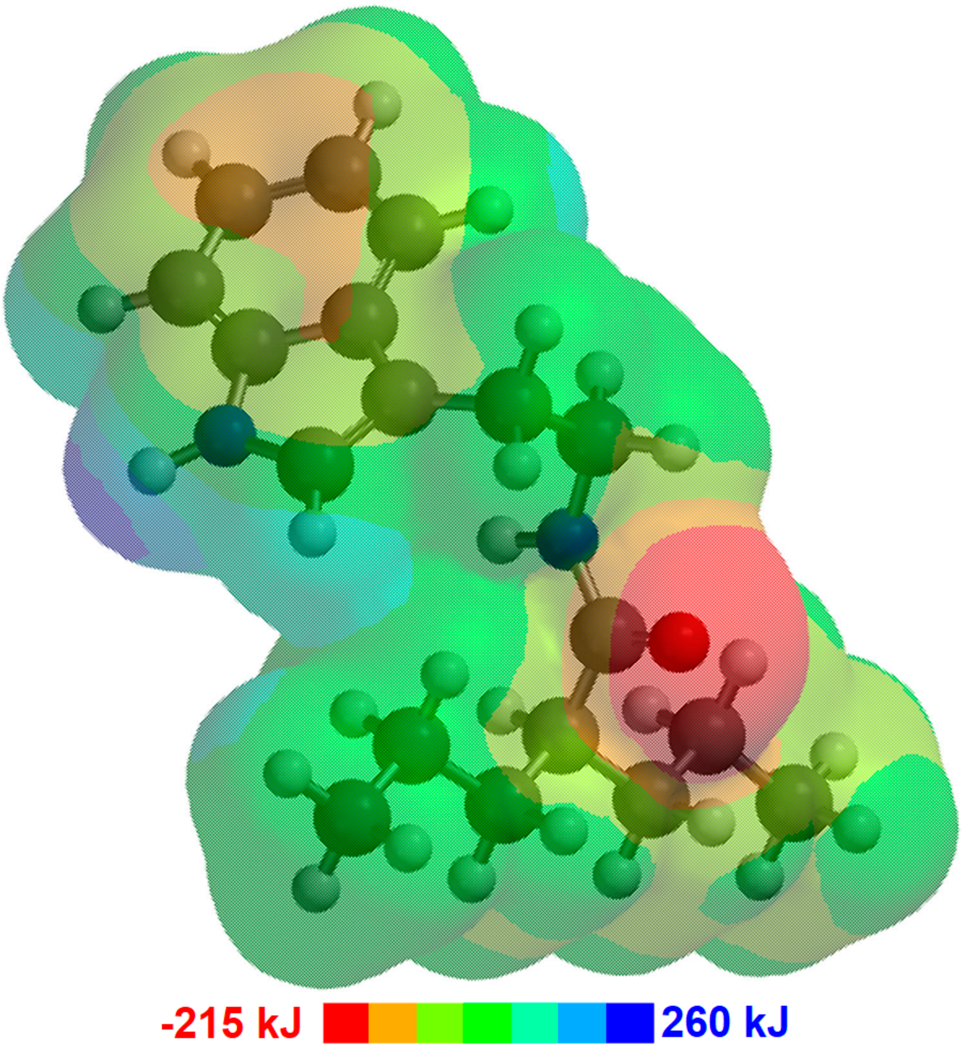

Figure S38. Electrostatic potential map of compound **3i**.

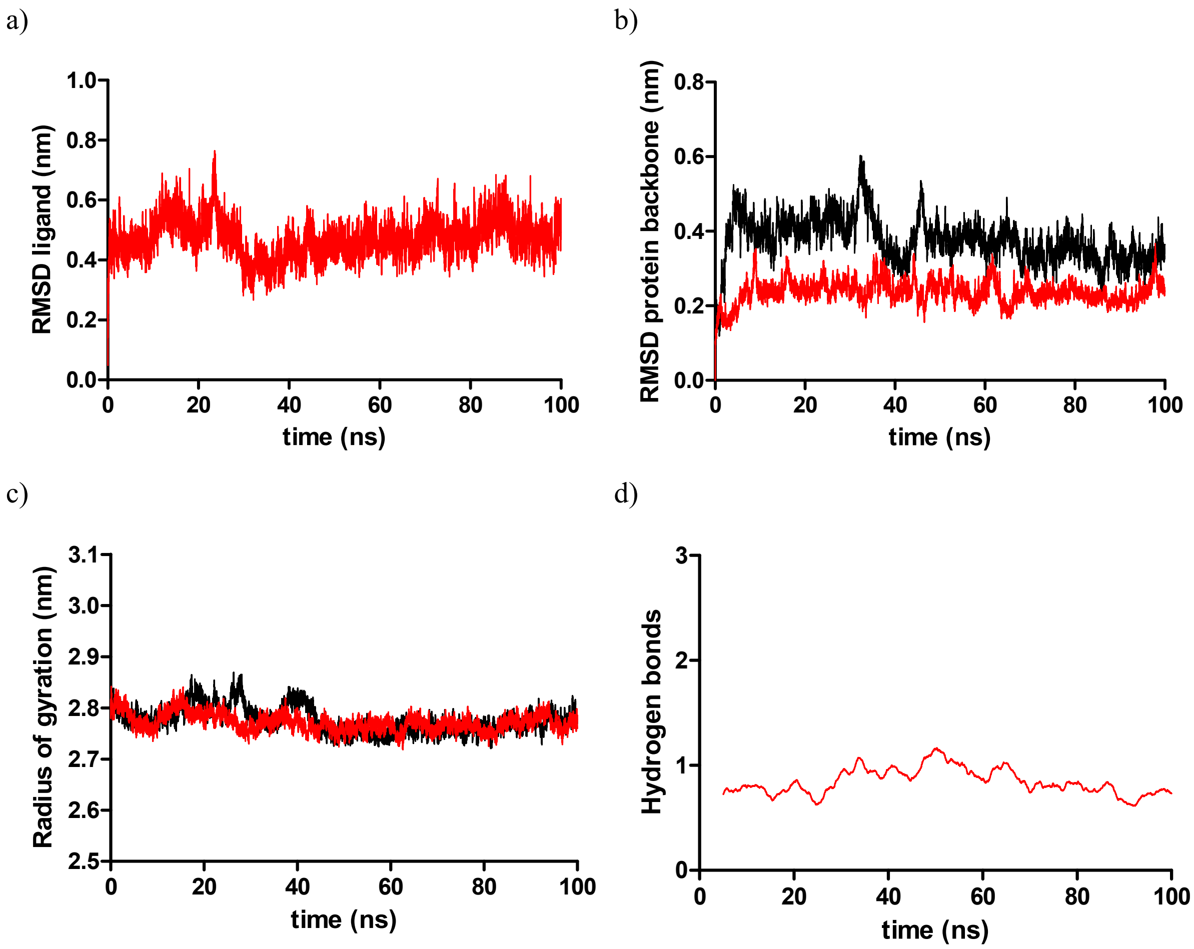

Figure S39. Analysis of the evolution in time of the complex of the compound **3a** docked in site 3 of HSA. a) RMSD of heavy atoms of the ligand, b) RMSD of HSA backbone apo (black) and in complex with the ligand (red), c) RG of HSA backbone apo (black) and in complex with the ligand (red), d) hydrogen bonds between the ligand and HSA (5 ns moving average).

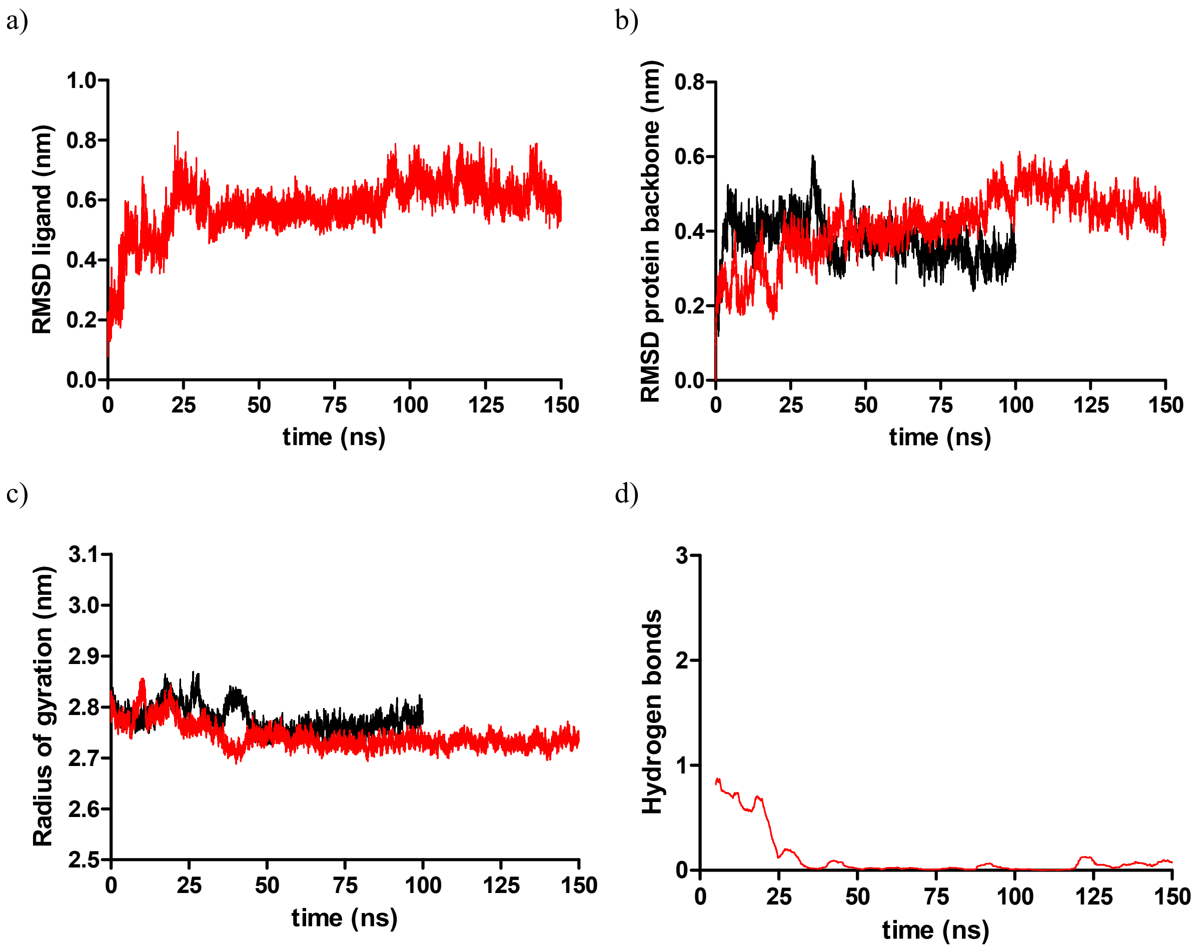

Figure S40. Analysis of the evolution in time of the complex of the compound **3b** docked in site 3 of HSA. a) RMSD of heavy atoms of the ligand, b) RMSD of HSA backbone apo (black) and in complex with the ligand (red), c) RG of HSA backbone apo (black) and in complex with the ligand (red), d) hydrogen bonds between the ligand and HSA (5 ns moving average).

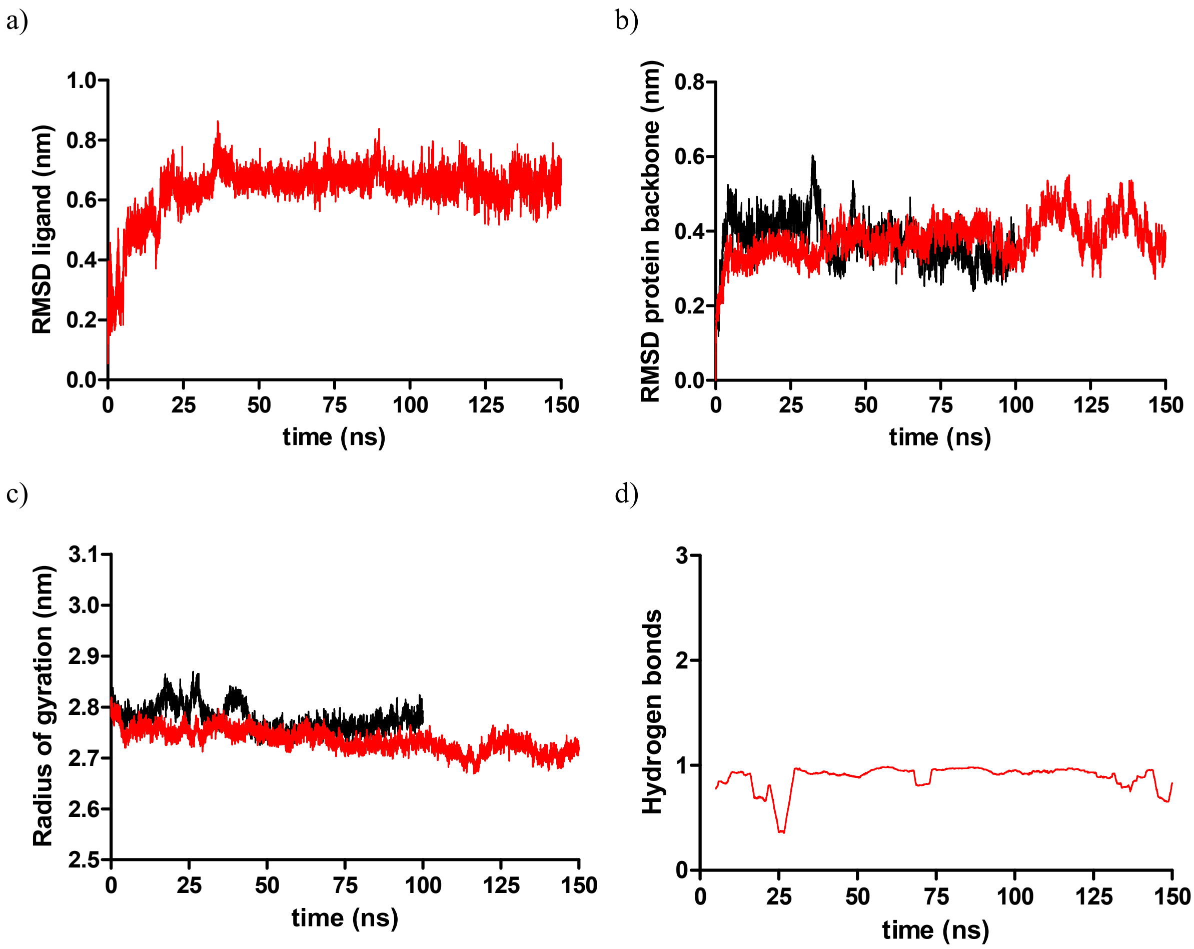

Figure S41. Analysis of the evolution in time of the complex of the compound **3c** docked in site 3 of HSA. a) RMSD of heavy atoms of the ligand, b) RMSD of HSA backbone apo (black) and in complex with the ligand (red), c) RG of HSA backbone apo (black) and in complex with the ligand (red), d) hydrogen bonds between the ligand and HSA (5 ns moving average).

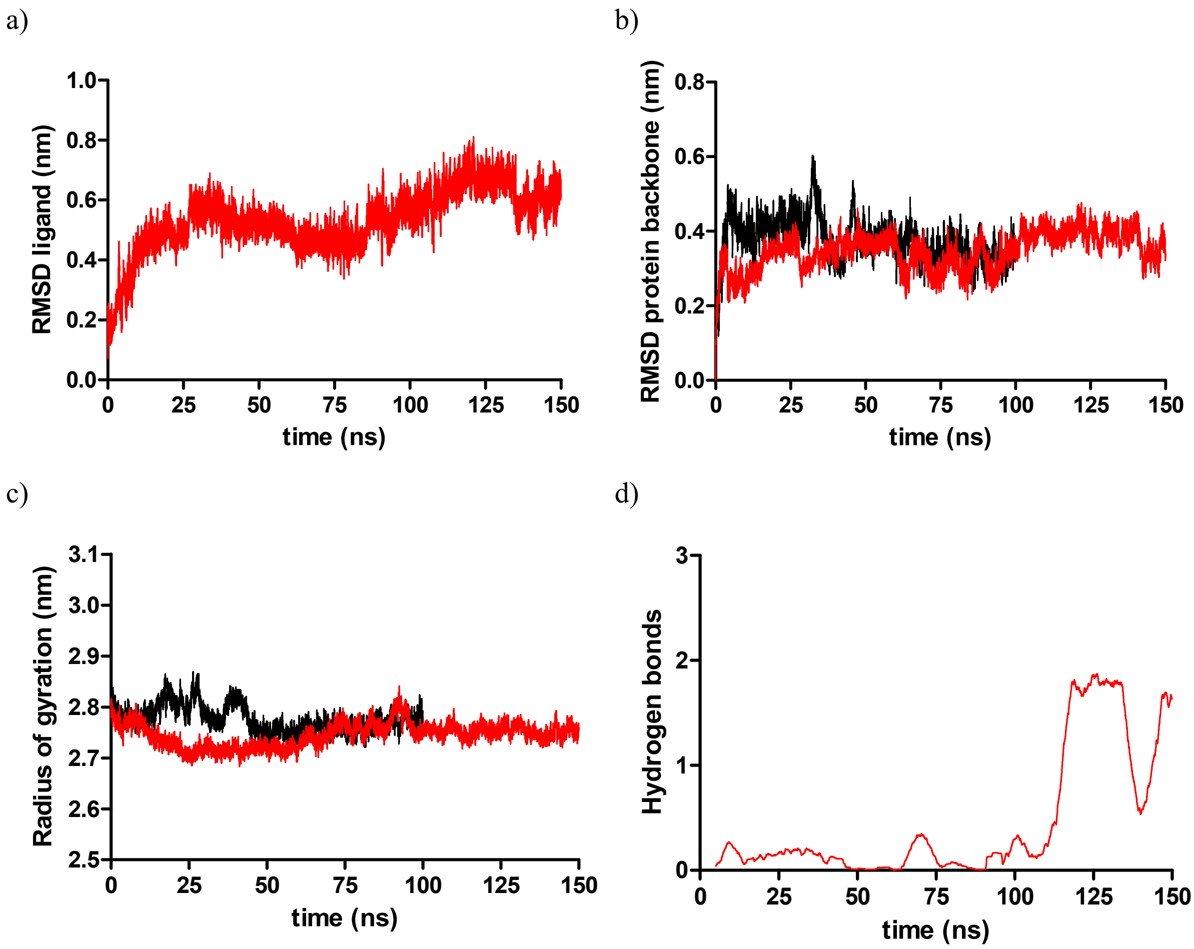

Figure S42. Analysis of the evolution in time of the complex of the compound **3d** docked in site 3 of HSA. a) RMSD of heavy atoms of the ligand, b) RMSD of HSA backbone apo (black) and in complex with the ligand (red), c) RG of HSA backbone apo (black) and in complex with the ligand (red), d) hydrogen bonds between the ligand and HSA (5 ns moving average).

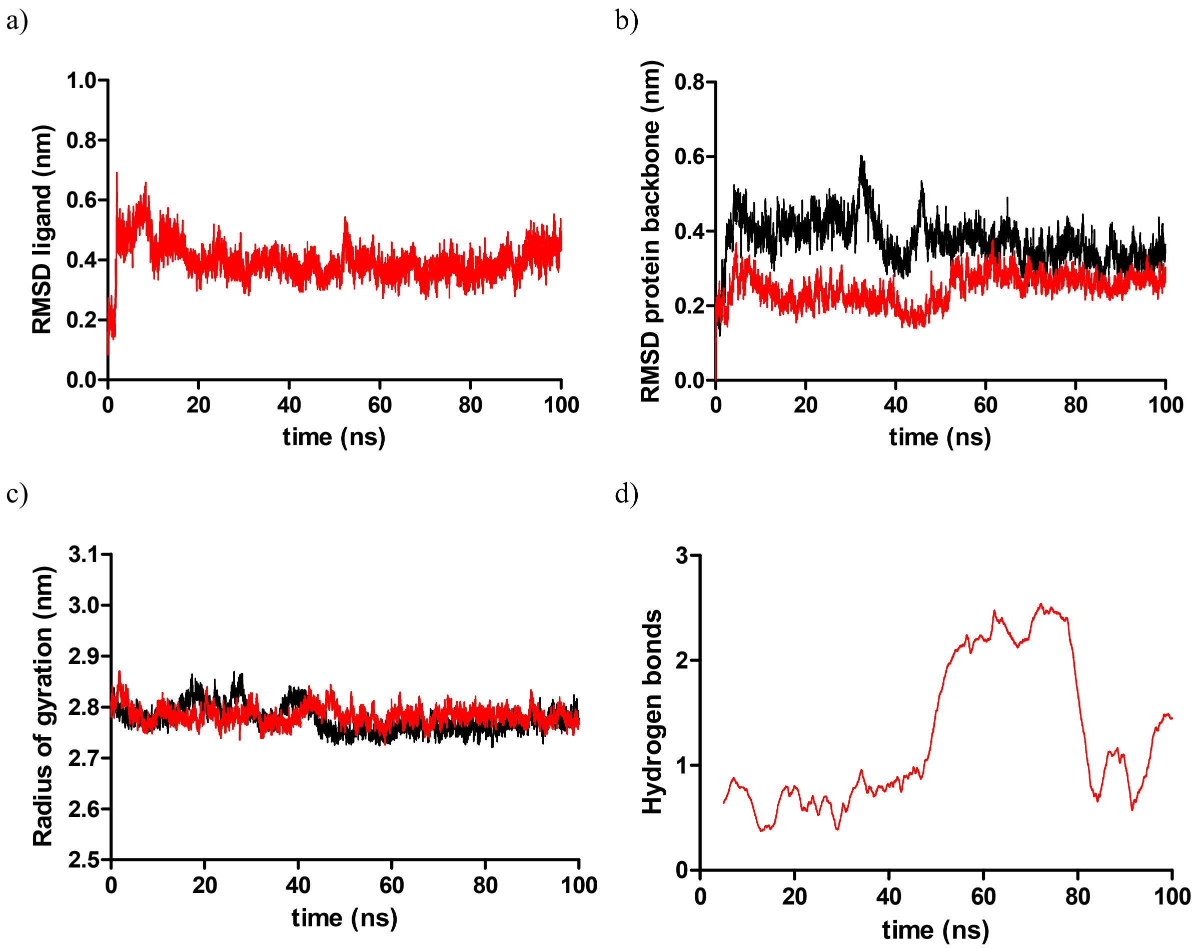

Figure S43. Analysis of the evolution in time of the complex of the compound **3e** docked in site 3 of HSA. a) RMSD of heavy atoms of the ligand, b) RMSD of HSA backbone apo (black) and in complex with the ligand (red), c) RG of HSA backbone apo (black) and in complex with the ligand (red), d) hydrogen bonds between the ligand and HSA (5 ns moving average).

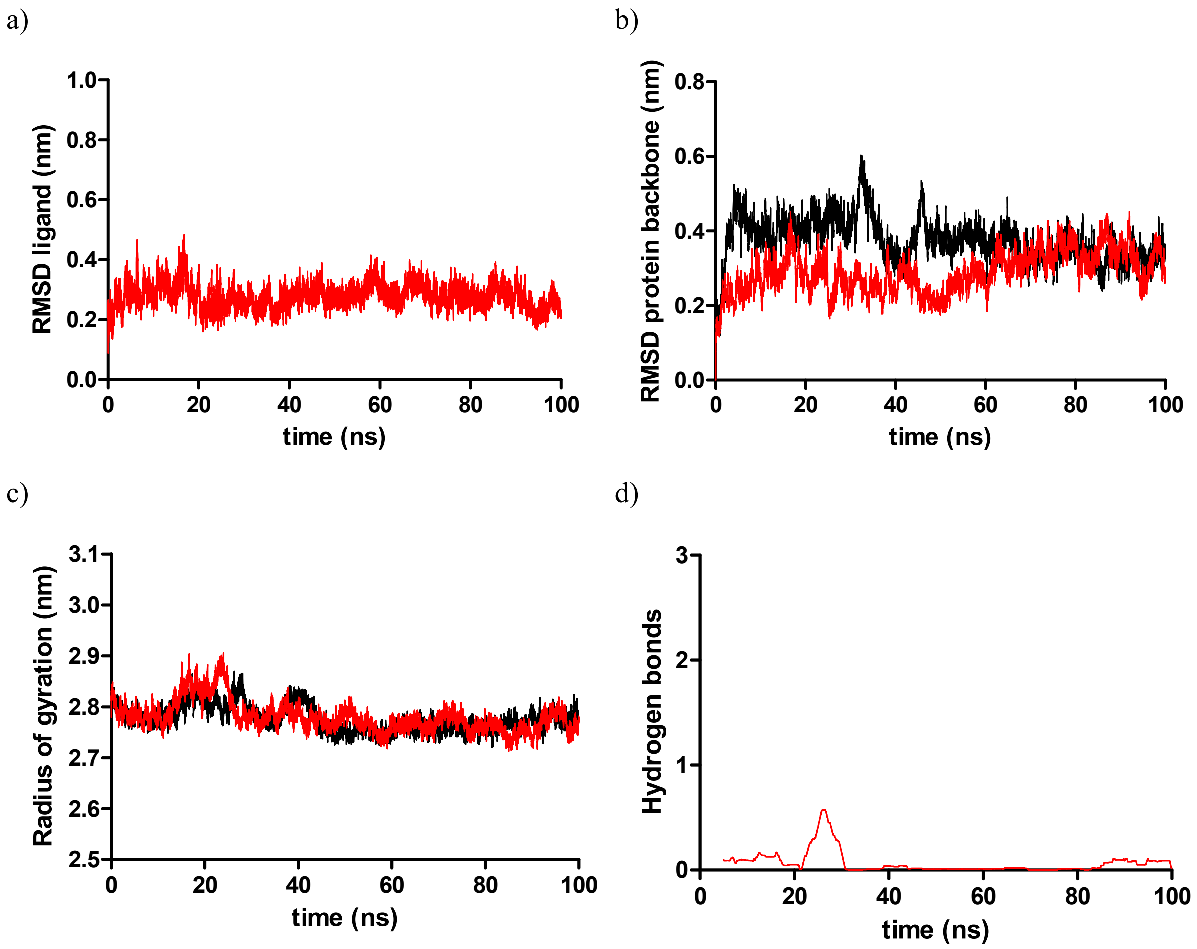

Figure S44. Analysis of the evolution in time of the complex of the compound **3f** docked in site 3 of HSA. a) RMSD of heavy atoms of the ligand, b) RMSD of HSA backbone apo (black) and in complex with the ligand (red), c) RG of HSA backbone apo (black) and in complex with the ligand (red), d) hydrogen bonds between the ligand and HSA (5 ns moving average).

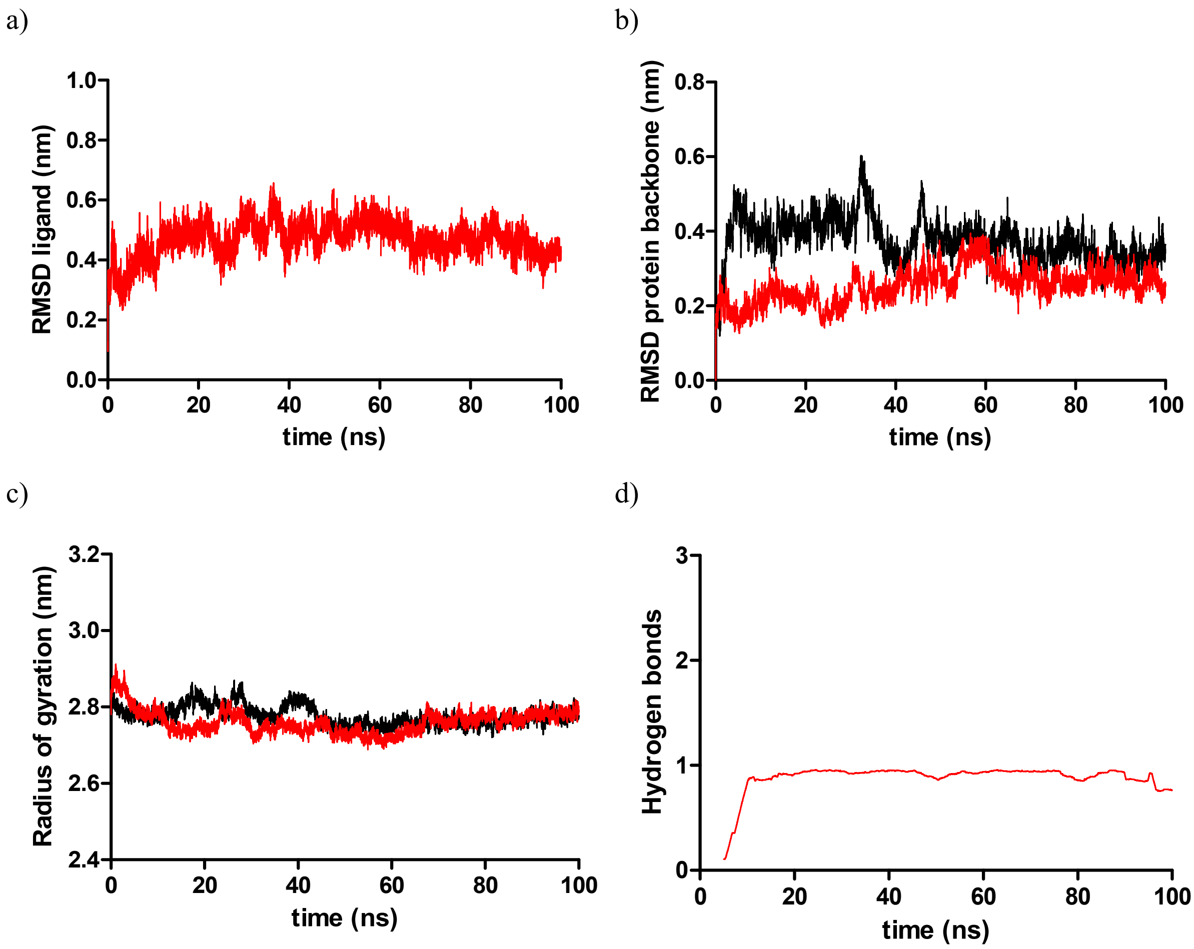

Figure S45. Analysis of the evolution in time of the complex of the compound **3g** docked in site 3 of HSA. a) RMSD of heavy atoms of the ligand, b) RMSD of HSA backbone apo (black) and in complex with the ligand (red), c) RG of HSA backbone apo (black) and in complex with the ligand (red), d) hydrogen bonds between the ligand and HSA (5 ns moving average).

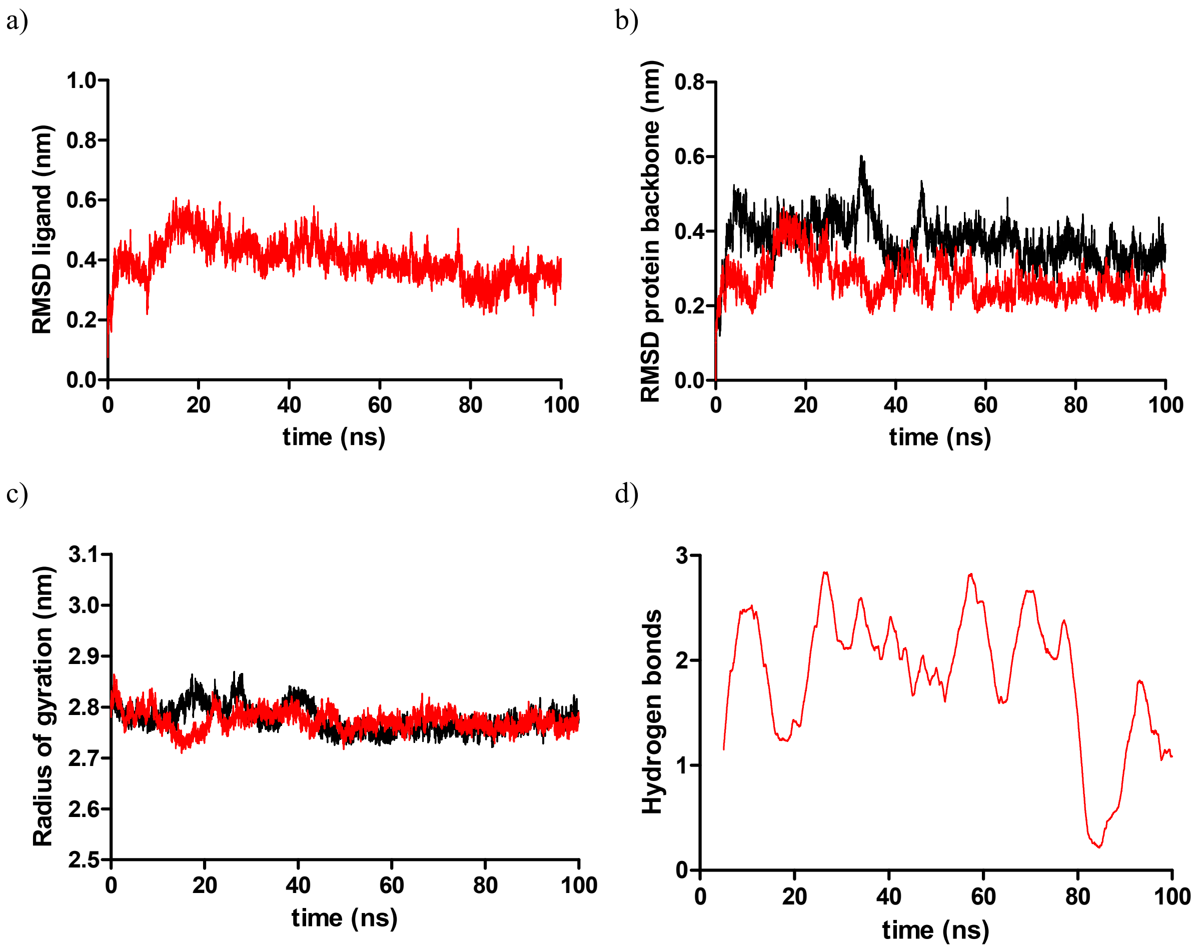

Figure S46. Analysis of the evolution in time of the complex of the compound **3h** docked in site 3 of HSA. a) RMSD of heavy atoms of the ligand, b) RMSD of HSA backbone apo (black) and in complex with the ligand (red), c) RG of HSA backbone apo (black) and in complex with the ligand (red), d) hydrogen bonds between the ligand and HSA (5 ns moving average).

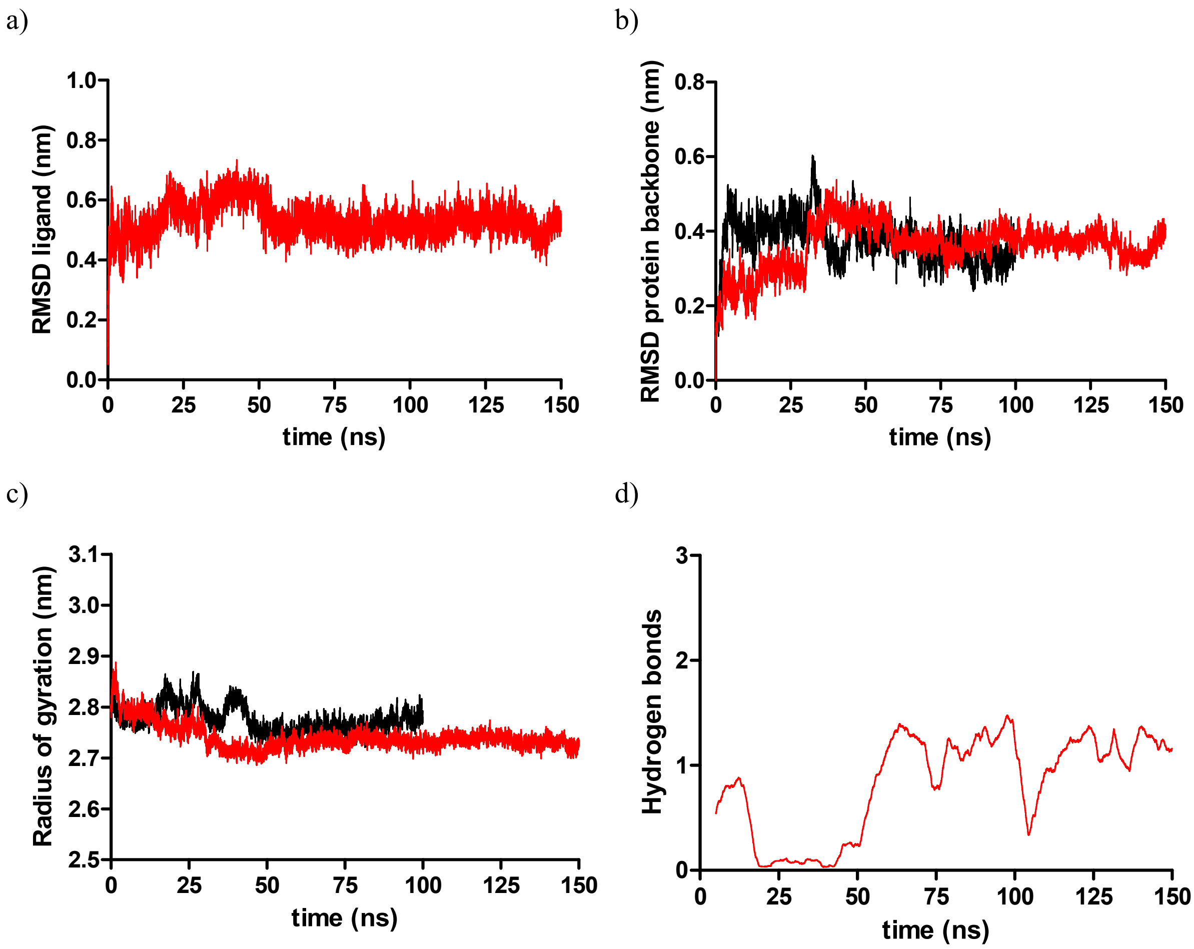

Figure S47. Analysis of the evolution in time of the complex of the compound **3i** docked in site 3 of HSA. a) RMSD of heavy atoms of the ligand, b) RMSD of HSA backbone apo (black) and in complex with the ligand (red), c) RG of HSA backbone apo (black) and in complex with the ligand (red), d) hydrogen bonds between the ligand and HSA (5 ns moving average).

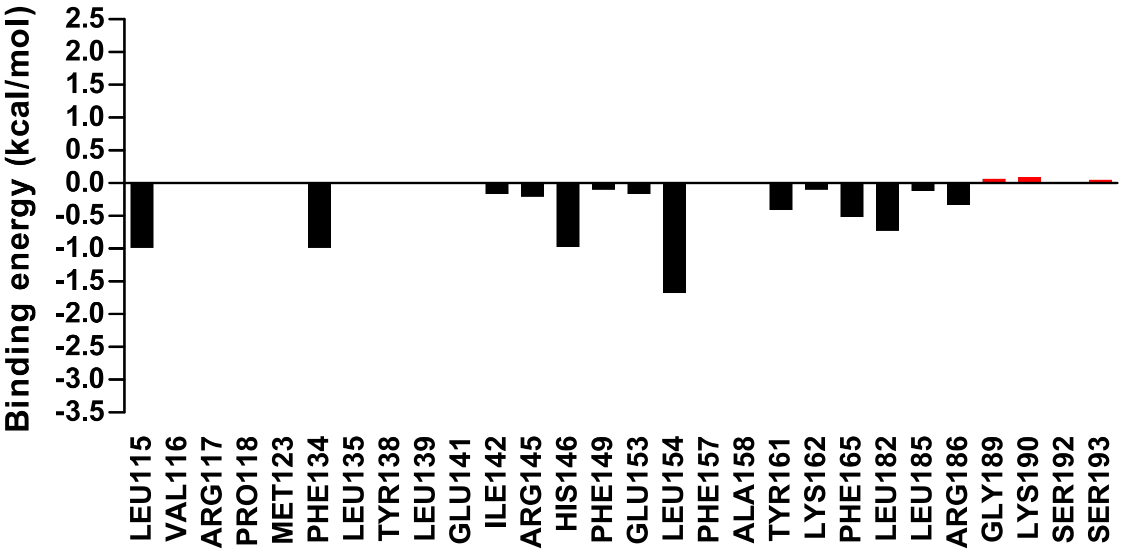

Figure S48. Binding energy decomposition for compound **3a**. Favorable interactions are depicted in black, while unfavorable interactions are depicted in red.

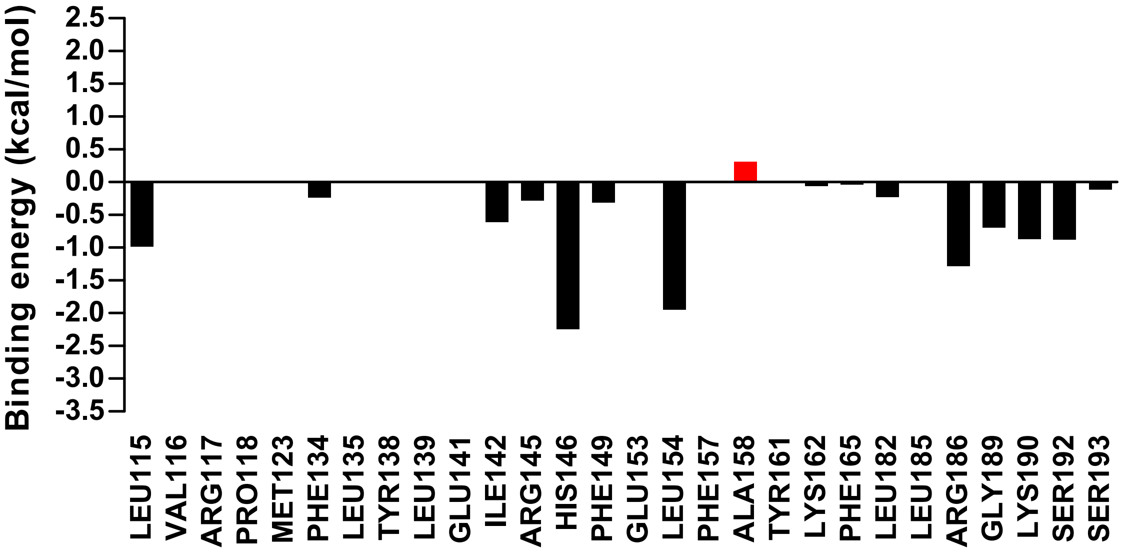

Figure S49. Binding energy decomposition for compound **3b**. Favorable interactions are depicted in black, while unfavorable interactions are depicted in red.

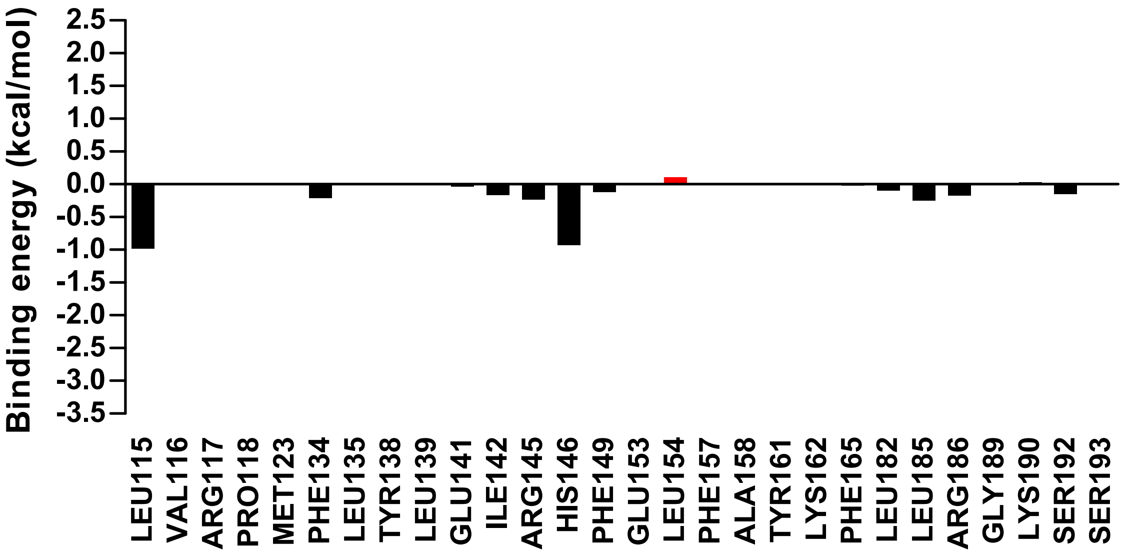

Figure S50. Binding energy decomposition for compound **3c**. Favorable interactions are depicted in black, while unfavorable interactions are depicted in red.

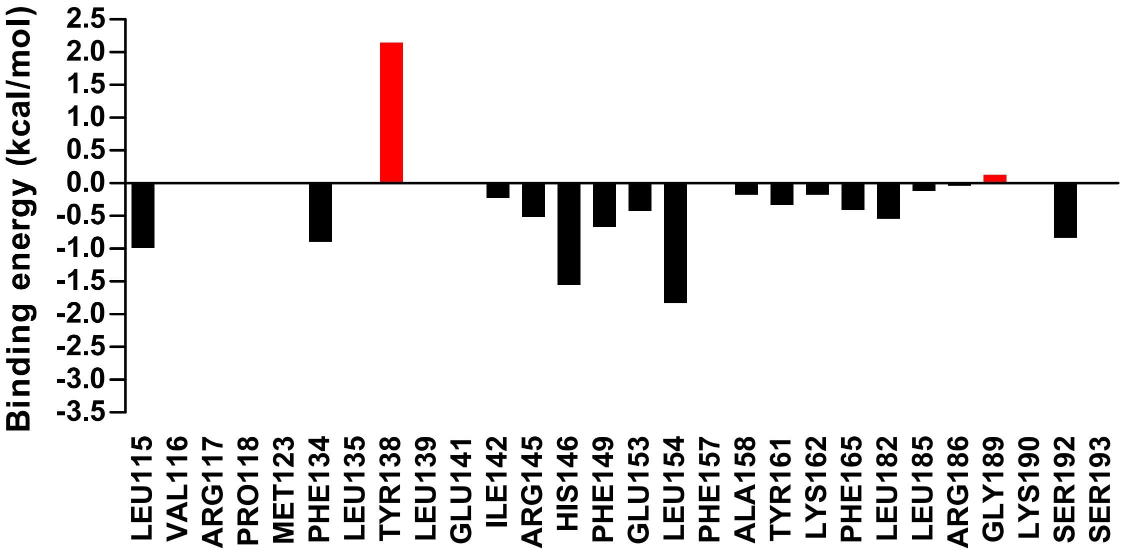

Figure S51. Binding energy decomposition for compound **3d**. Favorable interactions are depicted in black, while unfavorable interactions are depicted in red

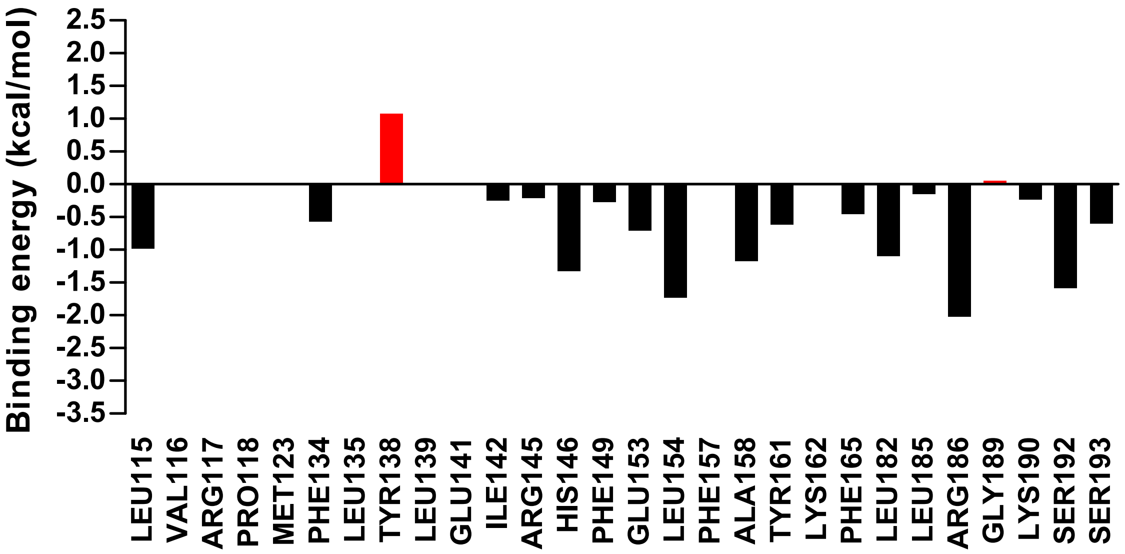

Figure S52. Binding energy decomposition for compound **3e**. Favorable interactions are depicted in black, while unfavorable interactions are depicted in red.

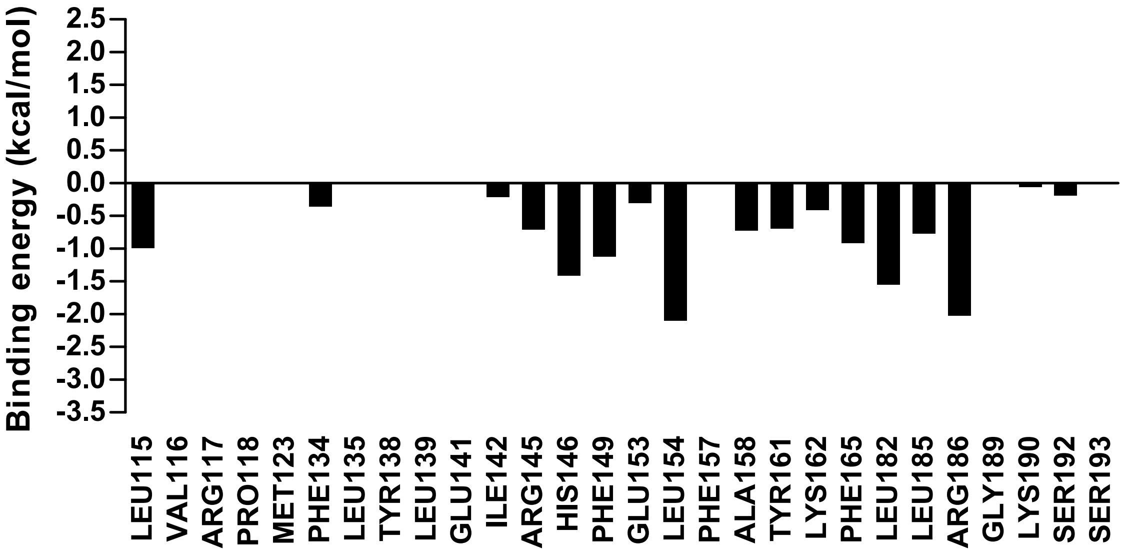

Figure S53. Binding energy decomposition for compound **3f**. Favorable interactions are depicted in black, while unfavorable interactions are depicted in red.

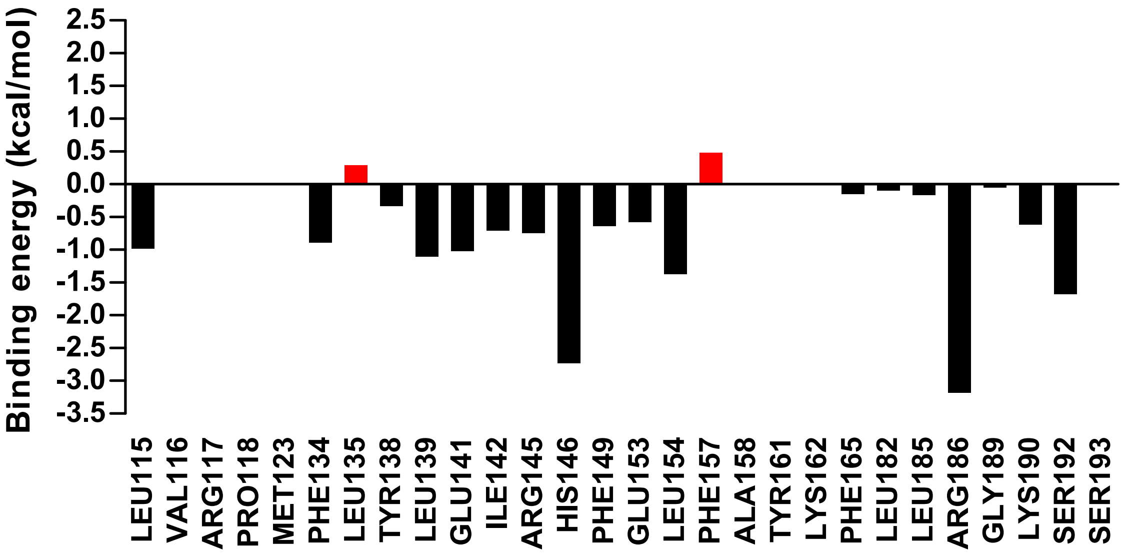

Figure S54. Binding energy decomposition for compound **3g**. Favorable interactions are depicted in black, while unfavorable interactions are depicted in red.

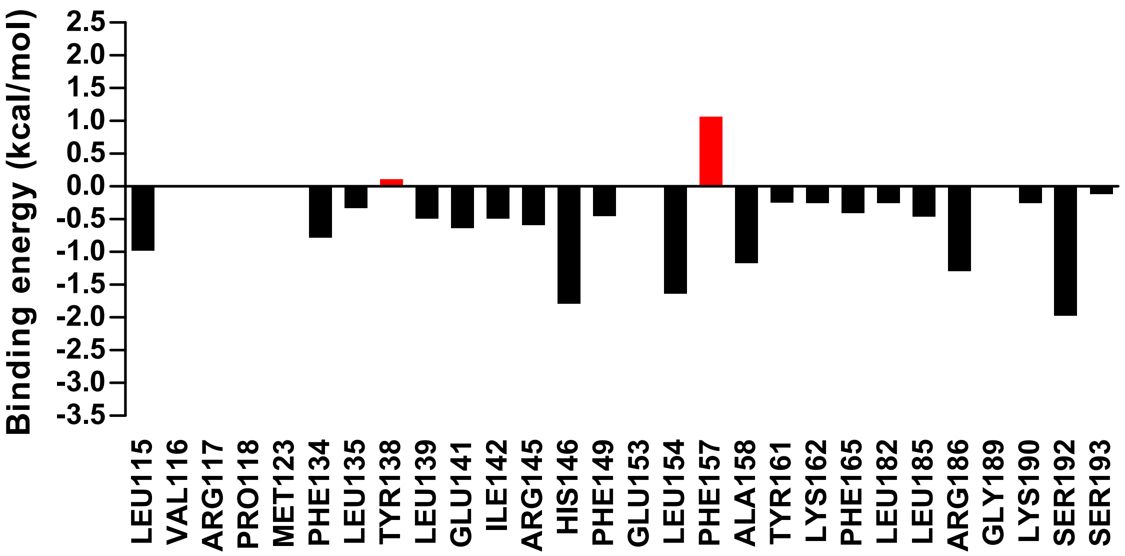

Figure S55. Binding energy decomposition for compound **3h**. Favorable interactions are depicted in black, while unfavorable interactions are depicted in red.

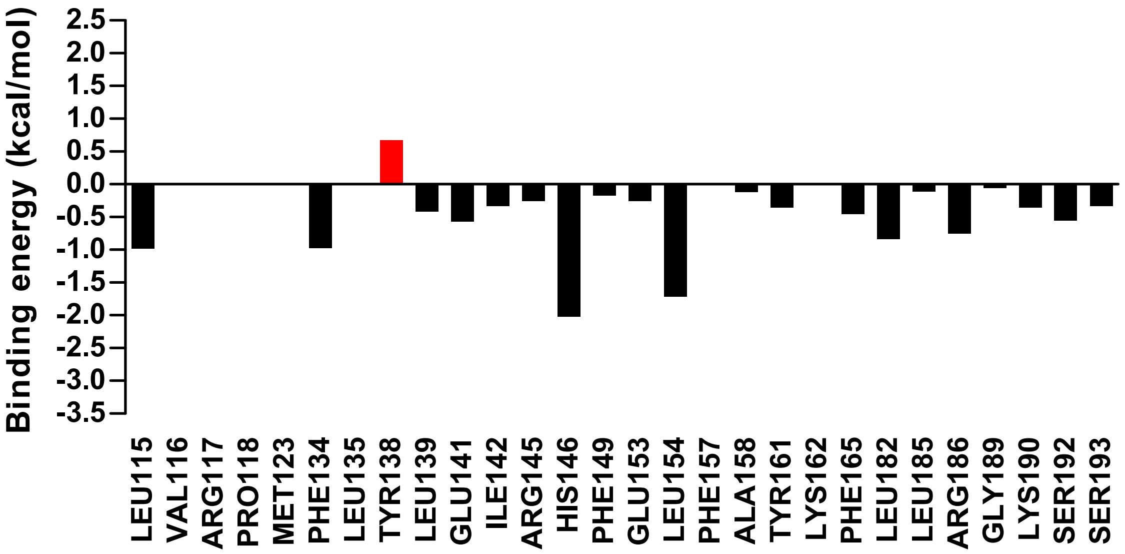

Figure S56. Binding energy decomposition for compound **3i**. Favorable interactions are depicted in black, while unfavorable interactions are depicted in red.
